# Divergent evolution of low-complexity regions in the vertebrate CPEB protein family

**DOI:** 10.3389/fbinf.2025.1491735

**Published:** 2025-03-20

**Authors:** Serena Vaglietti, Stefania Boggio Bozzo, Mirella Ghirardi, Ferdinando Fiumara

**Affiliations:** “Rita Levi-Montalcini” Department of Neuroscience, University of Turin, Turin, Italy

**Keywords:** cytoplasmic polyadenylation element-binding protein, CPEB proteins, liquid-liquid phase separation (LLPS), prion-like proteins, low-complexity regions (LCRs), homopolymeric amino acid repeats, divergent evolution, paralogous proteins

## Abstract

The *cytoplasmic polyadenylation element-binding proteins* (CPEBs) are a family of translational regulators involved in multiple biological processes, including memory-related synaptic plasticity. In vertebrates, four paralogous genes (CPEB1-4) encode proteins with phylogenetically conserved C-terminal RNA-binding domains and variable N-terminal regions (NTRs). The CPEB NTRs are characterized by low-complexity regions (LCRs), including homopolymeric amino acid repeats (AARs), and have been identified as mediators of liquid-liquid phase separation (LLPS) and prion-like aggregation. After their appearance following gene duplication, the four paralogous CPEB proteins functionally diverged in terms of activation mechanisms and modes of mRNA binding. The paralog-specific NTRs may have contributed substantially to such functional diversification but their evolutionary history remains largely unexplored. Here, we traced the evolution of vertebrate CPEBs and their LCRs/AARs focusing on primary sequence composition, complexity, repetitiveness, and their possible functional impact on LLPS propensity and prion-likeness. We initially defined these composition- and function-related quantitative parameters for the four human CPEB paralogs and then systematically analyzed their evolutionary variation across more than 500 species belonging to nine major clades of different stem age, from Chondrichthyes to Euarchontoglires, along the vertebrate lineage. We found that the four CPEB proteins display highly divergent, paralog-specific evolutionary trends in composition- and function-related parameters, primarily driven by variation in their LCRs/AARs and largely related to clade stem ages. These findings shed new light on the molecular and functional evolution of LCRs in the CPEB protein family, in both quantitative and qualitative terms, highlighting the emergence of CPEB2 as a proline-rich prion-like protein in younger vertebrate clades, including Primates.

## Introduction

The *cytoplasmic polyadenylation element-binding proteins* (CPEBs) are a family of RNA-binding proteins regulating mRNA translation (Richter, 2007) involved in various cellular processes, ranging from translational activation in oocytes to the control of local protein synthesis in memory-related synaptic plasticity (Richter, 2007; Kandel, 2012; Huang et al., 2023), also through prion-like mechanisms (Si et al., 2003a; Si et al., 2003b; Stephan et al., 2015). CPEBs have also been implicated in the pathogenesis of several diseases, ranging from cancer to post-traumatic stress disorder (PTSD) and autism spectrum disorders (ASDs; Kozlov et al., 2021; Lu et al., 2021).

In vertebrates, four paralogous genes encode a family of proteins (CPEB1-4) each made of a conserved C-terminal region (CTR), with two RNA-recognition motifs (RRMs) and a zinc finger (ZnF) domain, and an N-terminal region (NTR) characterized by low-complexity regions (LCRs), including homopolymeric amino acid repeats (AARs), that vary quite extensively across CPEB paralogs and orthologs (Wang and Cooper, 2010; Fiumara et al., 2010). At the functional level, CPEBs can act both as repressors and activators of mRNA translation (Richter, 2007), switching between these two states through paralog-specific mechanisms, like phosphorylation or prion-like structural transitions (Si et al., 2003a; Majumdar et al., 2012; Stephan et al., 2015). The prion-like switch relies on a structural transition from a soluble to a fibrillary form enriched in β-sheets and/or coiled-coil structures in different CPEB orthologs (Fiumara et al., 2010; Kandel, 2012; Kandel et al., 2013; Raveendra et al., 2013; Cervantes et al., 2016; Hervas et al., 2020; Hervas et al., 2021; Reselammal et al., 2021; Bowler et al., 2022). These self-sustaining prion-like transitions have been attributed to LCRs, or ‘prion-like’ domains (PrDs), in the NTRs of these proteins, (Si, 2015; Si et al., 2003a; Heinrich and Lindquist, 2011; Stephan et al., 2015; Hervas et al., 2020; Hervas et al., 2021; Reselammal et al., 2021). More recently, different CPEB orthologs have been shown to undergo liquid-liquid phase separation (LLPS; Ford et al., 2019; Ford et al., 2023; Ashami et al., 2021; Duran-Arqué et al., 2022; Ramírez de Mingo et al, 2022; Ramírez de Mingo et al., 2023), a biophysical process by which proteins assemble into transient ‘condensates’ (e.g., Vaglietti et al., 2023). Notably, the ability of CPEB proteins to undergo LLPS has been also attributed to their N-terminal LCRs (Duran-Arqué et al., 2022; Ramírez de Mingo et al., 2023).

CPEB genes appeared in Metazoa (Paps and Holland, 2018). The four vertebrate genes originated from an ancestral one by duplication (Duran-Arqué et al., 2022; Rouhana et al., 2023) and are divided into the CPEB1 and CPEB2-4 subfamilies based on sequence similarity (Hake and Richter, 1994; Kurihara et al., 2003; Theis et al., 2003). While sharing fundamental features, the four CPEBs diverged functionally in several respects, including mRNA binding modes, activation mechanisms, and subcellular localization (Duran-Arqué et al., 2022; Huang et al., 2023). The CPEB CTRs display a considerable degree of conservation (Richter, 2007), suggesting that the evolution of the variable NTRs may have substantially contributed to the functional diversification of the four CPEB paralogs. Indeed, the paralog-specific LLPS and prion-like behaviors of CPEBs rely on their variable NTRs (Stephan et al., 2015; Duran-Arqué et al., 2022), consistent with the fact that changes in LCRs/AARs composition and length can alter LLPS propensity and prion-like behavior (Fiumara et al., 2010; Vaglietti et al., 2023). Therefore, the emergence of paralog-specific CPEB functions (neo-/sub-functionalization) may have derived from at least two mechanisms, i.e., gene duplication and LCR divergence, whose interplay has key roles in genome evolution (Persi et al., 2016), promoting the functional divergence of proteins, including nucleic-acid binding proteins (Radò-Trilla et al., 2015; Chiu et al., 2022).

The discovery of the ability of the CPEB NTRs to drive both LLPS and prion-like aggregation raised several new biological questions. In general terms, the functional and temporal relationships between the transient LLPS-driven condensation and the persistent, prion-like fibrillization of CPEBs are still not well defined. Ford et al. (2019) proposed that CPEB3 is in the repressive state within LLPS-driven condensates and activates translation upon prion-like fibrillization. However, other groups identified LLPS as a precursor, rather than an alternative state to prion-like fibrillization (Ashami et al., 2021; Ramírez de Mingo et al., 2023). At the molecular level, it is unclear which compositional and structural features of the NTRs are related to their ability to drive LLPS and prion-like conformational and functional changes. The primary sequence composition and complexity of both LLPS-prone LCRs and PrDs in proteins have been related to their functional behaviors. ‘Molecular grammars’, which are still not clearly understood, are thought to specify sequence/function relationships in these regions (Wang et al., 2018; Saar et al., 2021; Rekhi et al., 2024). Therefore, a qualitative and quantitative definition of the key features of the primary sequences of LLPS-prone and prion-like CPEB LCRs may help to better understand their functional properties. Furthermore, whether or not the same portions of the CPEB NTRs drive both LLPS and prion-like structural transitions remains to be defined. Ramírez de Mingo et al. (2023) proposed that the CPEB3 NTR contains one prion-like portion and another one driving LLPS. However, in contrast with this model, the latter region had been identified by Stephan et al. (2015) as a functional prion-like region. Finally, it is unclear how evolutionary changes in the LCR primary sequences may have contributed to the functional divergence of the four CPEB paralogs once they had appeared in vertebrates.

To address these issues, we systematically defined the amino acid composition, sequence complexity, LLPS-propensity, and prion-likeness of the four human CPEBs, in both quantitative and qualitative terms, and traced the evolutionary history of these parameters in the CPEB orthologs across vertebrate clades.

## Results

### Differential amino acid occurrence and distribution between the human CPEB paralogs

**FIGURE 1:**
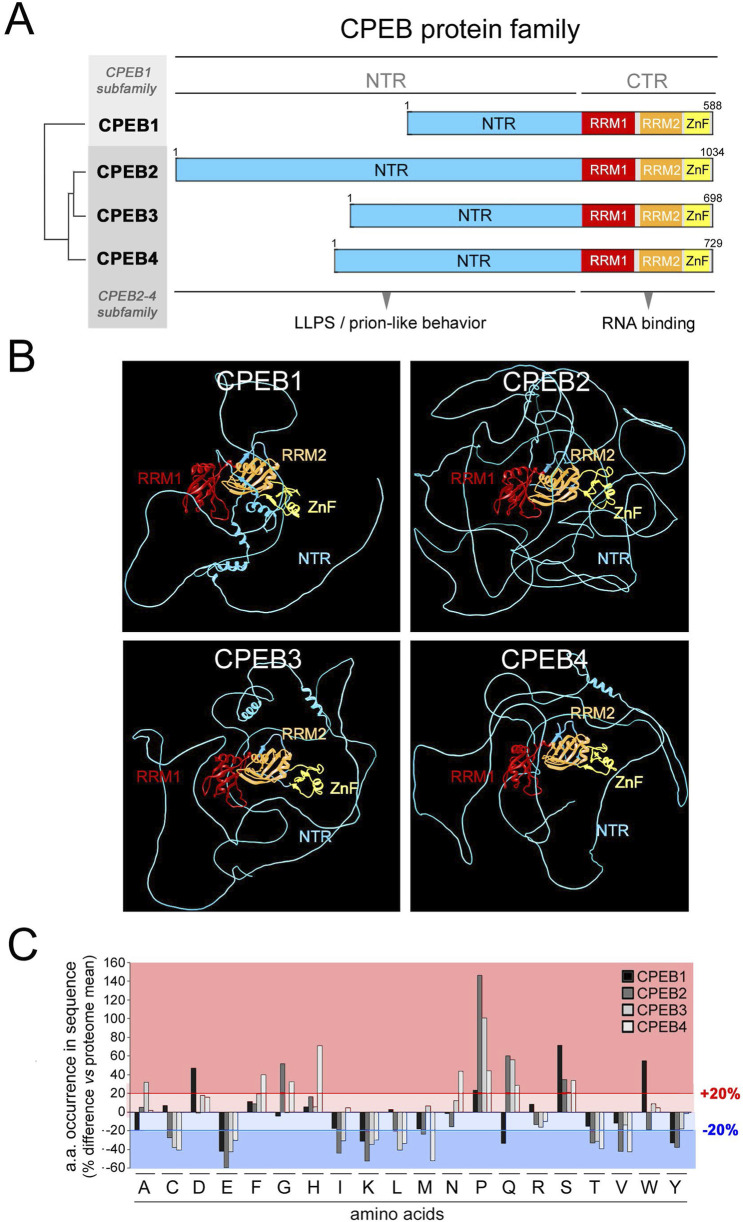
Differential occurrence of amino acids between CPEB paralogs. **(A)** Schematic representation of the domain structure of the human CPEB1-4 protein family, highlighting (tree on the *left*) the similarity relationships between the four paralogous proteins (as determined using Multalin), which are grouped into the CPEB1 and CPEB2-4 subfamilies. The RNA recognition domains motifs 1 and 2 (RRM1 and RRM2) in the C-terminal region (CTR) are highlighted in *red* and *orange*, respectively, and the zinc finger domain (ZnF) in *yellow*. The more variable N-terminal region (NTR) is in *cyan*. **(B)** Atomic-level structural models of the human CPEB1-4 proteins generated by AlphaFold2 based on their primary sequences. Protein domains are colored as in **(A)**. **(C)** Bar graph reporting the percent occurrence of each amino acid in the primary sequence of each CPEB paralog relative to the mean occurrence of each amino acid across all proteins of the human proteome (CPEB1 in *black*, CPEB2 in *dark gray*, CPEB3 in *gray* and CPEB4 in *light gray*). The *red lines* highlight deviations >20% in either direction from the proteome values.

**FIGURE 2:**
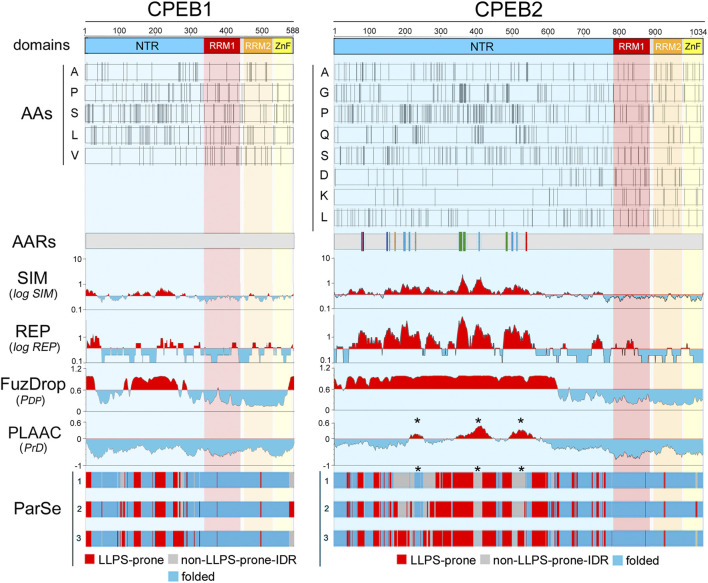
Amino acid distributions and per-residue scores related to sequence complexity, LLPS propensity, and prion-likeness along the primary sequences of human CPEB1 and CPEB2. The *upper bars* (“domains”) display a schematic representation of the domain structure of human CPEB1 and CPEB2, as in Figure 1A. RRM1 (a.a. 336-438 in CPEB1, a.a. 775-871 in CPEB2) is in *red*, RRM2 (a.a. 449-530 in CPEB1, a.a. 882-963 in CPEB2) is in *orange*, and the ZnF (a.a 532-581 in CPEB1, a.a. 967-1021 in CPEB2) is in *yellow.* The *bars* below (“AAs”) display the distribution of the indicated amino acids in CPEB1 and CPEB2 as *thin vertical line segments* (see Supplementary Figures S1–S2 for other amino acids). Note how some of these amino acids tend to concentrate in the NTR (e.g., A, P, S, L in CPEB1, A, G, P, Q, S in CPEB2) and others in the RNA-binding CTR (e.g., V in CPEB1, D and K in CPEB2). The *gray bars* (“AARs”) represent schematic representations of the two proteins with colored vertical bars indicating the position of AARs (≥4 residues; Pelassa et al., 2019). Note how CPEB1 is devoid of AARs while CPEB2 contains many of them formed by different amino acids, i.e., polyA (*red*), polyC (*gray*), polyG (*green*), polyP (*turquoise*), polyQ (*orange*), and polyS (*blue*). The *four plots* below report the per-residue scores related to sequence simplicity (‘SIM’), repetitiveness (‘REP’), LLPS propensity (FuzDrop pDP score’), and prion-likeness (PLAAC PrD score’). The SIM and REP scores are plotted on a logarithmic scale. In the SIM and REP plots, *red* and *cyan peaks* highlight protein regions with scores above or below, respectively, the mean value of the two parameters across the proteins of the whole human proteome. In the FuzDrop plot, *red* and *cyan peaks* highlight protein regions with P_DP_ scores above or below, respectively, the prediction threshold (P_DP_ ≥ 0.60) for LLPS-prone regions (Vendruscolo and Fuxreiter, 2022). In the PLAAC score plot, *red* and *cyan peaks* highlight protein fragments with scores above or below, respectively, the prediction threshold (PrD score ≥0) for prion-like regions (Lancaster et al., 2014). Note how the most of the CPEB2 NTR has a low-complexity (high SIM score) and repetitive (high REP score) primary sequence which is predicted by FuzDrop to be LLPS-prone for the most part. PLAAC identifies three discrete prion-like regions (*asterisks*) in the more central portion of the NTR. Conversely, the CPEB1 NTR contains some LCRs with relatively low SIM and REP scores. Fuzdrop identifies limited portions of the NTR as LLPS-prone and no prion-like domain is identified by PLAAC. The *tree bars* at the bottom indicate the position of residues identified by ParSe as part of LLPS-prone (P; *red*) or not (D; *gray*) LCRs/IDRs, or as part of folded (F; *turquoise*) regions based on three different algorithms (labelled here as 1, 2, and 3; see Methods; Ibrahim et al., 2023). Note how, in comparisons with the FuzDrop predictions, the LLPS-prone regions identified by ParSe in the NTR are less extended in both proteins. It is also remarkable how the three PrDs predicted by PLAAC in CPEB2 fall within regions with no LLPS propensity in two or three of the ParSe predictions (*asterisks*).

**FIGURE 3:**
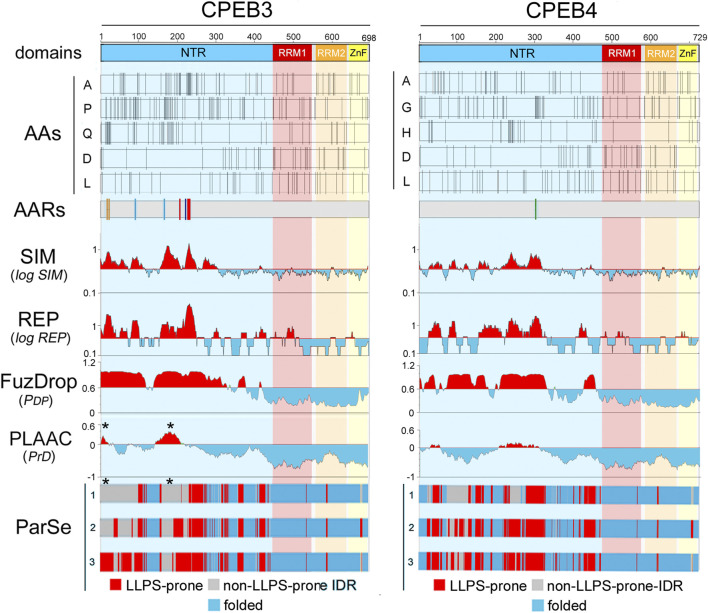
Amino acid distributions and per-residue scores related to sequence complexity, LLPS propensity, and prion-likeness along the primary sequences of human CPEB3 and CPEB4 **(A, B)** As in Figure 2, but for CPEB3 (RRM1: a.a. 439-535, RRM2: a.a. 546-627, ZnF: a.a. 631-685), and CPEB4 (RRM1: a.a. 470-566, RRM2: a.a. 577-658, ZnF: a.a. 662-716). Note how some residues concentrate in the NTRs (A, P, and Q in CPEB3; A, G. and H in CPEB4) and others in the CTRs (D and L in both proteins). Plots for all amino acids are in Supplementary Figure S3–S4). Multiple AARs are present in CPEB3 (polyA, polyP, polyQ, polyS; color coding as in Figure 2), while CPEB4 contains only a short polyG tract. Both proteins display multiple NTR subregions with high SIM and REP scores and LLPS propensity as predicted by FuzDrop. The ParSe predictions are more conservative and identify multiple LLPS-prone subregions within the two NTRs. PLAAC predicts two prion-like regions in both proteins, but with relatively high PrD scores in CPEB3 and with only borderline scores in CPEB4. Note how the CPEB3 PrDs predicted by PLAAC (*asterisks*) fall within regions with no LLPS propensity in two or three of the ParSe predictions (*asterisks*).

We initially performed a systematic compositional analysis of the four human CPEB paralogs (Figures 1A, B), defining for each protein the percent occurrence of the 20 amino acids (Figure 1C) and their distribution along its primary sequence (Figures 2, 3; Supplementary Figures S1–S4).

The percent occurrence of each amino acid in each CPEB protein was compared to its mean percent occurrence across all human proteins (Figure 1C). This analysis revealed that, in one or more of the four CPEBs, the occurrence of several amino acids substantially deviates (>20% over-/under-representation) from their occurrence in the human proteome. Deviations of this magnitude in the percent occurrence of a given amino acid in a protein can be related to the presence of compositionally biased protein regions (LCRs/AARs) even of modest length (see Methods). All four CPEBs display an underrepresentation of charged residues like glutamate (E) and lysine (K), and an overrepresentation of proline (P) and serine (S). Compositional differences across paralogs were found mostly between CPEB1 and CPEB2-4, but also between members of the CPEB2-4 subfamily. For example, glutamine (Q) residues are underrepresented in CPEB1 and overrepresented in CPEB2-4, and the degree of P overrepresentation is very different in CPEB2 (+146% vs. proteome), CPEB3 (+100%), and CPEB4 (+44%).

In analyzing the distribution of the 20 amino acids (Figures 2, 3; Supplementary Figures S1–S4), we found that some of them display a non-homogenous patterning along the CPEB primary sequences. For instance, P and Q residues are concentrated within the NTRs of CPEB2-4. These asymmetric distributions were particularly evident in CPEB2, with many amino acids concentrated in the NTR (e.g., G, S, and A, besides P and Q) and some others more abundant in the CTR (e.g., aspartate, D).

These asymmetries in amino acid distribution across protein regions are also related to the presence of AARs in the NTRs of CPEB2-4 (Figures 2, 3), which are absent in CPEB1. AARs are numerous in CPEB2/3, but almost absent in CPEB4, which bears only a short polyglycine (polyG) tract. CPEB2/3 both contain poly-alanine (polyA), -glutamine (polyQ), -proline (polyP), and -serine (polyS) repeats. CPEB2 also contains multiple polyG repeats.

### Differential sequence simplicity and repetitiveness between the human CPEB paralogs

To define quantitatively the complexity of the primary sequence of CPEBs, we calculated two per-residue scores expressing the local degree of sequence simplicity (SIM) and repetitiveness (REP) in a sliding window of 20 residues around each residue of the four proteins. The lesser the number of different amino acids is in the 20-residue window, the higher is the SIM score. This score would be minimum for a sequence with 20 different amino acids (i.e., ‘ACDEFGHIKLMNPQRSTVWY’ in any order) and maximum for a homopolymeric AAR (e.g., ‘AAAAAAAAAAAAAAAAAAAA’). Given a certain degree of complexity, the REP score quantifies primary sequence repetitiveness. Thus, in a region formed by 10 A and 10 Q residues, the score would be lower for ‘AQAQAQAQAQAQAQAQAQAQ’, intermediate for ‘AAAAAQQQQQAAAAAQQQQQ’, and higher for ‘AAAAAAAAAAQQQQQQQQQQ’.

The two scores are higher for CPEB2-4 in comparison with CPEB1 (Figures 2, 3). CPEB2-4 proteins display a tripartite organization in terms of complexity, with SIM and REP scores that are higher in the proximal two-thirds of the NTD, intermediate in its distal third, and lower in the CTR (Figures 2, 3).

### Differential LLPS propensity and prion-likeness between the human CPEB paralogs

The previous findings prompted us to test whether the observed differences in the composition and complexity of the CPEB NTRs may impact their LLPS propensity and prion-likeness using the FuzDrop, ParSe, and PLAAC algorithms (Vendruscolo and Fuxreiter, 2022; Ibrahim et al., 2023; Lancaster et al., 2014). These well-established prediction tools can provide nuanced per-residue predictions (see Methods) that can help identify LCR subregions specifically involved in driving LLPS-driven condensation and/or prion-like aggregation. Some of these tools were previously used to characterize CPEB3 (Ramírez de Mingo et al., 2023).

Both FuzDrop and ParSe identified the NTRs of all four CPEBs as LLPS-prone regions (Figures 2, 3), consistent with experimental evidence that all CPEB paralogs undergo LLPS or are recruited to LLPS-driven compartments (Duran-Arqué et al., 2022). For CPEB1, which is recruited to LLPS-driven ribonucleoprotein particle (RNP) condensates (Duran-Arqué et al., 2022; Ford et al., 2023), the FuzDrop and ParSe predictions were essentially overlapping. For CPEB2-4, both algorithms predicted LLPS-prone regions mostly confined to the two proximal thirds of the NTRs, which also have the highest SIM and REP scores. However, while FuzDrop identified most of these initial LCR portions of CPEB2-4 as LLPS-prone, ParSe was able to identify within them smaller, discrete subregions with LLPS propensity (Figures 2, 3).

PLAAC predicted prion-like domains (PrDs) in the central thirds of CPEB2-4, as well as in the N-terminal portion of CPEB3 (Figures 2, 3). No PrD was predicted in CPEB1 (Figure 2), consistent with previous experimental observations (Si et al., 2010). The predicted PrDs in CPEB2-4 are comprised within the extended LLPS-prone regions identified by FuzDrop, which may indicate that they mediate both LLPS and fibrillization. However, the discrete LLPS-prone subregions predicted by ParSe for CPEB2/3 appeared to alternate with the PLAAC PrDs, strongly suggesting that neighboring NTR subregions are alternatively implicated in either LLPS-driven condensation or in prion-like fibrillization.

Together with our previous analyses, these findings indicate that the four CPEB NTRs are formed by compositionally different subregions with distinct structural and functional roles, in agreement with initial evidence available for the CPEB3 paralog (Stephan et al., 2015; Ramírez de Mingo et al., 2023).

### An evolutionary perspective on the LCRs of the vertebrate CPEB protein family

The previous findings prompted us to test whether the primary sequence features of the four human CPEBs that we highlighted are phylogenetically conserved, or they gradually arose in the evolutionary history of vertebrates, or they represent instead highly variable taxon-/species-specific molecular features. Thus, we explored how the composition, complexity, LLPS propensity, and prion-likeness of the four CPEB paralogs have evolved in the gnathostome vertebrate lineage.

**FIGURE 4:**
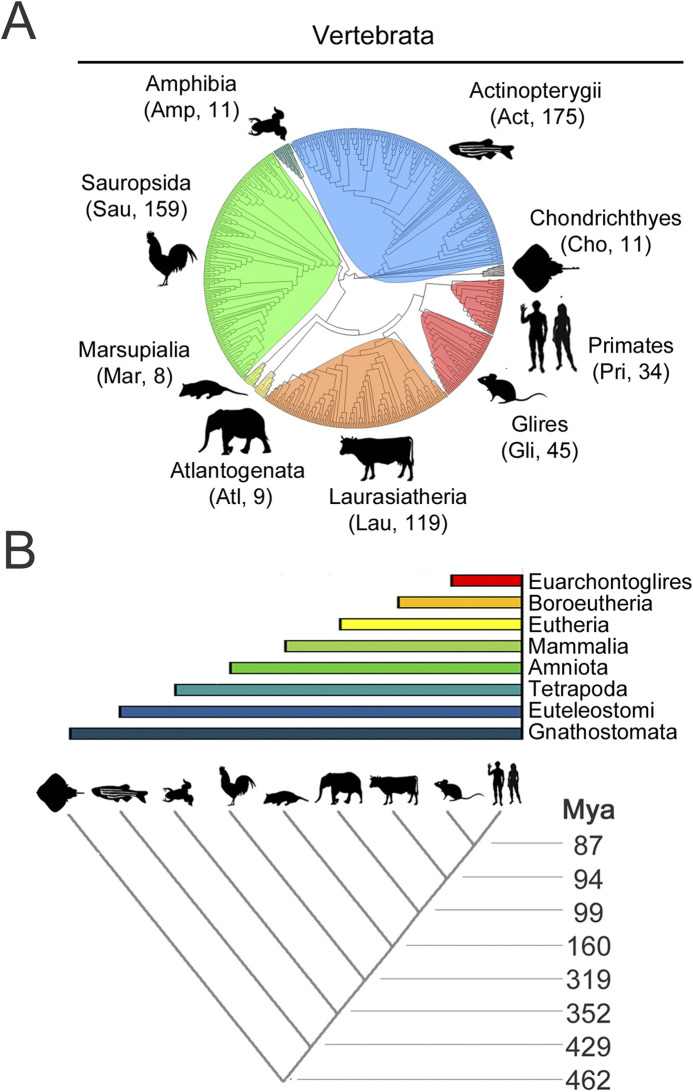
Evolutionary relationships of vertebrate species and clades whose CPEB ortholog protein sequences were analyzed **(A)**. Phylogenetic tree of the 571 species, belonging to the indicated major vertebrate clades, highlighted in different color shades, whose CPEB1-4 primary sequences were analyzed. A three-letter abbreviation of the clade name and the number of species with available CPEB sequences are indicated for each clade *in brackets*, with silhouette drawings indicating a representative species for each clade, i.e., *Homo sapiens* for Primates (Pri), *Mus musculus* for Glires (Gli), *Bos taurus* for Laurasiatheria (Lau), *Loxodonta africana* for Atlantogenata (Atl), *Monodelphis domestica* for Marsupialia (Mar), *Gallus gallus* for Sauropsida (Sau), *Xenopus tropicalis* for Amphibia (Amp), *Danio rerio* for Actinopterygii (Act), and *Amblyraja radiata* for Chondrichthyes (Cho). **(B)** The lower cladogram illustrates the phylogenetic relationships between the nine vertebrate clades shown in **(A)**. Clade stem ages are indicated *on the right*. The *colored bars* on top indicate the higher-level clades (listed *on the right*) that variably comprise the nine clades forming the lower cladogram.

For each CPEB paralog, we selected for this analysis hundreds of orthologs from species belonging to nine major clades of different stem age in the evolutionary tree of the vertebrate lineage (Figure 4A; Supplementary Table S1), from older ones, like Chondrichthyes and Actinopterygii, to younger ones like Glires and Primates (Figure 4B). The stem ages of these clades range from ∼87 to ∼462 million years ago (mya; Figure 4B). Besides Primates (*Pri*, 34 species) and their sister taxon Glires (*Gli*, 45 species, including rodents, rabbits, hares, and pikas) within Euarchontoglires (87 mya), the clades are Laurasiatheria (*Lau*, 119 species (94 mya), including carnivorans, and even-/odd-toed ungulates), Atlantogenata (*Atl*, 9 species (94 mya), comprising species from afrotherian (e.g., elephants), and xenarthran (e.g., armadillos) orders, Marsupialia (*Mar*, 8 species, 99 mya), Sauropsida (*Sau*, 159 species (180 mya), including birds and reptiles), Amphibia (*Amp*, 11 species, 319 mya), and bony (Actinopterygii, *Act*, 175 species, 429 mya) or cartilagineous (Chondrichthyes, *Cho*, 11 species, 462 mya) fishes.

**FIGURE 5:**
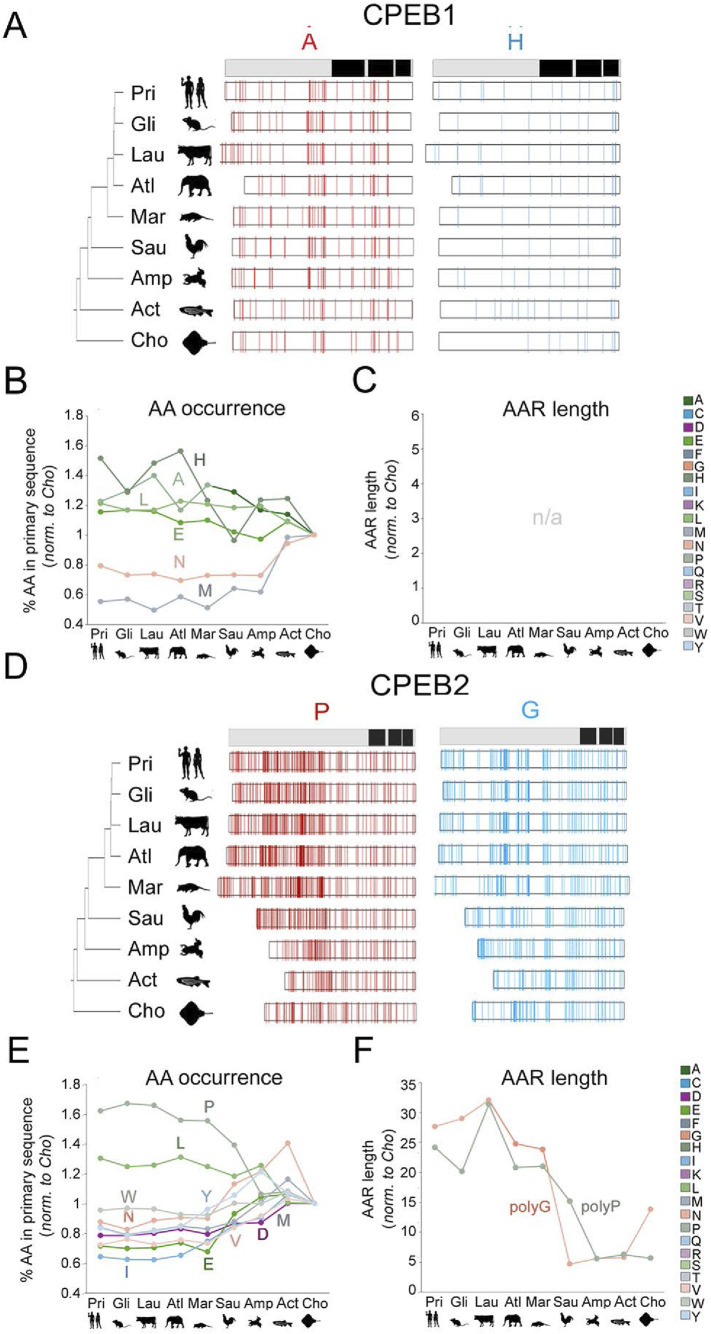
Evolution of amino acid occurrence and AAR lengths in vertebrate CPEB1/2 **(A)** The bar *on top* is a simplified representation of the domain structure of human CPEB1 (RRMs and ZnF are in *black*; see Figure 2A). On the *left*, phylogenetic tree of the 9 vertebrate taxa that were analyzed with silhouettes of representative species (as listed in the legend to Figure 4A). The *white bars* display the distribution of A and H residues, represented as *thin vertical line segments* (in *red* and *cyan*, respectively). Along the primary sequence of CPEB1 orthologs in the indicated species. The ortholog bars were graphically aligned to the junction between the NTR and CTR. **(B)** Graph reporting the mean percent occurrence of the indicated amino acids across the ortholog CPEB1 proteins of each clade. Values are normalized to those found in the clade with the oldest stem age (Chondrichthyes). The graph only reports the values relative to those amino acids whose evolutionary variation in occurrence correlates significantly with clade sten ages, as reported in Supplementary Table S2. Glutamate (E), histidine (H), and leucine (L) display significant, clade stem age-related, increases in their percent occurrence along the vertebrate lineage, whereas asparagine (N) and methionine (M) display an opposite, significant trend. Clade specific oscillations of the analyzed values were not analyzed in detail. **(C)** As in **(B)**, for AARs lengths. As CPEB1 is devoid of AAR, the graph is reported here for comparison purposes with the other paralogs (see **(B)** and Figure 6). **(D)** As in **(A)** for CPEB2. Note the considerable increase in the occurrence of proline (P) residues (*red* thin bars) and the elongation of polyG repeats (although the occurrence of G residues did not significantly increase overall). **(E)** As in **(B)** for CPEB2. Note how, along the vertebrate lineage, the occurrence of many amino acids increased or decreased significantly (see Supplementary Table S2). **(F)** As in **(C)** for CPEB2. Note how the total length of polyP and polyG stretches significantly increased from older to younger vertebrate clades (see Supplementary Table S2).

**FIGURE 6:**
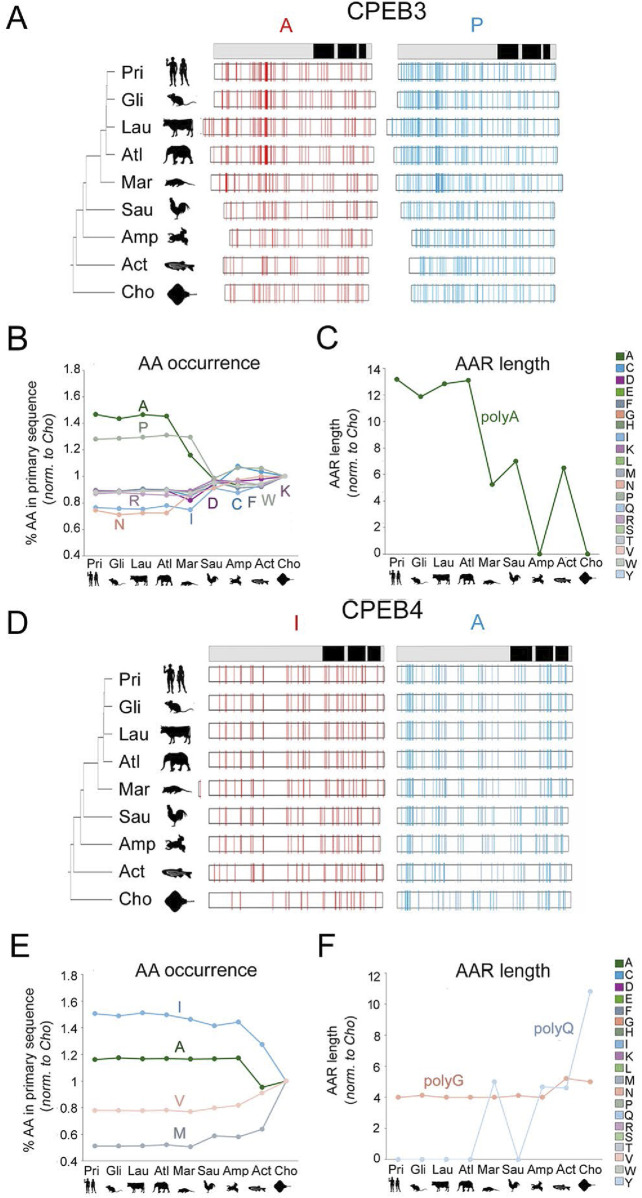
Evolution of amino acid occurrence and AAR lengths in vertebrate CPEB3/4 **(A–C)** As in Figure 5 for CPEB3. Note the significant increase in the occurrence of A and P residues along the vertebrate lineage. The increase in A residues is paralleled by an increase in total polyA repeat length. **(D–F)** As in Figure 5 for CPEB4. Note the disappearance of the polyQ repeat going from older to younger vertebrate clades.

**FIGURE 7:**
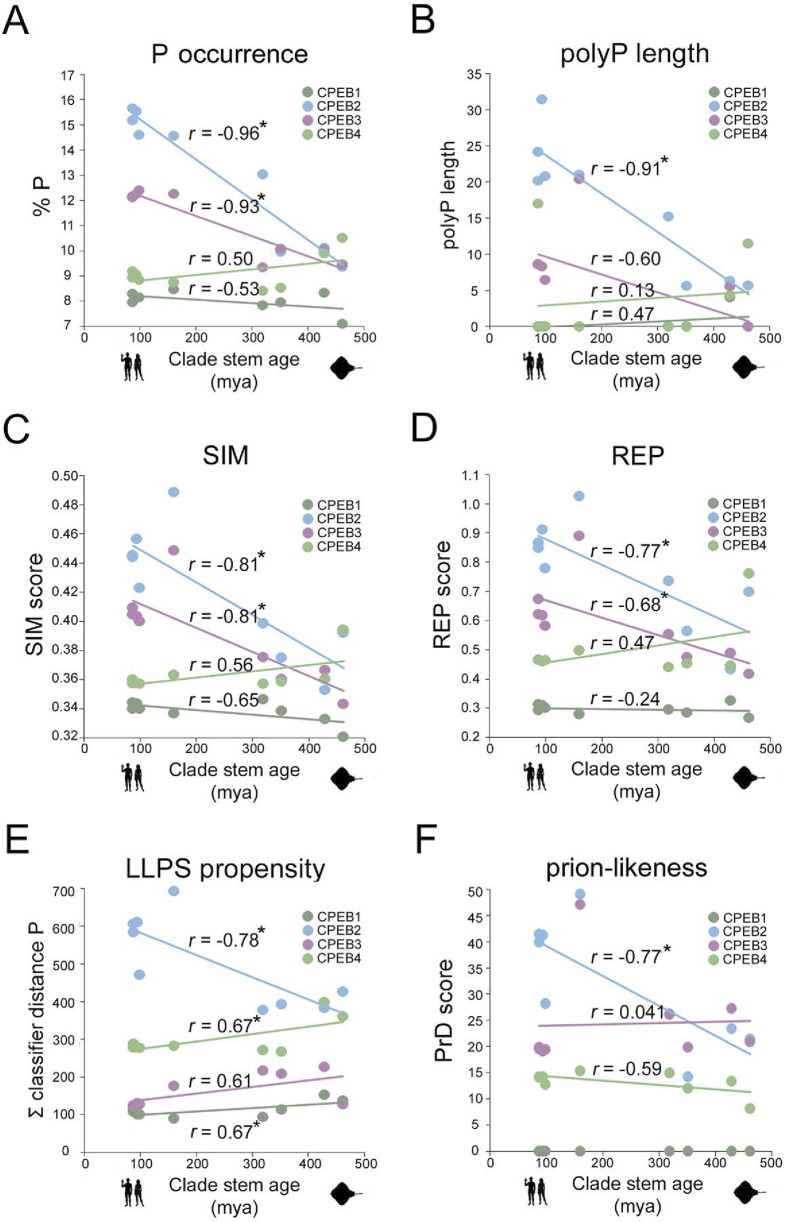
Divergent evolution of amino acid occurrence, sequence complexity, LLPS, and PrD propensity in CPEB1-4 **(A)**. Scatterplot with regression lines displaying, for each CPEB paralog, the correlation between the mean percent occurrence of P residue and stem ages across the nine clades. Statistically significant *r* correlation coefficients are marked with an *asterisk*. Data points in *dark green* for CPEB1, in *cyan* for CPEB2, in *purple* for CPEB3, and in *light green* for CPEB4. **(B–F)** As in **(A)** for polyP length **(B)**, SIM score **(C)**, REP score **(D)**, ParSe LLPS propensity score **(E)**, and PLAAC PrD score **(F)**.

**FIGURE 8:**
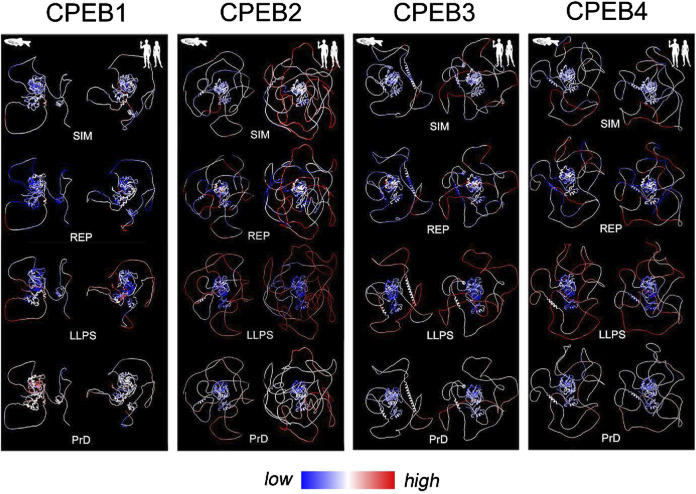
Evolutionary variation in SIM, REP, LLPS, and prion-likeness scores visualized onto structural models of *Danio rerio* and *Homo sapiens* CPEBs. For each CPEB paralog, the four panels display atomic-level structural models of the ortholog proteins of *Danio rerio* (*left column*) and *Homo sapiens* (*right column*) generated by AlphaFold2. Per-residue SIM, REP, LLPS, and prion-likeness scores are reported on protein structures using a pseudocolor scale going from *blue* (lower scores) to *red* (higher scores) through *white* (intermediate scores).

For each available CPEB primary sequence, we calculated the percent occurrence of the 20 amino acids, the total length of the repeats of each amino acid (AARs), as well as the mean SIM, REP, LLPS propensity (ParSe), and prion-likeness (PLAAC) scores across all residues of each protein (Figures 5–8). For each CPEB paralog in each clade, we calculated the mean values of the same parameters across all the available ortholog sequences or, in some analyses, across the orthologs from only five randomly selected species (see Methods).

### Divergent evolution of compositional features across CPEB paralogs in vertebrates

For each CPEB paralog, we initially analyzed the evolutionary variation of the amino acid composition and AAR lengths across the nine vertebrate clades, as shown in Figure 4. We initially calculated, across all available sequences in each clade, the mean percent occurrence of each amino acid and the mean total length of the AARs formed by each amino acid. Then, we studied whether these 40 parameters remained substantially stable across clades during vertebrate evolution or whether they varied, either in a clade-specific manner or with detectable overall trends related to clade stem ages (Figures 5–8; Supplementary Table S2).

This analysis revealed how the amino acid composition of CPEB1 remained overall quite stable across clades, from Chondrichthyes to Euarchontoglires, with relatively modest changes in amino acid occurrences. However, some clade stem age-related trends in the occurrence of certain amino acids were detected. Indeed, the mean percent occurrence of A, E, H and L residues (*r* = −0.69, *r* = −0.78, *r* = −0.75 and *r* = −0.73 respectively, n = 9 taxa, p < 0.05 in all instances) increases significantly from older to younger clades, while that of M and N residues decreased (*r* = 0.85, p < 0.01 and *r* = 0.72, p < 0.05 respectively, n = 9 in both instances; Figures 5A, B). AARs, which are not present in human CPEB1, were also not found in most of its vertebrate orthologs with the exception of a few clades in which short repeats (∼4-residue-long) can be sporadically observed. No AAR length displays significant evolutionary variation across clades (Figure 5C).

CPEB2 underwent marked compositional changes across clades, and the percent occurrence of several amino acids varied considerably, correlating with clade stem ages (Figures 5D, E). In particular, the occurrence of P and L residues significantly increased by ∼60% and ∼30% respectively (*r* = −0.96, n = 9, p < 0.001 and *r* = −0.85, n = 9, p < 0.01) going from Chondrichthyes to Euarchontoglires, whereas the occurrence of negatively charged (E,D), several hydrophobic (I,V,M,W,Y) and N residues decreased (*r* from −0.78 to −0.98, n = 9, *p* between 0.035 and 0.001). PolyP and polyG significantly increased their total lengths (Figure 5F; *r* = −0.91, n = 9, p < 0.01, and *r* = −0.87, n = 9, p < 0.02, respectively). Interestingly, also a short 4-residue-long polyC repeat appeared in Metatheria (*r* = −0.85, n = 9, p < 0.01).

CPEB3 also underwent considerable changes in amino acid occurrence and AAR length. Remarkably, some of these changes were parallel to those observed for CPEB2. Indeed, the occurrence of P residues increased (Figures 6A, B, *r* = −0.93, n = 9, p < 0.001), while that of D, I, and N residues decreased (*r* between −0.86 and −0.96, n = 9, *p* between 0.002 and 0.001) from Chondrichthyes to Euarchontoglires. In addition, the occurrence of A residues increased in CPEB3 (*r* = −0.93, n = 9, p < 0.01), also in relation to polyA length elongation, while that of R, K, W, and C residues decreased (*r* between 0.90 and 0.91, n = 9, *p* < 0.001; Figure 6C).

Unlike the two other members of the CPEB2-4 subfamily, CPEB4 displayed quite limited changes in amino acid occurrence and AAR length through the vertebrate lineage. The occurrence of I (*r* = −0.82, n = 9, p < 0.01) and A residues (*r* = −0.75, n = 9, p < 0.02) increased significantly going from older to younger clades, whereas that of V (*r* = 0.84, n = 9, p < 0.01) and M residues (*r* = 0.76, n = 9, p < 0.02) decreased (Figures 6D, E). AARs shortened or disappeared in CPEB4 along the vertebrate lineage (Figure 6F). Indeed, the polyG tract, i.e., the only AAR in human CPEB4, is longer in Chondrichthyes than in Euarchontoglires (*r* = −0.76, n = 9, p < 0.03) and a polyQ repeat that is present in Chondrichthyes is not found in the other clades (*r* = −0.75, n = 9, p < 0.03).

Taken together, these findings indicate that throughout vertebrate evolution, the four CPEB paralogs have been markedly diverging in terms of primary sequence composition. In quantitative terms, it is evident how CPEB1 varied overall to a considerably lesser degree than the paralogs of the CPEB2-4 subfamily and how, within the latter, CPEB2 and CPEB3 varied more than CPEB4. We also highlighted some parallel changes across some paralogs, especially for CPEB2 and CPEB3. For instance, the occurrence of P residues and the length of polyP repeats increased in both CPEB2 and CPEB3, but not in CPEB1/4, going from older to younger clades (Figures 7A, B), whereas that of negatively charged (D and E) and some polar (N) or aromatic (Y) residues significantly decreased. Alanine residues increased in both CPEB3 and CPEB4. The increase in I residues in CPEB4 paralleled the increase in a related aliphatic amino acid (L) in CPEB2. These findings uncover a remarkable degree of compositional divergence across CPEB paralogs, especially between the CPEB1 and CPEB2-4 subfamilies and, within the latter, between CPEB2/3 and CPEB4. It is noteworthy how CPEB2 and CPEB3, the two known prion-like paralogs, underwent several parallel changes.

### Divergent evolution of sequence complexity, LLPS-propensity, and prion-likeness in vertebrate CPEBs

Based on the previous results, we analyzed how the observed evolutionary changes in amino acid composition of the four CPEB paralogs may have impacted their overall primary sequence complexity, predicted LLPS propensity, and prion-likeness. Towards this aim, we calculated the mean values of complexity-related (SIM and REP) and function-related (ParSe P distance and PLAAC PrD) scores across orthologs in each clade and studied their variation profiles across clades (Figures 7C–F). To visually highlight the protein regions impacted by the evolutionary variations of the complexity- and function-related scores, their per-residue values were reported using a pseudocolor scale onto the available AlphaFold models of CPEB paralogs of two species from older, i.e., *Danio rerio* (Actinopterygii), and younger, i.e., *Homo sapiens* (Euarchontoglires), vertebrate clades (Figure 8).

For CPEB1, we found that both SIM and REP do not display marked oscillations across clades and have, overall, lower values in comparison with those of CPEB2-4 (Figures 7C, D). The protein does not to have any predicted PrD in vertebrates, as in *Homo* (Figure 7F), and displays the lowest LLPS propensity among the CPEB paralogs across clades (Figure 7E). Overall, the CPEB1 complexity- and function-related scores are generally lower in comparison with those of CPEB2-4 in each clade, displaying modest degrees of variation in relation to clade stem ages (Figures 2, 3). The minimal decline in LLPS propensity of the protein going towards younger clades is statistically significant (r = 0.67, n = 9; p < 0.05). Figure 8 (*first panel from the left*) highlights onto structural models the lack of substantial changes in CPEB1 complexity- and function-related parameters between *Danio* and *Homo*.

At the opposite, the primary sequence of CPEB2 displayed a considerable reduction in sequence complexity, with a strong increase in both the SIM and REP scores going from Chondrichthyes to Primates, which correlates significantly with clade stem ages (*r* = −0.81, n = 9 taxa, p = 0.01 and *r* = −0.77, n = 9 taxa, p < 0.02, respectively). Notably, these changes are also paralleled by significant increases in both LLPS propensity and prion-likeness (*r* = −0.77, n = 9 taxa, p < 0.02 in both instances). Figure 8 highlights onto structural models the marked increases in CPEB2 complexity- and function-related scores, as well as in the length of the NTR (see Figure 2), between species from older (*Danio*) and younger (*Homo*) clades.

As for CPEB2, the primary sequence of CPEB3 also underwent a reduction in sequence complexity with an increase in SIM score going from Chondrichthyes to Euarchontoglires, correlating significantly with clade stem ages (*r* = −0.81, n = 9 taxa, p < 0.01; Figure 7C). The REP score also displays a similar statistically significant trend (*r* = −0.68, n = 9 taxa, p < 0.05; Figure 7D). However, these two trends are not paralleled by significant increases in LLPS propensity and prion-likeness (Figures 7E,F), as found instead for CPEB2. Indeed, the PrD scores are instead substantially stable at a relatively high levels across vertebrate clades, with no evident correlation with clade stem ages (Figure 7F, *r* = −0.07, n = 9 taxa, p = 0.86), while LLPS propensity even declined to a certain extent, although this reduction did not significantly correlate with clade stem ages (Figure 7E; *r* = −0.61, n = 9 taxa, p = 0.08). Together, these findings indicate that, as for CPEB2, the overall sequence complexity of CPEB3 declines going towards younger vertebrate clades, although this may not directly translate into an increase in LLPS propensity and prion-likeness. Moreover, they also suggest that CPEB3 may have reached certain degrees of LLPS propensity and prion-likeness relatively early in the vertebrate lineage and has maintained them ever since. Figure 8 visually illustrates the differences in CPEB3 SIM, REP, LLPS, and prion-likeness scores between *Danio* and *Homo*.

CPEB4 displayed quite different trends in comparison with CPEB2/3. Indeed, this protein underwent only a modest reduction in both the SIM and REP scores going towards younger clades, which did not significantly correlate with clade stem ages (*r* = 0.56 and *r* = 0.47, respectively, n = 9, *p* > 0.05 in both cases; Figures 7C,D), It is noteworthy that CPEB2, CPEB3, and CPEB4 had similar SIM and REP scores in the older vertebrate clades and then diverged considerably along the vertebrate lineage. In contrast with what was found for CPEB2, LLPS propensity slightly but significantly declined towards younger clades (r = 0.67, n = 9, p < 0.05), and no significant change was observed for the PrD score, as exemplified in Figure 8 for the zebrafish and human orthologs. These findings indicate that the compositional changes observed in the four CPEB paralogs along the vertebrate lineage are associated to significant divergence in their overall sequence complexity and repetitiveness that directly affect the predicted propensity of the proteins to undergo LLPS and prion-like fibrillization. Thus, the CPEB evolutionary dynamics that we have uncovered may have critically contributed to the functional divergence of CPEB paralogs in the vertebrate lineage (see Discussion).

### The observed CPEB evolutionary trends are robust to random species sampling and intraclade variability

In the previous analyses, the number of CPEB orthologs that was analyzed per clade was determined by the availability of primary sequences in databases, which is not proportional to the actual clade size. To rule out that uneven species sampling may have contributed to the evolutionary trends that we observed, we repeated our analyses using for each clade a fixed number of randomly selected species (5). This randomized analysis was repeated for 10 times, using CPEB2 as a case study. Each time, we calculated the correlation coefficient between 24 parameters of interest (amino acid percent occurrences, as well as, SIM, REP, LLPS propensity, and prion-likeness scores) and clade stem ages. Remarkably, those correlations that were significant when using all the available sequences remained significant when using only 5 of them per clade, except for a single case, i.e., in 239 of 240 instances (Supplementary Table S3; Supplementary Figure S5). Thus, the evolutionary trends that we detected are largely independent of the degree of species sampling in clades.

Moreover, our previous analyses were performed using the mean values of the parameters of interest in each clade. As this approach did not consider the degree of intraclade variability of the parameters, we repeated our analysis for CPEB2 using the values of the parameters of interest for each individual species rather their mean values per clade. This analysis revealed that 13 out of 14 of the significant correlations that were detected for the parameters of interest remained significant even when not averaging values in each clade (Supplementary Table S4). The only exception was the percent occurrence of W residues, which are very rare in the protein, whose correlation coefficient fell slightly below significance. Thus, the observed evolutionary trends in CPEB composition- and function-related parameters remain significant even when considering their intraclade variability.

**FIGURE 9:**
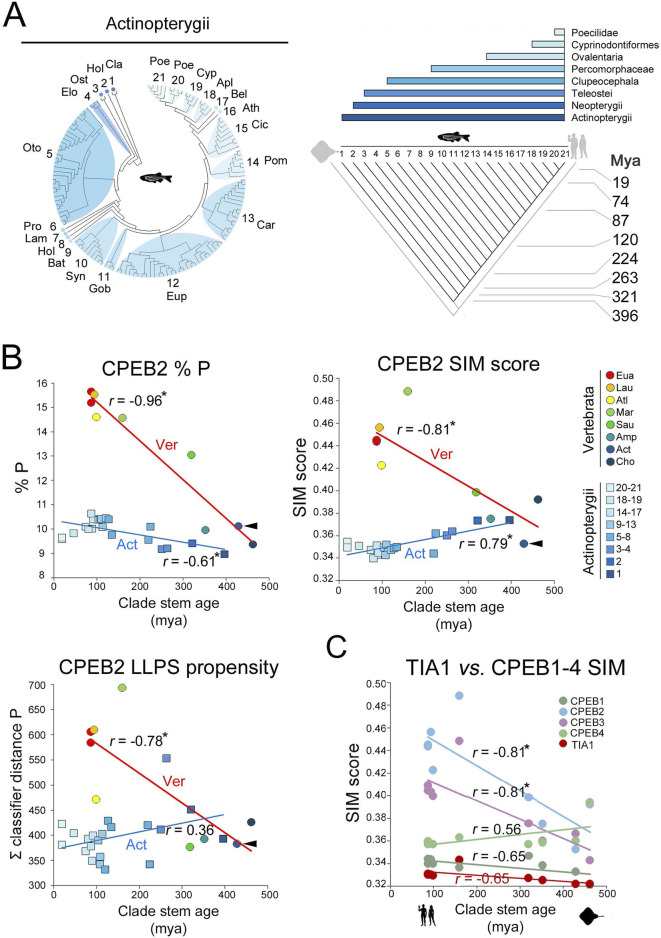
Intraclade versus interclade evolutionary variation in CPEB2 and evolutionary dynamics of TIA1 **(A)**. On the *left*, phylogenetic tree of the 134 species of the Actinopterygii clade with available CPEB2 sequences. Twenty-one subclades are numbered and labelled by a three-letter abbreviation of the clade name as listed in the *Methods* section. The cladogram on the right illustrates the phylogenetic relationships between the 21 clades shown in the phylogenetic tree. Clade stem ages are indicated. The *colored bars* on top indicate the higher-level clades (listed *on the right*) that variably comprise the 21 clades indicated in the cladogram. **(B)** Scatterplots with regression lines displaying, for CPEB2 orthologs, correlations across vertebrate clades (circles, *red regression line*) and Actinopterygii clades (squares, *blue regression line*) between the mean values of the indicated parameters (i.e., P occurrence in *upper left* panel, SIM score in *upper right* panel, ParSe LLPS propensity score in *lower left* panel) and clade stem ages. Data points are colored to indicate clades as reported in the legend *on the right*. Arrowheads indicate in each graph the datapoint relative to the mean value of the parameter in Actinopterygii. The *r* correlation coefficients are indicated for each regression line. *Asterisks* indicate statistically significant correlations. **(C)** As in Figure 7C, with datapoints and regression related to the TIA1 SIM score (in *red*) in comparison with the CPEB1-4 SIM scores.

To directly compare intraclade and interclade variability over a similar evolutionary time span in the vertebrate lineage (Figure 4), we repeated our analysis for the 24 parameters of interest within the Actinopterygii clade, using again CPEB2 as a case study. We performed this analysis in Actinopterygii as we had available CPEB2 sequences from a good number of species (Supplementary Table S1) from this clade that evolved over a long time period (396 my), comparable to that considered in our previous analysis across vertebrate clades (429 my). The mean values of the 24 parameters were analyzed in relation to the stem ages of 21 clades nested within Actinopterygii, ranging from Cladistia (396 mya) to Poeciliinae (18.9 mya; Figure 9A; see Methods). Although the number of available sequences for the oldest clades (from Cladistia to Elopocephala) was small, this analysis revealed that the variability of the 24 parameters within Actinopterygii is relatively limited in comparison with that across vertebrate clades over a similarly long evolutionary timespan (>400 million years; Figure 9B; Supplementary Figure S6). Within this clade, these relatively modest variation trends, some of which are statistically significant, go either in the same (e.g., % P, Figure 9B) or in the opposite (e.g., % LLPS propensity and prion-likeness, Figure 9B; Supplementary Figure S6) direction of those observed across vertebrate clades.

These findings indicate that the evolutionary dynamics of the CPEB composition- and function-related parameters that we observed across vertebrate clades appear to be lineage-specific. Thus, over a similar evolutionary timespan of ∼400 million years, their variation profiles radically differ, in magnitude and/or direction, along the vertebrate lineage or within its derived lineages, such as Actinopterygii. Therefore, the variation of some of the LCR-related parameters that we analyzed appears to mark evolutionary transitions across vertebrate clades, correlating with their stem ages. In this respect, the evolutionary dynamics of these parameters are comparable to those encountered for some other LCR-related parameters in the evolution of eukaryotic proteomes (Pelassa et al., 2019; see Discussion).

### The evolutionary changes observed in CPEBs are not generalized across prion-like RNA-binding proteins

The previous findings prompted us to test whether the evolutionary dynamics that we observed for CPEB2/3 are also detectable for other similar proteins, or whether they represent protein-/paralog-specific features.

Towards this aim, we studied the evolutionary history of TIA1, an LLPS-prone, prion-forming vertebrate protein containing multiple RRMs and a C-terminal LCR (Rayman and Kandel, 2017; Supplementary Figure S7A), i.e., a protein structurally and functionally related to CPEB2/3. We found that the TIA1 amino acid composition varied in a more limited manner compared to CPEB2/3 across vertebrate clades (Figure 9C; Supplementary Figures S7B–G). Notably, the TIA1 SIM and REP scores did not vary significantly (Supplementary Figures S7D–E). As for CPEB1, the protein has a relatively low LLPS propensity in older clades, which minimally, but significantly, increased going towards younger clades (r = −0.90, n = 9, p < 0.001; Supplementary Figure S7F). As for CPEB3, the prion-likeness of TIA1 is high and relatively stable throughout vertebrate phylogenesis, with only a taxon-specific drop in Actinopterygii, and a minimal increase from older to younger clades (r = −0.68, n = 9, p < 0.05; Supplementary Figure S7G). Thus, this protein has considerable prion-likeness and modest LLPS propensity already in older clades and maintained these features throughout the vertebrate phylogenetic tree, without substantial quantitative changes in sequence composition and complexity.

Thus, the evolutionary changes that we found for CPEB2/3 did not occur in similar proteins over the same evolutionary timespan, representing protein- and paralog-specific phenomena.

### Evolutionary divergence within the CPEB2-4 subfamily and the rise of CPEB2 as an LLPS-prone and prion-like protein in the vertebrate lineage

**FIGURE 10:**
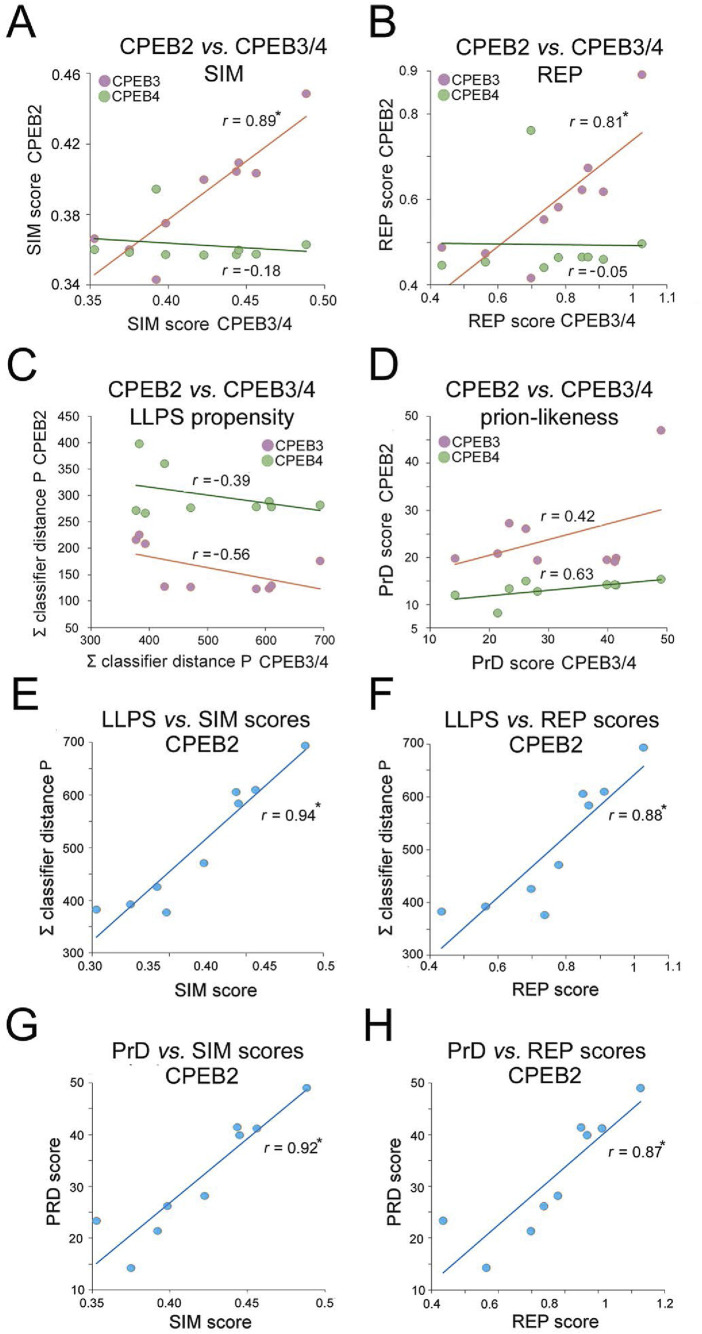
Divergent evolutionary variation of SIM, REP, LLPS, and prion-likeness scores across the CPEB2-4 subfamily orthologs. **(A)** Scatterplots with regression lines displaying correlations between the clade mean SIM scores of CPEB2 and those of CPEB3 (*purple*) or CPEB4 (*green*). The *r* correlation coefficients are indicated for each regression line. *Asterisks* indicate statistically significant correlations. **(B)** As in **(A)**, but for the REP score. Note how CPEB2 and CPEB3, but not CPEB4, underwent parallel evolutionary changes in both SIM and REP scores. **(C)** As in **(A)**, but for the LLPS propensity score (ParSe ∑ classifier distance P). **(D)** As in **(A)**, but for the prion-likeness score PLAAC (PrD score). Note how CPEB2 changes in LLPS propensity and prion-likeness scores do not correlate significantly with those of either CPEB3 or CPEB4. **(E)** Scatterplot with regression line displaying the significant correlation between the mean LLPS propensity and SIM scores of CPEB2 across vertebrate clades. The *r* correlation coefficients are indicated for each regression line. *Asterisks* (in this panel as well as in panels F-H) indicate statistically significant correlations. **(F)** As in **(E)**, for the correlation between the mean LLPS propensity and REP scores. **(G)** As in **(E)**, for the correlation between the mean PrD and SIM scores. **(H)** As in **(E)**, for the correlation between the mean PrD score and SIM scores. Note how the SIM and REP scores correlate indifferently with both LLPS propensity and prion-likeness scores.

Overall, the previous findings indicate that the complexity- and function-related parameters that were analyzed diverged significantly not only across members of the CPEB1 and CPEB2-4 subfamilies but also within the latter. Notably, going from older to younger clades, the SIM and REP scores significantly increase only for CPEB2/3, but not for CPEB4. Indeed, the SIM and REP scores of CPEB2 correlate significantly with those of CPEB3 (*r* = 0.89 and *r* = 0.81, respectively, n = 9 taxa, p < 0.01 in both instances), but not of CPEB4, across clades (*r* = 0.18, n = 9, p = 0.62, and *r* = 0.05, n = 9, p = 0.96, respectively; Figures 10A,B). However, CPEB2 and CPEB3 also diverged from each other in the impact of their sequence complexity changes on LLPS propensity and prion-likeness. Indeed, the LLPS propensity and prion-likeness scores of CPEB2 do not significantly correlate with those of CPEB3 (*r* = 0.39, n = 9 taxa, p = 0.29 and *r* = 0.42, n = 9 taxa, p = 0.26) and CPEB4 (*r* = 0.56, n = 9, p = 0.11 and *r* = 0.63, n = 9 taxa, p = 0.07) across clades (Figures 10C,D). Thus, two paralogs like CPEB2 and CPEB3 can display both parallel and divergent evolutionary trajectories with respect to different composition- and function-related parameters.

Overall, CPEB2 underwent the most conspicuous changes in amino acid composition and sequence complexity among the four CPEB paralogs along the vertebrate lineage. The considerable changes in SIM and REP scores correlated significantly with both LLPS propensity and prion-likeness scores (Figures 10E–H) at similarly high levels (r = 0.87-0.94, n = 9 and p < 0.01 in all instances), suggesting the absence of any obvious preferential link between the SIM and REP scores and either LLPS propensity or prion-likeness.

The results of these analyses reveal how CPEB2 arose as a second prion-like paralog of the CPEB family besides CPEB3 along the vertebrate lineage, reaching remarkable degrees of LLPS propensity and prion-likeness in the youngest vertebrate clades, including Glires and Primates.

## Discussion

We have systematically characterized the composition, complexity, LLPS propensity, and prion-likeness of the primary sequences of human CPEB1-4, studying their evolution in more than 500 species across nine major vertebrate clades. We found that the four CPEB paralogs underwent largely divergent evolutionary changes in composition and sequence complexity that varied their LLPS propensity and prion-likeness, with detectable trends going from older to younger vertebrate clades. These changes were particularly marked for CPEB2, which became a protein with high LLPS propensity and prion-likeness in younger clades, such as Glires and Primates. These findings expand our understanding of the molecular evolution of the CPEB protein family by defining, both qualitatively and quantitatively, how progressive changes in LCRs/AARs may have promoted the functional divergence of the four CPEB paralogs along the vertebrate lineage.

### Sequence composition and complexity in the human CPEB paralogs: structural and functional implications

We systematically analyzed the primary sequence features of the four human CPEB paralogs, focusing on their composition, sequence complexity, LLPS propensity, and prion-likeness. The combination of these sequence- and function-related quantitative parameters can provide a better understanding of how paralog-specific differences in LCR/AAR composition may determine functional differences across the four CPEB paralogous proteins. Our analyses substantially extend the breadth and scope of previous descriptive reports of the amino acid composition of some CPEB orthologs (e.g., Si et al., 2003b; Fiumara et al., 2010; Ramírez de Mingo et al., 2022).

In compositional terms, we found marked paralog-specific enrichments and depletions of several amino acids across the human CPEBs, which were associated with changes in the complexity and repetitiveness of their primary sequences, with CPEB2/3 displaying the lowest complexity and the highest repetitiveness. The biological meaning of these paralog-specific primary sequence differences is largely unknown and can only be interpreted based on our currently limited understanding of the structure and function of the CPEB NTRs.

Recent NMR structural analyses of human CPEB3 NTR fragments revealed a combination of random coil, α-helical, and polyproline-II (PP-II) conformations (Ramírez de Mingo et al., 2022). No NTR atomic-level structure is available for other paralogs. The AlphaFold models of human CPEBs show how the four NTRs are mostly disordered with interspersed structured segments, similar to what observed in the CPEB3 NMR structures. The enrichment in P/G structure-breaking residues, especially in CPEB2/3, may be key in maintaining the NTRs in a mostly disordered, flexible conformation. The disordered NTR portions can mediate LLPS through multivalent interactions (Gomes and Shorter, 2019). The interspersed secondary structure elements, which can be stabilized by folding-upon-binding mechanisms (Wright and Dyson, 2009), may cooperate with disordered regions in driving LLPS and fibrillization (Raveendra et al., 2013; Peskett et al., 2018; Vaglietti et al., 2023).

‘Molecular grammars’ are thought to exist by which the occurrence and order of amino acids in LCRs defines their LLPS behavior (Brangwynne et al., 2015; Martin and Mittag, 2018; Wang et al., 2018; Gomes and Shorter, 2019; Saar et al., 2021; Rekhi et al., 2024), although their fine ‘rules’ are not yet clearly understood. Martin and Mittag (2018) distinguished three types of LCRs, i.e. those enriched in either polar (and G), charged, or hydrophobic residues, with differential LLPS behaviors. The human CPEB LCRs, generally enriched in Q, S, G and depleted in charged and hydrophobic residues belong to the polar type, which is common in LLPS-prone proteins (Brangwynne et al., 2015). The LLPS of polar LCRs can be modulated by interspersed aromatic and charged residues (Martin and Mittag, 2018). Spaced aromatic (Y/F) and positively charged (R/K) residues govern the LLPS of certain proteins (Wang et al., 2018). While Y and K/R are underrepresented in CPEBs, F residues are scattered along their NTRs, as common in LLPS-prone proteins (Wang et al., 2018; Martin and Mittag, 2018). The variable enrichment in G, Q, and S residues may differentially shape the material properties of CPEB2-4 condensates (Wang et al., 2018). The prominent enrichment in P residues in the CPEB2-4 subfamily, may impact LLPS as P-rich regions play LLPS-modulating roles (Riback et al., 2017; Rekhi et al., 2024), also through proline cis-trans isomerization (Gomes and Shorter, 2019). Not all LLPS-driving LCRs are disordered and not all LLPS-driving domains are LCRs (Martin and Mittag, 2018; Dignon et al., 2020; Vaglietti et al., 2023), as also shown by our ParSe predictions. RRMs can contribute to LLPS in a complex interplay with LCRs (Wang et al., 2018) and future studies will have to finely dissect the interplay of NTRs and CTRs in CPEB LLPS.

Molecular grammars have also been proposed for functional prions (Alberti et al., 2009; Fiumara et al., 2010; Halfmann et al., 2011; Wake et al., 2024). Fiumara et al. (2010) classified these prions based on their composition, distinguishing Q/N-rich (type 1), Q/N/P/G-rich (type 3), and P/G-rich (type 5) prions, with intermediate degrees (types 2 and 4). The fibrillization of these prion classes can be triggered by distinct structural elements, i.e., α-helical coiled-coil (CCs) for Q/N-rich prions and β-sheets for prions richer in P/G residues, which may coexist (Fiumara et al., 2010; Hervas et al., 2021). Based on our analyses, human CPEB3 is a type 2 prion, bearing both type-1-like CC-prone regions (poly-Q, -A, and, -S AARs; Pelassa et al., 2014; Lilliu et al., 2018) and type-3 P/Q-rich regions, consistent with structural analyses of NTR fragments showing α-helical structures overlapping/flanking poly-Q, -A, and -S AARs, and PP-II and disordered conformations in P-rich regions (Ramírez de Mingo et al., 2022). P/G-rich patches may limit the fibrillization propensity of other NTR regions (Fiumara et al., 2010). Indeed, Reselammal et al. (2021) identified a core PrD subregion (a.a. 101-145) forming the rigid part of CPEB3 fibrils flanked by flexible proline-rich regions (a.a. 80-100 and 165-194; 37%–40% P). Thus, P residues in CPEB2/3 may profoundly shape their prion-like fibrillization besides their LLPS. CPEB2 is even more enriched in P/G than CPEB3, resembling a type 3 prion.

The prediction tools that we used correctly identified the protein regions known to drive the LLPS and prion-like fibrillization of human CPEBs based on experimental studies (Duran-Arqué et al., 2022; Stephan et al., 2015; Tsvetkov et al., 2020). On this basis, we further employed these tools in our evolutionary analyses (see below) to predict the contribution of specific NTR subregions to LLPS and prion-like fibrillization. ParSe identified specific LLPS-prone subregions that displayed an alternating pattern, with peripheral overlap, with the PrDs identified by PLAAC in CPEB2/3 (a.a. 210-250, 337-450, 489-566 in CPEB2; a.a. 1-35, 145-218 in CPEB3). These predictions suggest that different NTR subregions may be functionally specialized in driving either LLPS-condensation or prion-like fibrillization. Whether this is the case for CPEB2 remains to be experimentally determined. A few studies have attempted to initially identify functionally specialized subregions within the CPEB3 NTR, although with partially contradictory results. Stephan et al. (2015) mapped two PrDs in mouse CPEB3, i.e., PrD1 (a.a. 1-217; a.a. 1-216 in human CPEB3) and PrD2 (a.a. 284-449; a.a. 284-431 in human CPEB3), separated by an actin-binding region. The two PrDs predicted by PLAAC in our analyses overlap with PrD1. Reselammal et al. (2021) showed the key role of a PrD1 subregion (a.a. 101-194), containing one of the two PLAAC-predicted PrDs, in mouse CPEB3 fibrillization. Ramírez de Mingo et al. (2023) identified the a.a. 1-200 region of human CPEB3 as a PrD-like ‘amyloid-forming region’ and the distal NTR (a.a. 250-426), overlapping with PrD2 in Stephan et al. (2015), as the ‘phase-separation domain’. These partially contradictory findings, obtained in heterogeneous systems often only *in vitro*, together with our observations, indicate the need of careful molecular dissection approaches to identify NTR subregions mediating CPEB2/3 LLPS and/or fibrillization in the cellular context. Future studies will also have to better define the functional and temporal relationships between CPEB2/3 LLPS and prion-like fibrillization. For CPEB3, some studies view them as alternative states (Ford et al., 2019; 2023), while others see LLPS as an intermediate step towards fibrillization (Ramírez de Mingo et al., 2022; Ramírez de Mingo et al., 2023), as observed for other LCR-bearing proteins (Peskett et al., 2018; Vaglietti et al., 2023). While all these models have some experimental support, they remain largely speculative. Our findings can provide critical guidance in the further experimental dissection of the functional roles of NTR subregions in the LLPS and prion-like behavior of the vertebrate CPEB paralogs.

### Divergent LCR/AAR evolutionary variation as a driver of functional diversification in protein paralogs

The four paralogous CPEB genes appeared early in the vertebrate lineage, when many gene families diversified (Nishizawa and Nishizawa, 1999; Radó-Trilla et al., 2015). The appearance of LCRs/AARs and their evolutionary variation in length and composition, can contribute to the functional diversification of paralogous proteins with adaptive significance (e.g., Dover, 1989; Persi et al., 2016; 2023; Pelassa et al., 2019; Vaglietti et al., 2023). In our study, we initially characterized the marked differences in LCR-related sequence complexity, LLPS propensity, and prion-likeness across CPEB paralogs in *H. sapiens*, a species belonging to a relatively young terminal clade (Euarchontoglires) along the vertebrate lineage. In the second part of our study, we analyzed whether those differences are phylogenetically conserved–and were therefore present even in species from more ancient vertebrate clades–or whether they arose gradually, or at a specific points, along the vertebrate lineage. We found that composition- and function-related parameters of primary sequences varied in a largely divergent manner across CPEB paralogs through the vertebrate lineage, starting from Chondrichthyes. In general, for each paralog, these parameters were either relatively constant across clades or varied with trends largely related to clade stem ages along the lineage. Some clade-specific oscillations in their mean value, superimposed to clade stem age-related trends, were also found. We previously detected similar evolutionary trends for other LCR-related parameters at the level of entire proteomes (Pelassa et al., 2014; Pelassa et al., 2019). These trends were still detected when varying the sampling of species within clades and when accounting for the intraclade variability of the parameters, indicating that they are mostly related to inter- rather than intra-clade variation in the vertebrate lineage, at least for the CPEB case. Indeed, some complexity- and function-related parameters that varied markedly across vertebrate clades, correlating with clade stem ages, were instead relatively stable within clades, even with a long evolutionary history, such as Actinopterygii. Thus, changes in these parameters appear to be lineage-specific and to mark major evolutionary transitions across clades along the vertebrate lineage, as found for other LCR-related parameters in the evolution of eukaryotic proteomes (Pelassa et al., 2019).

In principle, either neutral evolution with genetic drift, selective forces, or their combination, may have shaped the evolutionary dynamics that we identified (e.g., Galtier, 2024).

The evolution of LCRs of variable complexity, from homopolymers (i.e., AARs), to oligopeptide repeats and regions of cryptic simplicity (Tautz et al., 1986; Enright et al., 2023), has been often modelled after that of selectively neutral microsatellites (Buschiazzo and Gemmell, 2006). AARs/LCRs originate from replication slippage and/or unequal crossing-over (Albà et al., 1999; Sainudiin et al., 2004; Owens et al., 2013; Warren et al., 1997). Synonymous or non-synonymous substitutions can lead, respectively, to their stabilization or interruption and loss (Buschiazzo and Gemmell, 2006;  Radó-Trilla and Albà, 2012; Lenz et al., 2014). LCRs can also arise from tandem duplications of gene segments (Nishizawa and Nishizawa, 1999) and GC-biased gene conversion (Galtier et al., 2009). These mechanisms, and thus AARs/LCRs occurrence and composition, can arise from clade-specific quantitative differences in slippage rates (Canceill et al., 1999; Flores and Engels, 1999; Ross et al., 2003; Laidlaw et al., 2007; Castillo-Lizardo et al., 2014), genome base composition (De Pristo et al., 2006; Tian et al., 2011), codon usage (Albà et al., 1999), unequal crossing-over (Hoffmann et al., 2008), and DNA repair mechanisms (Sia et al., 2001). At least some of these mechanisms may have contributed to the observed LCR/AAR variation across clades. Indeed, LCRs/AARs whose amino acids are encoded by GC-rich codons (e.g., A, G, P) are enriched in GC-rich mammalian genomes (Sumiyama et al., 1996; Nakachi et al., 1997), although this trend is not universal (Radó-Trilla and Albà, 2012). The evolutionary trends that we observed in CPEB P-rich/polyP, G-rich/polyG, and A-rich/polyA regions appear to be consistent with these trends.

However, a growing body of evidence is showing that selective forces also play substantial roles in the evolution of LCRs/AARs (Dover, 1989; Radó-Trilla and Albà, 2012; Radó-Trilla et al., 2015; Persi et al., 2016; Enright et al., 2023; Teekas et al., 2024) which are increasingly recognized as functional sequences rather than selectively neutral spacers (e.g., Dover, 1989; Fiumara et al., 2010; 2015; Pelassa et al., 2014; 2019; Pelassa and Fiumara, 2015; Chavali et al., 2017; Chavali et al., 2020; Marchetti et al., 2021; Vaglietti and Fiumara, 2021; Vaglietti et al., 2023). LCRs/AARs may be subject to selective pressure because variations in their length and composition alter protein structure (Fiumara et al., 2010) and interactions (Pelassa and Fiumara, 2015), also by convergent evolution with interactors (Vaglietti and Fiumara, 2021), as well as localization, through LLPS and aggregation, and physiological function (Vaglietti et al., 2023). Several lines of evidence, including analyses of mutation rates and codon usage (Hancock et al., 2001; Mularoni et al., 2010; Huntley and Golding, 2000; Haerty and Golding, 2010; Li et al., 2012), convergent evolution (Lavoie et al., 2003; Vaglietti et al., 2023), and sequence entropy (Enright et al., 2023), indicate that LCRs/AARs are subject to selective pressure. LCRs evolve more rapidly than other protein regions (Albà et al., 2007), with phases of relaxed purifying selection and positive selection followed by phases of intense purifying selection (Persi et al., 2016).

Two main lines of evidence in our findings suggest that selection played a role in shaping the evolution and divergence of LCR-related parameters in the vertebrate CPEB family. *First*, at least some of the LCR changes that we observed in the evolution of vertebrate CPEBs are predicted to directly impact their LLPS and prion-like behavior, and other aspects of their physiological activity, based not only on our *in silico* analyses but also on experimental evidence (e.g., Wang et al., 2018; Rekhi et al., 2024). These findings are consistent with the view that LCR variation is an evolutionary tool for regulating protein LLPS with adaptive effects (Martin and Mittag, 2018). For instance, changes in P-richness, such as those found in CPEB2/3, can regulate protein LLPS (Riback et al., 2017) and may have modulated CPEB3 interactions with actin mediated by its P-rich NTR (Radó-Trilla and Albà, 2012; Stephan et al., 2015). More in general, the evolutionary compositional changes in CPEBs may have contributed to shaping their interactomes (Pelassa and Fiumara, 2015; Mallik et al., 2022). Therefore, selection may have favored compositional changes in certain paralogs because of their direct impact on protein function and interactions. *Second*, both composition- and function-related parameters displayed clearly divergent evolutionary trends across paralogs, with some of them increasing or decreasing in certain paralogs, in a clade stem age-related manner, while remaining essentially stable in the other paralogs. These divergent evolutionary trajectories would be difficult to explain if the CPEB LCRs were selectively neutral. In the latter case, one may expect more similar trends across paralogs. Thus, it is plausible to speculate that, during vertebrate evolution, positive selection may have favored compositional changes in CPEB2/3 LCRs, while purifying selection may have maintained the composition of the CPEB1/4 LCRs relatively stable, consistent with observations of both adaptive and purifying selection acting at different stages of LCR evolution (Persi et al., 2016; 2023). From this perspective, our findings indicate that the CPEB2 LCRs may have undergone a phase of positive selection more recently than those of other paralogs.

In conclusion, we identified extensive patterns of LCR/AAR divergent evolution that may have had a key role in shaping the paralog-specific functions of CPEBs through the modulation of protein LLPS and prion-like behaviors. These findings identify the evolution of CPEBs as a paradigmatic example of the interplay of gene duplication and LCR variation in the functional diversification of protein families (Persi et al., 2023; Radó-Trilla et al., 2015; Chiu et al., 2022). Thus, they may provide key guidance for future experimental studies on the paralog-specific biological roles of the extensive LCRs of CPEBs, and their subregions, in LLPS and prion-like aggregation. Furthermore, they warrant further explorations of the LLPS and prion-like behaviors of CPEB2 in the context of vertebrate organisms, and their nervous systems, as previously done for CPEB3. Given the growing genetic, structural, and functional information on CPEB1-4, and the knowledge of their evolutionary dynamics that we traced here, the CPEB family represents an exquisite case study for investigating the impact of LCR evolution on the functional divergence of paralogous proteins.

## Materials and methods

### Protein primary sequences

The primary sequences of human CPEB1-4 were obtained from the NCBI protein database (https://www.ncbi.nlm.nih.gov/protein/; IDs: NP_001352171.1, NP_001170853.1, NP_055727.3, NP_085130.2, respectively), selecting isoforms reported as canonical in the Ensembl database. The reference human proteome was downloaded from the Uniprot database (https://www.uniprot.org/proteomes/UP000005640; Proteome ID: UP000005640, one sequence per gene, 20.590 proteins). The primary sequences of CPEB1-4 and TIA1 vertebrate orthologs (one per species) were downloaded in batch from the NCBI protein database. We selected sequences of orthologs of 571 species from nine major clades with different stem ages along the vertebrate lineage. The clades were defined based on a phylogenetic tree of the 571 species derived from TimeTree (Kumar et al., 2017; www.timetree.org), and on taxonomic information derived from NCBI Taxonomy (www.ncbi.nlm.nih.gov/taxonomy), using a branch-based clade definition approach. The stem age of each clade was derived from its divergence time from Euarchontoglires, or, for Euarchontoglires itself, from the divergence time between its constituent sister taxa, Glires and Euarchonta (obtained from the TimeTree database, median values; http://timetree.org/), as follows: Chondrichthyes (*Cho*, 12 species, 462 mya), Actinopterigyii (*Act*, 175 species, 429 mya), Amphibia/Lissamphibia (*Amp*, 11 species, 352 mya), Sauropsida (*Sau*, 159 species, 319 mya), Marsupialia (*Mar*, 8 species, 160 mya), Atlantogenata (*Atl*, 9 species, 99 mya), Laurasiatheria (*Lau*, 119 species, 94 mya), and two clades within Euarchontoglires, i.e., Glires (*Gli*, 45 species) and Primates (*Pri*, 34 species) which diverged 87 mya. Divergence times between clades were derived from TimeTree.org (median values). For each clade, the available sequences were aligned using MultAlin (Corpet, 1988) and the alignment was visually inspected. Sequences that appeared obviously incomplete in comparison with those of the same clade, i.e., lacking the initial methionine and/or with large deletions (>50 residues), were discarded and not further analyzed. After this selection process, most species (89%) still had 3-4 paralog sequences available for the analysis. The list of the selected sequences is reported in Supplementary Table S1. In some analyses (see Figure 9), the Actinopterigyii species with an available CPEB2 sequence were further divided into 21 clades based on their phylogenetic tree derived from TimeTree and taxonomic information derived from NCBI Taxonomy. For some of the clades (1-4, 6-9, 16-17) only a few (1-3) sequences were available. The stem age of each clade was derived from its divergence time from Poeciliinae, or for Poeciliinae itself, from the divergence time between of its two sister subclades to which *Poecilia* spp./*Poeciliopsis prolifica* and *Xiphophorus* spp./*Gambusia affinis* belong, as follows: *clade 1*, Cladistia (*Cla*, 2 species, 396 Mya); *clade 2*, Holostei (*Hol*, 1 species, 321 Mya); *clade 3*, Osteoglossocephala (*Ost*, 2 species, 263 Mya); *clade 4*, Elopocephala (*Elo*, 3 species, 250 Mya); *clade 5* (*Oto*, Otomorpha, 31 species, 224 Mya); *clade 6,* Protacanthopterygii (*Pro*, 1 species, 219 Mya); *clade 7,* Lampridacea (*Lam*, 1 species, 134 Mya); *clade 8*, Holocentrinomorphaceae (*Hol*, 1 species, 127 Mya); *clade 9*, Batrachoidaria (*Bat,* 1 species, 120 Mya); *clade 10*, Syngnathiaria/related Percomophaceae (*Syn*, 9 species, 109 Mya); *clade 11*, Gobiaria (*Gob*, 3 species, 108 Mya); *clade 12*, Eupercaria (*Eup*, 28 species, 113 Mya); *clade 13*, Carangaria/related Percomophaceae (*Car*, 16 species, 104 Mya); *clade 14*, Pomacentridae/Ovalentaria incertas sedis (*Pom*, 7 species, 87 Mya); *clade 15*, Cichlomorphae (*Cic*, 9 species, 91 Mya); *clade 16*, Atheriniformes (*Ath*, 1 species, 80 Mya); *clade 17*, Beloniformes, (*Bel*, 1 species, 89 Mya); *clade 18*, Aplocheiloidei (*Apl*, 4 species, 74 Mya); *clade 19*, Cyprinodontoidei (*Cyp*, 4 species, 46 Mya); *clade 20*, Poecilinae (*Poe*, *Poecilia* spp./*P. prolifica*; 4 species, 18.9 Mya); *clade 21*, Poeciliinae (*Poe*, *Xiphophorus* spp./*G. affinis*, 5 species, 18.9 Mya). Divergence times between clades were derived from TimeTree.org (median values).

### Compositional analyses of protein primary sequences

The percent occurrence of each amino acid, as well as the occurrence and length of AARs (≥4 residues) of the 20 amino acids, in protein primary sequences (of CPEB1-4, TIA1, or of the whole human proteome) were determined using previously developed Perl scripts (Pelassa et al., 2014; Marchetti et al., 2021). In case multiple repeats of one same amino acid were found in a protein, we calculated their total length as the sum of the individual repeat (≥4 residues) lengths and used this value for the evolutionary analyses. We used 20% and −20% over- or under-representation thresholds, respectively, to identify amino acids enriched or depleted in CPEB paralogs possibly in relation to the presence of compositionally biased regions (LCRs/AARs) in their primary sequences. These thresholds were empirically selected considering that in most proteins LCR/AARs regions constitute only a limited portion of the primary sequence. For instances, an average human protein of 500 residues is expected to contain ∼8%, i.e. 40, alanine (A) residues. If the initial 100 residues of the same protein were an alanine-rich region containing 16% A, the whole protein would then contain 48 (16 + 32) alanine residues, with a 20% increase in the percent occurrence of the amino acid, from 8% (40/500) to 9.6% (48/500). The same result would be obtained with an even shorter alanine-rich region (e.g., 50 residues) containing a higher percentage of alanine residues (e.g., 32%). Similarly, if the same protein contained a repeat of 10 alanine residues along its primary sequence, it would then contain 50 alanine residues (10%), i.e., ∼20% more than expected. Thus, deviations >20% in both directions in the occurrence of a given amino acid in the primary sequence of an average protein can signal the presence of compositionally biased regions of even modest length.

### Analyses of protein primary sequence complexity and repetitiveness

To define quantitatively the overall primary sequence complexity features of the proteins of interest, we calculated two per-residue sequence complexity-related scores, expressing the local degree of sequence simplicity (SIM) and repetitiveness (REP). The two scores were calculated, using *ad hoc* Perl scripts, in a sliding window of 20 residues centered around each residue (9 residues upstream, 10 residues downstream for all residues) along the protein primary sequence. For both scores, the sliding window length increased from 11 to 20 residues for the first 10 residues of the primary sequence and decreased from 20 to 10 residues for the last 10 residues.

In this 20-residue window, the SIM score was calculated as:

$$\text{SIM}=\frac{\text{CV}\left(a\right)}{\;1+\log\left(b\right)}$$

where CV is the coefficient of variation, *a* is a set of 20 values corresponding to the absolute number of occurrences of each amino acid in the 20-residue window (going from 0 to 20 for a given amino acid), and *b* is the number of amino acids occurring at least one time in the 20-residue window (going from 1 to 20). The “ACDEFGHIKLMNPQRSTVWY” sequence (in any order) has the minimum coefficient of variation of *a* (0) as well as the maximum *b* score (20), with the lowest possible SIM score (0). Any pure homopolymeric amino acid sequence, e.g., “AAAAAAAAAAAAAAAAAAAA”, has the maximum CV(*a*) (4.35) as well as the minimum *b* score (1), with the highest possible SIM score (4.35). The SIM score can thus vary from 0 to 4.35.

The REP score has been calculated as:

$$\text{REP}=\frac{a}{\sqrt{b}*\sqrt{c}}$$

Where *a* the total length of tandem repeats of at least two units of any amino acid (from 0 to 20), *b* is the number of tandem repeats of at least two units of any amino acid (from 1 for a 20-residues homopolymer to 10 in a sequence like “AACCDDEEFFGGHHIIKKLL”), and *c* is the number of different amino acids forming tandem repeats of at least two residues (from 0 to 10). The REP score can thus vary from 0 (for a sequence such as “ACDEFGHIKLMNPQRSTVWY”, in any order) to 20 (for any pure homopolymeric amino acid sequence, e.g., “AAAAAAAAAAAAAAAAAAAA”).

For each protein of interest. we calculated the mean per-residue SIM and REP scores across all the amino acids in the primary sequence. For the evolutionary analyses, we then calculated the mean values of these scores across all orthologs of a given protein in each clade of interest.

### Liquid-liquid phase separation (LLPS) propensity and prion-likeness predictions

The per-residue propensity to undergo LLPS for the primary sequence of the four human CPEB proteins was calculated using the FuzDrop algorithm, with a pDP score threshold of 0.6 to predict LLPS-prone protein regions (Vendruscolo and Fuxreiter, 2022) and the ParSe algorithm, version 2, taking into account three related phase-separation propensity metrics of the algorithm (Ibrahim et al., 2023), i.e., classifier distance, classifier distance with U_π_ + U_q_ extension (Δh°-trained), and classifier distance with U_π_ + U_q_ extension (c_sat_-trained), indicated as 1, 2, and 3, respectively, in Figures 2, 3. (https://stevewhitten.github.io/Parse_v2_FASTA; Ibrahim et al., 2023). ParSe is able to identify residues within LCR/IDR regions with (labelled as ‘P’) and without (labelled as ‘D’) propensity to undergo LLPS, as well as residues in folded regions (labelled as ‘F’) which are not predicted to have LLPS propensity. P-, D-, and F-labelled residues are depicted, respectively, in *red*, *gray*, and *turquoise* in the protein schemes shown in Figures 2, 3. To better highlight the local LLPS propensity of each CPEB region, we reported the category of each single residue (P, D, or F) in the plots, even though ParSe predicts as P, D, or F regions only if they are formed by at least 20 consecutive residues with the same label. ParSe was also used to obtain batch predictions of the LLPS propensity of the CPEBs vertebrate orthologs, using the ‘Σ classifier distance P’ score as a measure of the presence of LLPS-prone intrinsically disordered regions (IDRs) in the proteins of interest (Ibrahim et al., 2023). The prion-likeness of proteins was calculated using the PLAAC tool (Lancaster et al., 2014). To identify the position of potential PrDs in the four human paralogs (Figures 2–4), we plotted the per-residue PLAAC scores for the entire proteins, considering as PrDs continuous stretches of amino acids with PLAAC scores ≥0. To assess the overall prion-likeness of CPEB orthologs of a given clade in evolutionary analyses (Figures 6–10), we calculated their mean ‘PRDscore’ as provided by the PLAAC software.

### AlphaFold structural models

Atomic level structural models of CPEB1-4 paralogs of *H. sapiens* and *D. rerio,* as shown in Figures 1B, 8 were generated using the Colab AlphaFold2 software (Mirdita et al., 2022; available at https://colab.research.google.com/github/sokrypton/ColabFold/blob/main/AlphaFold2.ipynb). We selected the first one of the five structural models that were generated for each paralog. The models were downloaded as files in PDB format. The structures were visualized and pseudocolored (as in Figure 8) based on per-residue SIM, REP, LLPS propensity, or PrD scores using the UCSF Chimera software (Pettersen et al., 2004).

### Phylogenetic trees

Phylogenetic trees of the vertebrate and Actinopterigyii lineages were derived from TimeTree (timetree.org; Hedges et al., 2006) in Newick format and then processed using MEGA11 (Tamura et al., 2021) and RStudio software (Yu et al., 2017). Vertebrate species silhouettes (Public Domain Mark 1.0 and CC0 1.0 Universal Public Domain Dedication) were downloaded from PhyloPic (https://www.phylopic.org/). Credits: NASA (*H. sapiens*), Daniel Jaron (*Mus musculus*), Steven Traver (*Bos taurus, Loxodonta africana, Gallus gallus*), Daniel Stadtmauer (*Monodelphis domestica*), Andreas Hejnol (*Xenopus tropicalis*), Jake Warner (*D. rerio*), and Nathan Hermann (*Amblyraja radiata*).

### Evolutionary analyses of complexity- and function-related scores

We calculated 24 parameters of interest (i.e., the percent occurrences of the 20 amino acids and the mean per-residue SIM, REP, LLPS propensity, and prion-likeness scores) for each CPEB1-4 and TIA1 ortholog sequence. Then, we calculated the mean values of these 24 parameters across orthologs in each clade of interest (in vertebrates or Actinopterygii, see above). Finally, we calculated the Pearson’s *r* coefficients in correlations of the mean values of these parameters in each clade with clade stem ages. For CPEB2, we also performed the same analysis using the values of the 24 parameters of each individual ortholog protein (rather than their mean values across orthologs in each clade) or by considering only five randomly selected species per clade. The latter analysis was repeated 10 times with different sets of randomly selected orthologs per clade. The random selection of orthologs was performed using an *ad hoc* Perl script.

### Software and statistics

Available (Pelassa et al., 2014; 2019; Marchetti et al., 2021) and *ad hoc* software for bioinformatics analyses was written in Perl language (www.perl.org). Alignments of protein primary sequences used in the selection of ortholog sequences were obtained using Clustal Omega (Sievers et al., 2011) and Multalin (Corpet, 1988). Protein schemes were generated using Prosite MyDomains (Sigrist et al., 2012) using domain boundaries derived from Uniprot, NCBI protein, and SMART (http://smart.embl-heidelberg.de/; Letunic et al., 2021) databases and modified using Photoshop Elements 11 (Adobe), which was also used to generate figures. Plots of amino acid distributions along protein primary sequences were generated using the DrawProtein RStudio package (Brennan, 2018). Data analysis and statistics were performed using Statistica (TIBCO) and Excel (Microsoft), which was also used to generate graphs. The *r* correlation coefficient was calculated using Excel and its statistical significance assessed using the online Prism (GraphPad) calculator. A value of p ≤ 0.05 was considered as statistically significant in all instances.

## Data Availability

The raw data supporting the conclusions of this article will be made available by the authors, without undue reservation.
