# Supplementary material for: Divergent evolution of low-complexity regions in the vertebrate CPEB protein family
Source: Front Bioinform. 2025 Mar 20;5:1491735. doi: 10.3389/fbinf.2025.1491735 (PMC11965684; doi:10.3389/fbinf.2025.1491735)
Supplement: Supplementary file 1 [file Table4.pdf]

Supplemental Table 4 - *Pearson's r coefficients* for correlations between the indicated 24 parameters, calculated for each individual species or clade (mean value), and clade stem ages*

Paralogs	% A	% C	% D	% E	% F	% G	% H	% I	% K	% L	% M	% N	% P	% Q	% R	% S	%T	% V	% W	% Y	SIM	REP	LLPS	PRD
CPEB2-all species	**-0.74**	0.301	**0.854**	**0.923**	0.652	**-0.68**	0.281	**0.907**	**0.798**	**-0.79**	**0.771**	**0.82**	**-0.92**	-0.22	0.314	0.367	-0.21	**0.88**	0.565	**0.808**	**-0.91**	**-0.86**	**-0.81**	**-0.7**
CPEB-clade mean	-0.43	0.293	**0.929**	**0.947**	0.554	-0.4	0.344	**0.983**	0.634	**-0.85**	**0.868**	**0.8**	**-0.96**	-0.34	0.294	0.149	0.052	**0.957**	**0.785**	**0.815**	**-0.81**	**-0.77**	**-0.78**	**-0.77**

*the *r* value for statistically significant changes is in *green* (increase from older to younger clades) or in *red* (decrease from older to younger clades).

Legend

%X = mean percent amino acid frequency across ortholog primary sequences for each clade (where X is any amino acid)

SIM = SIM score

REP = REP score

LLPS = Σ classifier distance P (ParSE)

PRD = PRD score (PLAAC)
