# Supplementary material for: Divergent evolution of low-complexity regions in the vertebrate CPEB protein family
Source: Front Bioinform. 2025 Mar 20;5:1491735. doi: 10.3389/fbinf.2025.1491735 (PMC11965684; doi:10.3389/fbinf.2025.1491735)
Supplement: Supplementary file 2 [file Table1.pdf]

**Supplemental Table 1** - *NCBI sequence IDs of CPEB and TIA1 protein orthologs analyzed in this study*								
**Species #**	**Clade**	**Subclade (for Actinopterygii with available CPEB2 sequences)**	**Species names**	**CPEB1 IDs**	**CPEB2 IDs**	**CPEB3 IDs**	**CPEB4 IDs**	**TIA1 IDs**
1	PRI		Aotus nancymaae		XP_021523889.1	XP_012310193.1	XP_012316583.1	XP_012289357.1
2	PRI		Callithrix jacchus	XP_035159818.1	XP_008991833.3	XP_009008208.1	XP_002744608.1	XP_054100816.1
3	PRI		Carlito syrichta				XP_008061477.1	XP_008060561.1
4	PRI		Cebus imitator	XP_017357762.1	XP_017354194.1	XP_037583661.1	XP_017390588.1	
5	PRI		Cercocebus atys	XP_011947462.1	XP_011900836.1	XP_011918454.1	XP_011906434.1	XP_011895103.1
6	PRI		Chlorocebus sabaeus	XP_037843703.1	XP_037864621.1	XP_007961736.1	XP_008013514.1	XP_037862985.1
7	PRI		Colobus angolensis			XP_011803231.1	XP_011803201.1	XP_011800627.1
8	PRI		Gorilla gorilla	XP_055218788.1	XP_055241652.1	XP_018890579.1	XP_004043071.1	XP_030864091.1
9	PRI		Homo sapiens	NP_001352171.1	NP_001170853.1	NP_055727.3	NP_085130.2	NP_071505.2
10	PRI		Hylobates moloch	XP_032031450.1	XP_031991081.1	XP_031992641.1	XP_032014542.1	XP_058285044.1
11	PRI		Lemur catta		XP_045406348.1	XP_045423766.1	XP_045407101.1	
12	PRI		Macaca fascicularis	XP_015308684.1	XP_045248082.1	XP_045217127.1	XP_005558607.1	XP_045225070.1
13	PRI		Macaca mulatta	NP_001247755.1	XP_028704184.1	XP_015003173.2	XP_001098122.2	NP_001248687.1
14	PRI		Macaca nemestrina	XP_011766913.1	XP_011749891.1	XP_011737268.1	XP_011738398.1	XP_011711798.1
15	PRI		Macaca thibetana	XP_050652097.1	XP_050647601.1	XP_050660401.1	XP_050649148.1	XP_050610427.1
16	PRI		Mandrillus leucophaeus	XP_011856070.1			XP_011845573.1	XP_011840417.1
17	PRI		Microcebus murinus	XP_012618683.1	XP_012593026.1	XP_012621276.1	XP_012620967.1	
18	PRI		Nomascus leucogenys	XP_030670804.1	XP_030657040.1	XP_030665119.1	XP_030680816.1	XP_030683742.1
19	PRI		Nycticebus coucang		XP_053433601.1	XP_053438682.1	XP_053422576.1	XP_053443095.1
20	PRI		Otolemur garnettii			XP_012656849.2	XP_012664385.1	
21	PRI		Pan paniscus	XP_034795207.1	XP_034814198.1	XP_034786984.1	XP_003814055.1	XP_008954597.1
22	PRI		Pan troglodytes	XP_009427971.2	XP_016806846.2	XP_016774346.1	XP_001155432.1	XP_001141557.1
23	PRI		Papio anubis	XP_031524248.1	XP_009204806.3	XP_009213231.3	XP_003900584.1	XP_031511116.1
24	PRI		Piliocolobus tephrosceles	XP_023042953.1	XP_026311418.1	XP_023082011.1	XP_023074729.1	XP_023079704.1
25	PRI		Pongo abelii	XP_054387122.1	XP_054409687.1	XP_054378598.1	XP_054412230.1	XP_024097907.1
26	PRI		Pongo pygmaeus	XP_054306937.1	XP_054341927.1	XP_054292180.1	XP_054343837.1	XP_063504666.1
27	PRI		Propithecus coquereli			XP_012515172.1	XP_012505183.1	
28	PRI		Rhinopithecus bieti	XP_017750348.1		XP_017750195.1		XP_017709244.1
29	PRI		Rhinopithecus roxellana		XP_030779363.1	XP_030796373.1	XP_010371642.1	XP_010360194.1
30	PRI		Saimiri boliviensis		XP_039324465.1	XP_039321030.1	XP_003936184.1	XP_003922702.1
31	PRI		Sapajus apella	XP_032120695.1	XP_032128670.1	XP_032101425.1	XP_032116270.1	XP_032147716.1
32	PRI		Symphalangus syndactylus	XP_055133369.1	XP_055102321.1	XP_055111480.1	XP_055141188.1	XP_055097025.1
33	PRI		Theropithecus gelada		XP_025242284.1	XP_025253800.1	XP_025244393.1	XP_025210788.1
34	PRI		Trachypithecus francoisi	XP_033046841.1	XP_033069666.1	XP_033080234.1	XP_033036216.1	XP_033095226.1
35	GLI		Acomys russatus	XP_051004777.1	XP_051021037.1	XP_051002343.1	XP_051023338.1	XP_051010808.1
36	GLI		Apodemus sylvaticus	XP_052015645.1	XP_052055702.1	XP_052042616.1	XP_052052958.1	XP_052030171.1
37	GLI		Arvicanthis niloticus	XP_034362750.1	XP_034364437.1	XP_034378653.1	XP_034360789.1	XP_034367223.1
38	GLI		Arvicola amphibius	XP_038169856.1	XP_038176674.1	XP_038195952.1	XP_038184894.1	XP_038175042.1
39	GLI		Castor canadensis	XP_020039105.1			XP_020039331.1	XP_020038735.1
40	GLI		Cavia porcellus	XP_005006319.1			XP_013011637.1	XP_013007040.1
41	GLI		Chinchilla lanigera	XP_005381536.1		XP_013370305.1	XP_005376410.1	XP_005385675.1
42	GLI		Chionomys nivalis	XP_057611917.1	XP_057627358.1	XP_057633630.1	XP_057629585.1	XP_057619191.1
43	GLI		Cricetulus griseus	XP_027264086.1	XP_035308530.1	XP_027262128.1	XP_007639755.1	XP_007646760.1
44	GLI		Dipodomys spectabilis		XP_042530658.1	XP_042545183.1	XP_042528639.1	XP_042539011.1
45	GLI		Fukomys damarensis	XP_019062603.1			XP_010620458.1	XP_010607957.1
46	GLI		Grammomys surdaster		XP_028643646.1	XP_028629310.1	XP_028612268.1	XP_028618134.1
47	GLI		Heterocephalus glaber	XP_004852907.1		XP_012925306.1	XP_004836419.1	XP_004849968.1
48	GLI		Ictidomys tridecemlineatus	XP_040130628.1		XP_021587622.1	XP_005334165.1	XP_021580007.1
49	GLI		Jaculus jaculus	XP_004658273.2	XP_045017719.1	XP_045012953.1	XP_004665066.1	
50	GLI		Marmota flaviventris	XP_027811293.1	XP_034491815.1	XP_027795706.1	XP_027794393.1	XP_027790263.1
51	GLI		Marmota marmota	XP_048646141.1		XP_015355605.1	XP_048671230.1	XP_048660103.1
52	GLI		Marmota monax	XP_058433442.1	XP_046279607.1	XP_058437064.1	XP_046277336.1	
53	GLI		Mastomys coucha	XP_031243034.1	XP_031197972.1	XP_031246036.1	XP_031210440.1	XP_031238287.1
54	GLI		Meriones unguiculatus	XP_060223429.1	XP_021494396.2	XP_021486960.1	XP_060219871.1	XP_021490180.1
55	GLI		Mesocricetus auratus	XP_040585345.1	XP_021082088.2	XP_005063709.1	XP_005081596.1	XP_021083415.1
56	GLI		Microtus fortis	XP_050010081.1	XP_050005609.1	XP_050016712.1	XP_050019686.1	
57	GLI		Microtus ochrogaster	XP_026640090.1	XP_013208773.2	XP_005352240.1	XP_005349082.1	XP_013208469.1
58	GLI		Microtus oregoni	XP_041509930.1	XP_041492178.1	XP_041516971.1	XP_041531786.1	XP_041489455.1
59	GLI		Mus caroli		XP_029333985.1	XP_029328391.1	XP_029339653.1	XP_021020070.1
60	GLI		Mus musculus	NP_031781.1	NP_001355735.1	NP_001277756.1	NP_080528.2	NP_035715.1
61	GLI		Mus pahari		XP_029400799.1	XP_029392826.1	XP_021069207.1	XP_021047474.1
62	GLI		Myodes glareolus	XP_048290918.1			XP_048292008.1	XP_048272662.1
63	GLI		Nannospalax galili	XP_029421430.1		XP_008847828.1	XP_008849000.1	XP_029414595.1
64	GLI		Ochotona curzoniae	XP_040826081.1		XP_040826285.1	XP_040828319.1	XP_040849189.1
65	GLI		Ochotona princeps	XP_058521503.1		XP_058527329.1	XP_012783132.2	
66	GLI		Octodon degus	XP_004623387.1		XP_004632115.1	XP_004644884.1	XP_023574138.1
67	GLI		Onychomys torridus	XP_036042578.1	XP_036057584.1	XP_036023740.1	XP_036051669.1	XP_036037010.1
68	GLI		Oryctolagus cuniculus	XP_051690201.1	XP_051686044.1	XP_051679338.1	XP_002710434.1	
69	GLI		Perognathus longimembris			XP_048194654.1	XP_048190180.1	XP_048208638.1
70	GLI		Peromyscus californicus	XP_052615976.1	XP_052590801.1	XP_052568019.1	XP_052614866.1	XP_052578298.1
71	GLI		Peromyscus eremicus	XP_059128239.1	XP_059131801.1	XP_059114660.1	XP_059126234.1	XP_059114119.1
72	GLI		Peromyscus leucopus	XP_028729619.1	XP_028748767.1	XP_028738714.1	XP_037056455.1	XP_028709787.1
73	GLI		Peromyscus maniculatus			XP_006976707.2	XP_015853083.1	XP_015858558.1
74	GLI		Phodopus roborovskii	XP_051062597.1	XP_051056276.1	XP_051055538.1	XP_051036967.1	XP_051030984.1
75	GLI		Psammomys obesus			XP_055470069.1	XP_055460503.1	XP_055473634.1
76	GLI		Rattus norvegicus	NP_001099746.1	XP_038948724.1	NP_001406754.1	NP_001100462.1	
77	GLI		Rattus rattus	XP_032749212.1	XP_032772817.1	XP_032747745.1	XP_032768593.1	
78	GLI		Sciurus carolinensis	XP_047397061.1	XP_047423248.1	XP_047408221.1	XP_047411059.1	XP_047379089.1
79	GLI		Urocitellus parryii		XP_026258574.1	XP_026253139.1	XP_026270582.1	XP_026260899.1
80	LAU		Acinonyx jubatus	XP_053079466.1	XP_026893773.2	XP_014917412.3	XP_014928184.1	XP_014919480.1
81	LAU		Ailuropoda melanoleuca	XP_034523718.1			XP_002916973.1	
82	LAU		Artibeus jamaicensis	XP_037009507.1	XP_036982901.2	XP_037012483.1	XP_036983820.1	XP_053519326.1
83	LAU		Balaenoptera acutorostrata		XP_057402483.1	XP_057386610.1	XP_007190381.1	
84	LAU		Balaenoptera musculus		XP_036708183.1	XP_036685177.1	XP_036702996.1	XP_036728540.1
85	LAU		Balaenoptera ricei		XP_059779305.1	XP_059756356.1	XP_059773678.1	XP_059799034.1
86	LAU		Bison bison			XP_010840522.1	XP_010851822.1	XP_010843591.1
87	LAU		Bos indicus			XP_019808362.1	XP_019838361.1	XP_019826023.1
88	LAU		Bos javanicus	XP_061249997.1	XP_061276826.1	XP_061258780.1	XP_061249220.1	
89	LAU		Bos taurus	XP_024838094.1	XP_024849406.1	XP_015316086.2	XP_003587550.1	
90	LAU		Bubalus bubalis	XP_044789255.1	XP_025145467.1	XP_025129694.1	XP_006055450.1	XP_006079681.1
91	LAU		Bubalus kerabau	XP_055412601.1	XP_055445094.1	XP_055417049.1	XP_055410871.1	XP_055396536.1
92	LAU		Budorcas taxicolor	XP_052515702.1	XP_052497937.1	XP_052516868.1	XP_052515081.1	
93	LAU		Callorhinus ursinus	XP_025748983.1	XP_025743081.1	XP_025736592.1	XP_025722804.1	XP_025724246.1
94	LAU		Camelus bactrianus	XP_045372704.1			XP_010970574.1	XP_010962032.1
95	LAU		Camelus dromedarius	XP_031296492.1	XP_031293045.1	XP_031317244.1	XP_031293671.1	XP_010978175.1
96	LAU		Camelus ferus	XP_032324702.1	XP_032350099.1	XP_032347210.1	XP_006183495.1	XP_006192830.1

97	LAU		Canis lupus	XP_022272673.1	XP_038335802.1	XP_022267625.1		XP_025326578.1
98	LAU		Capra hircus	XP_017921850.1	XP_017905226.1	XP_017896949.1	XP_005694597.1	XP_017910954.1
99	LAU		Ceratotherium simum	XP_004432040.2	XP_004432409.1		XP_004428563.1	XP_004435634.1
100	LAU		Cervus canadensis	XP_043346303.1	XP_043303581.1	XP_043331939.1	XP_043344448.1	XP_043324893.1
101	LAU		Cervus elaphus	XP_043778150.1	XP_043762103.1	XP_043782563.1	XP_043743147.1	XP_043773455.1
102	LAU		Condylura cristata			XP_012579214.1	XP_004679289.1	XP_012587051.1
103	LAU		Dama dama	XP_061014778.1	XP_061001476.1	XP_061018161.1	XP_060985504.1	XP_061011099.1
104	LAU		Delphinapterus leucas		XP_030616046.1	XP_022425051.1	XP_022450794.1	XP_022416909.1
105	LAU		Delphinus delphis	XP_059859597.1	XP_059868777.1	XP_059890456.1	XP_059864351.1	XP_059882402.1
106	LAU		Desmodus rotundus	XP_024417489.1	XP_053778076.1	XP_053778431.1	XP_045041091.1	XP_045049737.1
107	LAU		Diceros bicornis		XP_058402728.1		XP_058402197.1	XP_058406992.1
108	LAU		Enhydra lutris		XP_022361646.1	XP_022355489.1	XP_022349289.1	XP_022359913.1
109	LAU		Eptesicus fuscus	XP_054569182.1	XP_054564074.1	XP_027988997.2	XP_028010371.1	XP_008145795.1
110	LAU		Equus asinus	XP_044616679.1	XP_044623749.1		XP_014688365.1	XP_014692180.1
111	LAU		Equus caballus	XP_023482643.1	XP_023494109.1		XP_001502854.1	XP_023474714.1
112	LAU		Equus przewalskii				XP_008523626.1	XP_008513060.1
113	LAU		Equus quagga	XP_046506514.1	XP_046511739.1		XP_046521962.1	XP_046519129.1
114	LAU		Erinaceus europaeus	XP_060035476.1	XP_007517932.2	XP_060047946.1	XP_007520885.1	
115	LAU		Eubalaena glacialis	XP_061037731.1	XP_061047230.1	XP_061027247.1	XP_061045921.1	XP_061024620.1
116	LAU		Eumetopias jubatus	XP_027945224.1	XP_027944677.1	XP_027954744.1	XP_027958517.1	XP_027972032.1
117	LAU		Felis catus	XP_011281076.1	XP_044912504.1	XP_023096492.1	XP_019694276.1	XP_011279409.1
118	LAU		Globicephala melas	XP_060149775.1	XP_030720090.2	XP_060142740.1	XP_030685759.1	XP_030733335.1
119	LAU		Halichoerus grypus		XP_035952439.1			XP_035943821.1
120	LAU		Hippopotamus amphibius		XP_057562084.1	XP_057592317.1	XP_057584821.1	XP_057597001.1
121	LAU		Hipposideros armiger	XP_019495780.1		XP_019494561.1	XP_019491499.1	XP_019503190.1
122	LAU		Hyaena hyaena		XP_039097498.1	XP_039086119.1		XP_039095472.1
123	LAU		Kogia breviceps		XP_058923207.1	XP_058911442.1	XP_058917677.1	XP_058935800.1
124	LAU		Lagenorhynchus albirostris	XP_060013738.1	XP_060004479.1	XP_059982183.1	XP_060000199.1	XP_060025023.1
125	LAU		Lagenorhynchus obliquidens		XP_026959134.1	XP_026967838.1	XP_026940066.1	XP_026987732.1
126	LAU		Leopardus geoffroyi	XP_045304584.1	XP_045330472.1	XP_045294155.1	XP_045356953.1	XP_045302322.1
127	LAU		Leptonychotes weddellii	XP_030892477.1			XP_030891484.1	XP_030892329.1
128	LAU		Lipotes vexillifer	XP_007470110.1		XP_007471617.1	XP_007465622.1	XP_007453468.1
129	LAU		Lontra canadensis	XP_032692723.1	XP_032710687.1	XP_032692306.1	XP_032707728.1	
130	LAU		Lutra lutra	XP_047593521.1	XP_047574860.1	XP_047557412.1	XP_047585497.1	
131	LAU		Lynx canadensis		XP_030170041.1	XP_030190416.1	XP_030183278.1	XP_030166640.1
132	LAU		Lynx rufus	XP_046942787.1	XP_046946548.1	XP_046929122.1	XP_046946939.1	XP_046923835.1
133	LAU		Manis javanica		XP_036846574.1	XP_036871655.1	XP_036850974.1	XP_017504365.1
134	LAU		Manis pentadactyla	XP_036787809.2	XP_036776988.2	XP_057362067.1	XP_036740738.1	XP_036764180.1
135	LAU		Meles meles	XP_045864803.1	XP_045850711.1	XP_045882478.1	XP_045855236.1	XP_045835036.1
136	LAU		Mesoplodon densirostris		XP_059959989.1	XP_059970294.1	XP_059950913.1	XP_059972880.1
137	LAU		Miniopterus natalensis				XP_016064270.1	
138	LAU		Mirounga angustirostris	XP_045755277.1	XP_045737420.1	XP_045734372.1	XP_045756921.1	XP_064428438.1
139	LAU		Mirounga leonina	XP_034880109.1	XP_034861176.1	XP_034880950.1	XP_034870555.1	XP_034857249.1
140	LAU		Molossus molossus	XP_036133721.1	XP_036117566.1	XP_036121274.1	XP_036097433.1	
141	LAU		Monodon monoceros	XP_029094046.1	XP_029090889.1	XP_029101024.1	XP_029075058.1	XP_029087635.1
142	LAU		Moschus berezovskii	XP_055273861.1		XP_055281058.1	XP_055277819.1	XP_055257220.1
143	LAU		Mustela erminea	XP_032197885.1	XP_032189755.1	XP_032169467.1	XP_032192885.1	XP_032208255.1
144	LAU		Mustela lutreola	XP_059035880.1	XP_059021193.1	XP_059025437.1	XP_059030314.1	
145	LAU		Mustela nigripes	XP_059227475.1	XP_059237115.1	XP_059254112.1	XP_059273363.1	XP_059261887.1
146	LAU		Mustela putorius	XP_004763730.1	XP_044931620.1	XP_004749718.1	XP_004737637.1	XP_012906590.1
147	LAU		Myotis brandtii				XP_014405817.1	XP_005867850.1
148	LAU		Myotis daubentonii	XP_059533892.1	XP_059531419.1	XP_059518816.1	XP_059555202.1	XP_059515498.1
149	LAU		Myotis davidii				XP_015420586.1	XP_015426978.1
150	LAU		Myotis lucifugus		XP_014317998.2	XP_023610604.1	XP_006097726.1	XP_006093019.1
151	LAU		Myotis myotis		XP_036184972.1	XP_036192493.1	XP_036169146.1	XP_036190106.1
152	LAU		Neofelis nebulosa	XP_058592433.1	XP_058579048.1	XP_058553136.1	XP_058579566.1	XP_058539777.1
153	LAU		Neogale vison	XP_044086487.1	XP_044082036.1	XP_044096033.1	XP_044085303.1	
154	LAU		Neomonachus schauinslandi		XP_021535108.2	XP_021555461.1	XP_044773043.1	XP_021554724.1
155	LAU		Neophocaena asiaeorientalis				XP_024609110.1	XP_024612133.1
156	LAU		Nyctereutes procyonoides	XP_055182534.1	XP_055182600.1	XP_055190372.1	XP_055181195.1	
157	LAU		Odobenus rosmarus	XP_004398319.2	XP_004410943.1	XP_012421557.1	XP_004392042.1	XP_004398702.1
158	LAU		Odocoileus virginianus	XP_020750836.1		XP_020725625.1	XP_020753554.1	
159	LAU		Orcinus orca		XP_033266564.2	XP_033260772.2	XP_004272062.1	XP_004277084.1
160	LAU		Oryx dammah	XP_040113450.1	XP_040093271.1	XP_040110453.1	XP_040118662.1	
161	LAU		Ovis aries	XP_042091051.1	XP_027826994.1	XP_027816172.1	XP_060256558.1	
162	LAU		Panthera leo	XP_042796825.1	XP_042792801.1	XP_042764989.1	XP_042802257.1	XP_042788513.1
163	LAU		Panthera onca	XP_060473533.1	XP_060511393.1	XP_060498009.1	XP_060491039.1	
164	LAU		Panthera pardus	XP_019315911.1	XP_019309231.2	XP_019300622.2	XP_019305891.1	XP_019296572.1
165	LAU		Panthera tigris	XP_042843905.1	XP_042840843.1	XP_042816932.1	XP_015393239.2	XP_015393532.1
166	LAU		Panthera uncia	XP_049470182.1	XP_049486279.1	XP_049502351.1	XP_049503727.1	XP_049507573.1
167	LAU		Phacochoerus africanus	XP_047652299.1	XP_047655328.1	XP_047615825.1	XP_047647302.1	XP_047637795.1
168	LAU		Phoca vitulina	XP_032247743.1	XP_032255848.1	XP_032288389.1	XP_032259627.1	XP_032266656.1
169	LAU		Phocoena sinus		XP_032488531.1	XP_032463891.1	XP_032481461.1	XP_032508015.1
170	LAU		Phyllostomus discolor	XP_035868235.1	XP_035874734.1	XP_028368100.1	XP_028383213.1	XP_028371839.1
171	LAU		Phyllostomus hastatus		XP_045689977.1	XP_045710158.1	XP_045675138.1	
172	LAU		Physeter catodon		XP_023975055.1	XP_028336899.1		
173	LAU		Pipistrellus kuhlii		XP_036276176.2	XP_036294444.1	XP_036280623.1	XP_045436827.1
174	LAU		Prionailurus bengalensis	XP_043410591.1	XP_043428876.1	XP_043452907.1	XP_043451625.1	XP_043459519.1
175	LAU		Prionailurus viverrinus	XP_047702699.1	XP_047711655.1	XP_047681924.1	XP_047709234.1	XP_047708505.1
176	LAU		Pteronotus parnellii		XP_054437919.1	XP_054428826.1	XP_054423593.1	
177	LAU		Pteropus alecto			XP_015455337.1	XP_024898866.1	XP_015443016.1
178	LAU		Pteropus giganteus	XP_039714072.1	XP_039694099.1	XP_039727664.1	XP_039734206.1	XP_039708368.1
179	LAU		Pteropus vampyrus			XP_023387210.1	XP_023379463.1	XP_011363869.1
180	LAU		Puma concolor				XP_025779400.1	XP_025788373.1
181	LAU		Puma yagouaroundi	XP_040349473.1		XP_040341785.1	XP_040301322.1	XP_040314544.1
182	LAU		Rhinolophus ferrumequinum	XP_032956173.1	XP_032961718.1	XP_032986725.1	XP_032952933.1	XP_032980477.1
183	LAU		Rousettus aegyptiacus	XP_015990894.1	XP_036083411.1	XP_036079413.1	XP_016000599.1	XP_016016863.1
184	LAU		Sorex araneus		XP_004619176.2	XP_054974849.1	XP_004611542.1	XP_004609199.1
185	LAU		Sorex fumeus		XP_055963277.1	XP_055986452.1	XP_055968347.1	XP_055983566.1
186	LAU		Sturnira hondurensis	XP_036909431.1	XP_036901555.1	XP_036903238.1	XP_036913691.1	XP_036904041.1
187	LAU		Suncus etruscus			XP_049646804.1	XP_049632004.1	XP_049641028.1
188	LAU		Suricata suricatta			XP_029788091.1	XP_029798694.1	XP_029793402.1
189	LAU		Sus scrofa	NP_001090979.1	XP_020955979.1	XP_020929151.1	XP_003134091.2	XP_020943058.1
190	LAU		Talpa occidentalis	XP_054550688.1		XP_037353483.1	XP_037356861.1	XP_037383456.1
191	LAU		Tursiops truncatus		XP_019784038.2	XP_019799613.2	XP_004315857.1	XP_004322043.1
192	LAU		Ursus americanus		XP_045627265.1	XP_045665904.1	XP_045648086.1	XP_045644153.1
193	LAU		Ursus arctos	XP_057159708.1	XP_026370204.2	XP_026337434.1	XP_026366779.1	XP_026339278.1
194	LAU		Ursus maritimus	XP_040498778.1	XP_040481003.1	XP_040480622.1	XP_040486547.1	XP_040479273.1
195	LAU		Vicugna pacos	XP_031547672.1			XP_006199030.1	XP_006201616.1
196	LAU		Vulpes lagopus	XP_041609608.1	XP_041607578.1	XP_041600143.1	XP_041606823.1	XP_041610943.1

197	LAU		Vulpes vulpes	XP_025856281.1		XP_025846351.1	XP_025862685.1	XP_025873640.1
198	LAU		Zalophus californianus	XP_027425414.1	XP_027454039.2	XP_027452437.1	XP_027460781.1	
199	ATL		Choloepus didactylus	XP_037689078.1	XP_037685337.1	XP_037661100.1	XP_037656727.1	XP_037662663.1
200	ATL		Chrysochloris asiatica	XP_006870997.1		XP_006831200.1	XP_006864859.1	XP_006862982.1
201	ATL		Dasypus novemcinctus	XP_058150718.1	XP_012374114.3	XP_058154752.1	XP_058138337.1	XP_004460179.1
202	ATL		Echinops telfairi	XP_045142333.1		XP_012859716.1	XP_004696852.1	XP_045150116.1
203	ATL		Elephantulus edwardii	XP_006885199.1		XP_006880396.1	XP_006884424.1	
204	ATL		Elephas maximus	XP_049762142.1	XP_049742442.1	XP_049711679.1	XP_049730297.1	XP_049713495.1
205	ATL		Loxodonta africana	XP_010592100.1		XP_023402691.1	XP_010590937.1	XP_003413712.1
206	ATL		Orycteropus afer	XP_007943310.1	XP_007948278.1	XP_007949452.1	XP_007949852.1	
207	ATL		Trichechus manatus	XP_004370721.1	XP_023598565.1	XP_012413395.2	XP_012415577.1	XP_004369293.1
208	MAR		Antechinus flavipes	XP_051837878.1		XP_051837596.1	XP_051834881.1	XP_051831428.1
209	MAR		Dromiciops gliroides	XP_043840497.1	XP_043826861.1	XP_043843576.1	XP_043845832.1	XP_043837728.1
210	MAR		Gracilinanus agilis	XP_044521716.1	XP_044537693.1	XP_044521610.1	XP_044520380.1	XP_044519508.1
211	MAR		Monodelphis domestica	XP_007478623.1	XP_016278799.2	XP_007478868.1	XP_001370640.1	XP_007476353.1
212	MAR		Phascolarctos cinereus	XP_020852705.1	XP_020848700.1	XP_020819849.1	XP_020824796.1	XP_020840987.1
213	MAR		Sarcophilus harrisii	XP_031812164.1		XP_031811903.1	XP_023362111.1	XP_031806497.1
214	MAR		Trichosurus vulpecula	XP_036625476.1	XP_036618558.1	XP_036593106.1	XP_036604627.1	XP_036604495.1
215	MAR		Vombatus ursinus	XP_027715017.1	XP_027692032.1	XP_027695681.1	XP_027697602.1	XP_027720926.1
216	SAU		Acanthisitta chloris	XP_009068221.1			XP_009077362.1	
217	SAU		Accipiter gentilis	XP_049668846.1	XP_049647207.1	XP_049666393.1	XP_049685210.1	XP_049686873.1
218	SAU		Agelaius phoeniceus		XP_054488768.1	XP_054493137.1	XP_054498777.1	XP_054506579.1
219	SAU		Ahaetulla prasina	XP_058012341.1		XP_058043106.1	XP_058021983.1	XP_058050868.1
220	SAU		Alligator mississippiensis	XP_014459976.1	XP_059577568.1	XP_019339769.1	XP_019333943.1	XP_006272065.1
221	SAU		Alligator sinensis	XP_025047829.1		XP_025066672.1	XP_006028788.1	
222	SAU		Ammospiza caudacuta	XP_058667332.1	XP_058659498.1	XP_058666397.1	XP_058671480.1	XP_058676136.1
223	SAU		Ammospiza nelsoni	XP_059338487.1	XP_059325839.1	XP_059332647.1	XP_059339416.1	XP_059346900.1
224	SAU		Anas platyrhynchos	XP_038041103.1	XP_038034288.1	XP_021123893.3	XP_027324525.1	XP_038023052.1
225	SAU		Anolis carolinensis		XP_008119130.1	XP_016851398.1	XP_008102995.1	XP_062817068.1
226	SAU		Anolis sagrei		XP_060633943.1	XP_060624680.1	XP_060621102.1	XP_060643146.1
227	SAU		Anser cygnoides	XP_047937475.1	XP_047901970.1	XP_047925172.1	XP_013047291.1	XP_047901697.1
228	SAU		Antrostomus carolinensis				XP_028939958.1	
229	SAU		Apaloderma vittatum	XP_009862988.1				
230	SAU		Aptenodytes forsteri	XP_009274599.1		XP_009276050.2	XP_009284024.1	
231	SAU		Apteryx mantelli			XP_013806603.1		XP_067168639.1
232	SAU		Apteryx rowi	XP_025924070.1	XP_025946420.1	XP_025917690.1	XP_025918872.1	XP_025937516.1
233	SAU		Apus apus	XP_051484716.1	XP_051473787.1	XP_051475375.1	XP_051486897.1	
234	SAU		Aquila chrysaetos	XP_029870932.1	XP_029869422.1	XP_029887395.1	XP_029853498.1	XP_029890730.1
235	SAU		Athene cunicularia	XP_026712188.1		XP_026707007.1	XP_026713721.1	
236	SAU		Aythya fuligula	XP_032051041.1		XP_032047646.1	XP_032052531.1	XP_032059946.1
237	SAU		Balearica regulorum	XP_010300237.1				
238	SAU		Buceros rhinoceros	XP_010141474.1			XP_010130324.1	
239	SAU		Calidris pugnax	XP_014817105.1	XP_014792524.1	XP_014821190.1	XP_014803461.1	
240	SAU		Calypte anna		XP_030305353.1	XP_030308981.1	XP_030315312.1	XP_030320016.1
241	SAU		Camarhynchus parvulus	XP_030811123.1	XP_030802674.1	XP_030806606.1	XP_030813121.1	
242	SAU		Caretta caretta	XP_048723392.1	XP_048704756.1	XP_048711952.1	XP_048717163.1	XP_048685011.1
243	SAU		Cariama cristata	XP_009702269.1			XP_009694976.1	
244	SAU		Catharus ustulatus	XP_032926622.1	XP_032917221.1	XP_032921528.1	XP_032928705.1	XP_032937184.1
245	SAU		Centrocercus urophasianus	XP_042680590.1	XP_042668431.1	XP_042673856.1	XP_042682001.1	XP_042695482.1
246	SAU		Chaetura pelagica	XP_010004215.1		XP_009998348.1	XP_010000274.1	
247	SAU		Charadrius vociferus	XP_009893849.1		XP_009886987.1	XP_009880374.1	
248	SAU		Chelonia mydas	XP_043379921.1	XP_037753966.1	XP_027675219.2	XP_037762276.1	XP_037742447.1
249	SAU		Chelonoidis abingdonii	XP_032647041.1		XP_032626155.1	XP_032657383.1	XP_032620729.1
250	SAU		Chiroxiphia lanceolata	XP_032556037.1	XP_032541268.1	XP_032551079.1	XP_032558463.1	XP_032568363.1
251	SAU		Chlamydotis macqueenii	XP_010114813.1				
252	SAU		Chrysemys picta	XP_008164995.2	XP_042711387.1	XP_042712732.1	XP_005298123.1	XP_005285275.1
253	SAU		Colius striatus	XP_010200813.1		XP_010193732.1		
254	SAU		Columba livia	XP_005515554.1		XP_021149440.1	XP_021144600.1	XP_064897128.1
255	SAU		Corapipo altera	XP_027513510.1	XP_027498509.1	XP_027504110.1	XP_027498939.1	XP_027525560.1
256	SAU		Corvus brachyrhynchos	XP_008629484.1		XP_017581351.1	XP_017583692.1	
257	SAU		Corvus cornix	XP_039413603.1	XP_039407259.1	XP_039410330.1	XP_010397945.1	XP_039420382.1
258	SAU		Corvus hawaiiensis	XP_048173565.1	XP_048160785.1	XP_048166661.1	XP_048176282.1	XP_048146171.1
259	SAU		Corvus kubaryi	XP_041878695.1		XP_041874949.1		XP_041875994.1
260	SAU		Corvus moneduloides	XP_031978699.1	XP_031966222.1	XP_031972967.1	XP_031981202.1	XP_031948112.1
261	SAU		Coturnix japonica	XP_015728221.1	XP_015718247.1	XP_015722422.1	XP_015731680.1	XP_015738437.1
262	SAU		Crocodylus porosus	XP_019388794.1		XP_019411638.1	XP_019390509.1	XP_019401513.1
263	SAU		Crotalus tigris	XP_039195480.1			XP_039207967.1	XP_039180089.1
264	SAU		Cuculus canorus	XP_053933791.1	XP_053920273.1	XP_053927229.1	XP_009566333.1	XP_053944870.1
265	SAU		Cyanistes caeruleus	XP_023790109.1		XP_023785428.1	XP_023791573.1	
266	SAU		Cygnus atratus	XP_035422666.1	XP_035403087.1	XP_050568007.1	XP_035415628.1	XP_035418669.1
267	SAU		Cygnus olor	XP_040425991.1	XP_040410941.1	XP_040419748.1	XP_040429102.1	XP_040394007.1
268	SAU		Dermochelys coriacea	XP_038275100.1	XP_043370071.1	XP_038266851.1	XP_038268719.1	
269	SAU		Dromaius novaehollandiae	XP_025972279.1	XP_025977714.1	XP_025949911.1	XP_025978394.1	XP_064353869.1
270	SAU		Dryobates pubescens	XP_009896738.2	XP_054028119.1	XP_054019599.1	XP_009902939.1	XP_054031234.1
271	SAU		Egretta garzetta	XP_009642185.1		XP_035753219.1	XP_035758405.1	
272	SAU		Empidonax traillii	XP_027745205.1		XP_027764905.1	XP_027757018.1	
273	SAU		Eublepharis macularius		XP_054855093.1	XP_054839188.1	XP_054831191.1	XP_054853116.1
274	SAU		Euleptes europaea			XP_056706070.1	XP_056710639.1	XP_056716933.1
275	SAU		Eurypyga helias	XP_010160273.1				
276	SAU		Falco biarmicus	XP_056203592.1	XP_056190964.1	XP_056207152.1	XP_056204089.1	XP_056219007.1
277	SAU		Falco cherrug	XP_055573176.1	XP_055557253.1	XP_014134658.1	XP_027661410.1	XP_055552146.1
278	SAU		Falco naumanni	XP_040458164.1	XP_040450224.1	XP_040462988.1	XP_040461100.1	XP_040473920.1
279	SAU		Falco peregrinus	XP_055649634.1	XP_055650961.1	XP_027644696.2	XP_027634590.1	XP_055676253.1
280	SAU		Falco rusticolus	XP_037251866.1	XP_037240435.1	XP_037256385.1	XP_037254801.1	XP_037227457.1
281	SAU		Ficedula albicollis	XP_005051700.2	XP_005045655.2	XP_005048482.1	XP_005053691.1	
282	SAU		Fulmarus glacialis	XP_009574772.1				XP_009584902.1
283	SAU		Gallus gallus	XP_025009583.1	XP_015141289.2	XP_015144323.1	XP_414678.3	
284	SAU		Gavia stellata	XP_059679859.1	XP_059673385.1	XP_059677225.1	XP_009811721.1	XP_059687342.1
285	SAU		Gavialis gangeticus	XP_019360069.1		XP_019358185.1	XP_019361943.1	XP_019369902.1
286	SAU		Gekko japonicus				XP_015265057.1	XP_015276622.1
287	SAU		Geospiza fortis	XP_030916875.1		XP_030917803.1	XP_005418859.1	
288	SAU		Gopherus evgoodei		XP_030420278.1	XP_030427152.1	XP_030429373.1	XP_030407806.1
289	SAU		Gopherus flavomarginatus	XP_050824835.1	XP_050803502.1	XP_050815385.1	XP_050818605.1	XP_050796126.1
290	SAU		Grus americana	XP_054693458.1	XP_054679782.1	XP_054688341.1	XP_054697349.1	XP_054660550.1
291	SAU		Gymnogyps californianus	XP_050759042.1	XP_050752156.1	XP_050755173.1	XP_050761533.1	XP_050766145.1
292	SAU		Haemorhous mexicanus	XP_059714138.1	XP_059701128.1	XP_059707639.1	XP_059716065.1	XP_059726626.1
293	SAU		Haliaeetus albicilla	XP_009927804.1		XP_009928450.1		XP_069657195.1
294	SAU		Haliaeetus leucocephalus	XP_010569858.1	XP_010575185.1	XP_010563215.1	XP_010577362.1	
295	SAU		Harpia harpyja	XP_052664504.1	XP_052636414.1	XP_052654912.1	XP_052628185.1	XP_052661960.1
296	SAU		Hemicordylus capensis	XP_053128724.1	XP_053109175.1	XP_053167526.1	XP_053151302.1	XP_053125095.1

297	SAU		Heteronotia binoei	XP_060116266.1	XP_060102163.1	XP_060097331.1	XP_060096550.1	XP_060107572.1
298	SAU		Hirundo rustica	XP_039933502.1		XP_039926757.1	XP_039933793.1	XP_039943519.1
299	SAU		Indicator indicator	XP_054243795.1	XP_054247497.1	XP_054238149.1	XP_054244887.1	
300	SAU		Lacerta agilis	XP_033023138.1	XP_033016959.1	XP_033005179.1	XP_032995858.1	XP_033015019.1
301	SAU		Lagopus leucura	XP_042741740.1	XP_042727375.1	XP_042718962.1	XP_042736704.1	XP_042746844.1
302	SAU		Lagopus muta	XP_048812308.1	XP_048796894.1	XP_048801244.1	XP_048816145.1	XP_048784098.1
303	SAU		Lepidothrix coronata	XP_017672524.1	XP_017680020.1	XP_017659345.1	XP_017678394.1	XP_017687429.1
304	SAU		Leptosomus discolor	XP_009944685.1				
305	SAU		Lonchura striata		XP_021410086.1	XP_021392141.1	XP_021387807.1	XP_021401072.1
306	SAU		Malaclemys terrapin	XP_053898359.1	XP_053885444.1	XP_053890782.1	XP_053894253.1	XP_053873460.1
307	SAU		Malurus melanocephalus		XP_057230427.1	XP_057237110.1	XP_057239482.1	
308	SAU		Manacus candei	XP_051652410.1	XP_051654390.1	XP_051656524.1	XP_051649522.1	XP_051668059.1
309	SAU		Manacus vitellinus	XP_017925390.1		XP_029814473.1	XP_017929079.2	XP_029820865.1
310	SAU		Mauremys mutica	XP_044836773.1	XP_044874332.1	XP_044878991.1		XP_044860409.1
311	SAU		Mauremys reevesii	XP_039348789.1	XP_039395982.1	XP_039402317.1	XP_039340294.1	XP_039380724.1
312	SAU		Meleagris gallopavo	XP_019474855.1		XP_010712865.1	XP_010717265.1	
313	SAU		Melopsittacus undulatus	XP_005142554.1	XP_033920809.1	XP_030900553.2	XP_012982816.1	XP_033926311.1
314	SAU		Melospiza georgiana		XP_057880245.1	XP_057884612.1	XP_057891038.1	XP_057898358.1
315	SAU		Melozone crissalis		XP_054149491.1	XP_054132582.1	XP_054143914.1	XP_054151092.1
316	SAU		Mesitornis unicolor	XP_010186684.1		XP_010191140.1	XP_010180673.1	
317	SAU		Molothrus ater		XP_036237612.1	XP_036241139.1	XP_036247678.1	XP_036252719.1
318	SAU		Motacilla alba	XP_038002930.1	XP_037990781.1	XP_037996188.1	XP_038005755.1	
319	SAU		Myiozetetes cayanensis	XP_050165616.1	XP_050194080.1	XP_050161210.1	XP_050168458.1	XP_050181704.1
320	SAU		Neopelma chrysocephalum	XP_027553872.1	XP_027542187.1	XP_027528194.1	XP_027538780.1	XP_027562073.1
321	SAU		Neopsephotus bourkii	XP_061205870.1	XP_061235772.1	XP_061204987.1	XP_061216273.1	XP_061204205.1
322	SAU		Nestor notabilis			XP_010019708.1	XP_010014215.1	
323	SAU		Nipponia nippon	XP_009467893.1		XP_009463625.1	XP_009470448.1	
324	SAU		Notechis scutatus			XP_026526520.1	XP_026543881.1	XP_026540525.1
325	SAU		Nothoprocta perdicaria	XP_025899713.1	XP_025908154.1	XP_025907108.1	XP_025903454.1	XP_025898707.1
326	SAU		Numida meleagris	XP_021262385.1	XP_021249627.1	XP_021254936.1	XP_021266625.1	XP_021230555.1
327	SAU		Oenanthe melanoleuca	XP_056355373.1	XP_056346031.1	XP_056351151.1	XP_056357987.1	XP_056364827.1
328	SAU		Onychostruthus taczanowskii	XP_041274612.1	XP_041258820.1	XP_041271779.1	XP_041253691.1	XP_041260572.1
329	SAU		Opisthocomus hoazin			XP_009933978.1	XP_009929424.1	
330	SAU		Oxyura jamaicensis	XP_035191669.1	XP_035180178.1	XP_035185418.1	XP_035194682.1	XP_035200904.1
331	SAU		Pantherophis guttatus	XP_034288525.1		XP_034286335.1	XP_034261248.1	XP_034286552.1
332	SAU		Parus major	XP_015494635.1	XP_015480428.1	XP_015488048.1	XP_015497194.1	
333	SAU		Passer montanus	XP_039578735.1	XP_039554664.1	XP_039564636.1	XP_039557664.1	
334	SAU		Pelecanus crispus	XP_009489632.1		XP_009490412.1		
335	SAU		Pelodiscus sinensis			XP_025035569.1	XP_006139234.1	
336	SAU		Pezoporus flaviventris	XP_061303368.1	XP_061325353.1	XP_061315119.1	XP_061312660.1	XP_061334989.1
337	SAU		Pezoporus wallicus	XP_057276554.1	XP_057282834.1	XP_057258278.1	XP_057267981.1	XP_057258533.1
338	SAU		Phaethon lepturus	XP_010282948.1		XP_010286856.1		
339	SAU		Phalacrocorax carbo	XP_009511544.1				XP_064328722.1
340	SAU		Phasianus colchicus	XP_031472069.1	XP_031444251.1	XP_031453582.1	XP_031453968.1	XP_031452854.1
341	SAU		Pipra filicauda			XP_039235118.1	XP_027574280.1	XP_027602504.1
342	SAU		Podarcis muralis	XP_028562303.1	XP_028598991.1	XP_028585633.1	XP_028573657.1	XP_028597015.1
343	SAU		Podarcis raffonei	XP_053220917.1	XP_053260481.1	XP_053244725.1	XP_053232599.1	XP_053257403.1
344	SAU		Poecile atricapillus	XP_058703268.1	XP_058694039.1	XP_058697135.1	XP_058705050.1	XP_058715481.1
345	SAU		Pogona vitticeps	XP_020659469.1		XP_020638577.1	XP_020662374.1	XP_020635803.1
346	SAU		Protobothrops mucrosquamatus	XP_015673943.1			XP_015674111.1	XP_015666618.1
347	SAU		Pseudonaja textilis			XP_026566338.1	XP_026552177.1	XP_026570905.1
348	SAU		Pseudopodoces humilis			XP_014105863.1	XP_014108580.1	XP_005532308.1
349	SAU		Pterocles gutturalis	XP_010075543.1			XP_010083296.1	
350	SAU		Pygoscelis adeliae	XP_009322134.1		XP_009321539.1	XP_009316748.1	
351	SAU		Pyrgilauda ruficollis		XP_041331722.1	XP_041317355.1	XP_041345918.1	XP_041321863.1
352	SAU		Python bivittatus	XP_007428520.1		XP_007429206.1	XP_007424411.1	
353	SAU		Rhineura floridana	XP_061451440.1	XP_061439808.1	XP_061491530.1	XP_061472905.1	XP_061448739.1
354	SAU		Rissa tridactyla	XP_054070368.1	XP_054058742.1	XP_054063187.1	XP_054072895.1	XP_054080940.1
355	SAU		Sceloporus undulatus	XP_042332562.1	XP_042326512.1	XP_042315204.1	XP_042310091.1	XP_042328019.1
356	SAU		Serinus canaria	XP_018769243.1	XP_030094637.2	XP_050831964.1	XP_009097550.1	XP_050838997.1
357	SAU		Sphaerodactylus townsendi			XP_048362614.1	XP_048344113.1	XP_048369093.1
358	SAU		Strigops habroptila	XP_030352794.1	XP_030347621.1	XP_030341716.1	XP_030360251.1	XP_030365718.1
359	SAU		Struthio camelus	XP_009665281.1		XP_009676071.1	XP_009665176.1	XP_068776760.1
360	SAU		Sturnus vulgaris	XP_014745520.1	XP_014738670.1	XP_014730816.1	XP_014731418.1	XP_014740647.1
361	SAU		Taeniopygia guttata	XP_030137661.3	XP_030126622.1	XP_012430079.3	XP_002196316.2	XP_032608293.1
362	SAU		Tauraco erythrolophus	XP_009976443.1				
363	SAU		Terrapene carolina		XP_026503822.1		XP_024055116.1	
364	SAU		Thamnophis elegans	XP_032089583.1		XP_032087958.1	XP_032065385.1	XP_032085416.1
365	SAU		Thamnophis sirtalis			XP_013909709.1	XP_013909257.1	
366	SAU		Tinamus guttatus	XP_010225926.1				
367	SAU		Trachemys scripta	XP_034639741.1	XP_034626561.1	XP_034632532.1	XP_034635596.1	XP_034617645.1
368	SAU		Tympanuchus pallidicinctus	XP_052528064.1	XP_052539282.1	XP_052533863.1	XP_052541027.1	XP_052558277.1
369	SAU		Tyto alba	XP_032846368.1	XP_032840331.2	XP_032849936.1	XP_009974566.1	
370	SAU		Varanus komodoensis		XP_044274288.1	XP_044276620.1	XP_044310307.1	XP_044273265.1
371	SAU		Vidua chalybeata	XP_053810362.1	XP_053797951.1	XP_053804750.1	XP_053812207.1	XP_053822638.1
372	SAU		Vidua macroura	XP_053843997.1	XP_053830842.1	XP_053839248.1	XP_053846716.1	XP_053856562.1
373	SAU		Zonotrichia albicollis	XP_005490360.1		XP_026655503.1	XP_005492948.1	
374	SAU		Zootoca vivipara	XP_034988009.2	XP_060135057.1		XP_034961201.1	XP_034974122.1
375	AMP		Bombina bombina		XP_053560104.1	XP_053548387.1	XP_053573116.1	XP_053574133.1
376	AMP		Bufo bufo	XP_040269712.1	XP_040275022.1	XP_040291879.1	XP_040262242.1	XP_040270362.1
377	AMP		Bufo gargarizans	XP_044139276.1	XP_044152640.1	XP_044153266.1	XP_044136671.1	
378	AMP		Geotrypetes seraphini	XP_033776035.1	XP_033804832.1	XP_033799081.1	XP_033782998.1	XP_033804744.1
379	AMP		Hyla sarda	XP_056428596.1	XP_056411143.1	XP_056385362.1	XP_056430827.1	XP_056427437.1
380	AMP		Microcaecilia unicolor	XP_030043833.1	XP_030047055.1	XP_030058862.1	XP_030068525.1	XP_030057759.1
381	AMP		Nanorana parkeri			XP_018414208.1	XP_018428789.1	
382	AMP		Rana temporaria	XP_040198845.1	XP_040191373.1	XP_040217737.1	XP_040200486.1	XP_040201804.1
383	AMP		Rhinatrema bivittatum	XP_029431025.1	XP_029446639.1	XP_029465783.1	XP_029439202.1	XP_029459854.1
384	AMP		Spea bombifrons	XP_053320752.1	XP_053314258.1	XP_053307327.1	XP_053318233.1	XP_053320082.1
385	AMP		Xenopus tropicalis	NP_001017330.1	XP_004911287.2	NP_001015925.1	XP_004912901.2	XP_012814227.2
386	ACT	14	Acanthochromis polyacanthus		XP_022055186.1	XP_022073150.2		
387	ACT	12	Acanthopagrus latus	XP_036952236.1	XP_036971574.1	XP_036979167.1	XP_036974957.1	
388	ACT	5	Alosa alosa	XP_048119571.1	XP_048108292.1	XP_048125260.1	XP_048086264.1	XP_048115360.1
389	ACT	5	Alosa sapidissima	XP_041918598.1	XP_041913914.1	XP_041921686.1	XP_041947779.1	XP_041915468.1
390	ACT	14	Amphiprion ocellaris	XP_023119749.1	XP_023122583.2	XP_023132439.2	XP_023132888.1	
391	ACT	13	Anabas testudineus	XP_026234681.1	XP_026201765.1	XP_026212744.1	XP_026226163.1	
392	ACT		Anarrhichthys ocellatus	XP_031704686.1		XP_031694356.1		
393	ACT	4	Anguilla anguilla		XP_035270963.1	XP_035256030.1		
394	ACT		Anoplopoma fimbria	XP_054467320.1		XP_054455811.1		
395	ACT	15	Archocentrus centrarchus		XP_030582521.1	XP_030603654.1	XP_030601563.1	
396	ACT	15	Astatotilapia calliptera		XP_026014148.1	XP_026045010.1	XP_026037012.1	

397	ACT	5	Astyanax mexicanus	XP_022541586.2	XP_007233466.3	XP_049337640.1	XP_049322243.1	
398	ACT	18	Austrofundulus limnaeus		XP_013876000.1	XP_013876621.1	XP_013887827.1	
399	ACT	13	Betta splendens	XP_029000918.1	XP_029003229.1	XP_029031760.1	XP_029029031.1	
400	ACT	11	Boleophthalmus pectinirostris	XP_020791825.1	XP_055020975.1	XP_055018813.1		
401	ACT	3	Brienomyrus brachyistius		XP_048864533.1	XP_048862555.1	XP_048850800.1	XP_048874687.1
402	ACT	5	Carassius auratus	XP_026123707.1	XP_026054964.1	XP_026113719.1	XP_026090069.1	
403	ACT	5	Carassius carassius	XP_059390372.1	XP_059383197.1	XP_059421795.1	XP_059382766.1	XP_059402689.1
404	ACT	5	Carassius gibelio		XP_052450783.1	XP_052428594.1		
405	ACT	12	Centropristis striata	XP_059210325.1	XP_059196663.1	XP_059214869.1	XP_059207151.1	
406	ACT		Chanos chanos	XP_030620893.1		XP_030626709.1	XP_030649425.1	XP_030624415.1
407	ACT	12	Cheilinus undulatus	XP_041643933.1	XP_041646739.1	XP_041646111.1	XP_041656846.1	
408	ACT	12	Chelmon rostratus	XP_041790919.1	XP_041791332.1	XP_041802999.1	XP_041807901.1	
409	ACT	5	Clarias gariepinus	XP_053348797.1	XP_053338598.1	XP_053357622.1	XP_053334147.1	
410	ACT	5	Clupea harengus	XP_012670327.2	XP_012676206.2	XP_031435304.1	XP_012693228.1	
411	ACT		Cololabis saira			XP_061601777.1	XP_061595463.1	
412	ACT	5	Colossoma macropomum	XP_036439952.1	XP_036446733.1	XP_036430451.1	XP_036444421.1	
413	ACT	4	Conger conger		XP_061094594.1	XP_061083959.1	XP_061104405.1	
414	ACT		Coregonus clupeaformis	XP_041715570.2		XP_045077253.1		
415	ACT	10	Corythoichthys intestinalis		XP_057696203.1	XP_057704437.1	XP_057676320.1	
416	ACT		Cottoperca gobio	XP_029282835.1		XP_029306428.1		
417	ACT	5	Ctenopharyngodon idella	XP_051756513.1	XP_051736588.1	XP_051773389.1	XP_051733542.1	XP_051748873.1
418	ACT	12	Cyclopterus lumpus	XP_034382911.1	XP_034387248.1	XP_034408686.1		
419	ACT		Cynoglossus semilaevis	XP_008335264.1		XP_008320374.1	XP_008330513.1	
420	ACT	19	Cyprinodon tularosa		XP_038127573.1	XP_038139995.1	XP_038148013.1	
421	ACT	19	Cyprinodon variegatus		XP_015259774.1	XP_015236475.1	XP_015229404.1	
422	ACT		Cyprinus carpio	XP_042584099.1		XP_042593369.1		XP_042580914.1
423	ACT	5	Danio aesculapii		XP_056305512.1	XP_056327538.1		XP_056314581.1
424	ACT	5	Danio rerio	NP_571502.2	XP_002666445.2	XP_009305819.1	NP_001338642.1	NP_997793.1
425	ACT	5	Denticeps clupeoides	XP_028812541.1	XP_028818727.1	XP_028844010.1	XP_028838973.1	XP_028828674.1
426	ACT	12	Dicentrarchus labrax	XP_051234327.1	XP_051232765.1	XP_051239957.1	XP_051256321.1	
427	ACT		Doryrhamphus excisus			XP_057920922.1	XP_057943091.1	
428	ACT		Dunckerocampus dactyliophorus			XP_054654802.1		
429	ACT	13	Echeneis naucrates	XP_029353864.1	XP_029355796.1	XP_029376677.1	XP_029375482.1	
430	ACT	5	Electrophorus electricus	XP_026860586.1	XP_035377273.1	XP_035387398.1	XP_026882186.1	
431	ACT	12	Epinephelus fuscoguttatus	XP_049452178.1	XP_049443439.1	XP_049457350.1	XP_049447664.1	
432	ACT	12	Epinephelus lanceolatus	XP_033479018.1	XP_033477212.1	XP_033468392.1	XP_033493643.1	
433	ACT		Epinephelus moara	XP_049909967.1	XP_049910804.1	XP_049928038.1	XP_049892707.1	
434	ACT	1	Erpetoichthys calabaricus		XP_028657633.2	XP_028651180.1	XP_028668520.1	
435	ACT	6	Esox lucius	XP_010873809.2	XP_010870438.2	XP_019902789.1	XP_010869440.1	XP_010876423.1
436	ACT	12	Etheostoma cragini	XP_034736903.1	XP_034737522.1	XP_034755139.1	XP_034745181.1	
437	ACT	12	Etheostoma spectabile	XP_032373448.1	XP_032380934.1	XP_032396926.1	XP_032390453.1	
438	ACT	19	Fundulus heteroclitus		XP_012716720.2		XP_035998788.1	
439	ACT		Gadus chalcogrammus	XP_056463585.1		XP_056465010.1		XP_056444171.1
440	ACT		Gadus macrocephalus	XP_059927063.1			XP_059911701.1	
441	ACT		Gadus morhua	XP_030233454.1		XP_030234988.1	XP_030216505.1	
442	ACT	21	Gambusia affinis		XP_043985013.1	XP_043991871.1	XP_043996165.1	
443	ACT	12	Gasterosteus aculeatus	XP_040019161.1	XP_040040336.1	XP_040034009.1	XP_040036561.1	
444	ACT	19	Girardinichthys multiradiatus		XP_047230856.1	XP_047207341.1	XP_047235553.1	
445	ACT	14	Gouania willdenowi		XP_028299967.1	XP_028323954.1	XP_028323360.1	
446	ACT		Gymnodraco acuticeps	XP_034076922.1		XP_034079684.1	XP_034062596.1	
447	ACT	15	Haplochromis burtoni	XP_005942578.1	XP_005923083.1	XP_005933665.1	XP_005926456.1	
448	ACT	5	Hemibagrus wyckioides	XP_058237343.1	XP_058266433.1	XP_058236954.1	XP_058271160.1	
449	ACT		Hippocampus comes			XP_019737493.1	XP_019719727.1	
450	ACT	10	Hippocampus zosterae		XP_051929819.1	XP_051936262.1	XP_051902577.1	
451	ACT	13	Hippoglossus hippoglossus	XP_034431566.1	XP_034438826.1	XP_034465202.1	XP_034461604.1	
452	ACT	13	Hippoglossus stenolepis	XP_035033028.1	XP_035032034.1	XP_035027903.1	XP_035035404.1	
453	ACT		Hypomesus transpacificus	XP_046898289.1		XP_046885529.1	XP_046899015.1	XP_046873871.1
454	ACT	5	Ictalurus furcatus		XP_053499226.1	XP_053477668.1	XP_053504527.1	
455	ACT	5	Ictalurus punctatus	XP_017313831.1	XP_017343478.1	XP_017319134.1	XP_017348373.1	
456	ACT	18	Kryptolebias marmoratus		XP_017278985.1			
457	ACT	5	Labeo rohita	XP_050972146.1	XP_050952257.1	XP_050981550.1	XP_050949852.1	XP_050966556.1
458	ACT	12	Labrus bergylta	XP_020493352.1	XP_020487405.1	XP_020508933.2	XP_020506765.1	
459	ACT	12	Labrus mixtus	XP_060883630.1	XP_060885519.1	XP_060896711.1	XP_060911176.1	
460	ACT	7	Lampris incognitus	XP_056134105.1	XP_056131730.1	XP_056148192.1		XP_056154075.1
461	ACT	12	Larimichthys crocea	XP_027137622.1	XP_027146085.1	XP_019128344.2	XP_019127684.1	
462	ACT	13	Lates calcarifer	XP_018538951.1	XP_018525508.1	XP_018536581.1	XP_018547360.1	
463	ACT	2	Lepisosteus oculatus		XP_006629654.2	XP_015202149.1	XP_015204853.1	XP_015193092.1
464	ACT		Limanda limanda	XP_060924657.1		XP_060942936.1	XP_060941352.1	
465	ACT	13	Mastacembelus armatus	XP_026175839.1	XP_026160844.1	XP_026164629.1	XP_026185051.1	
466	ACT	15	Maylandia zebra		XP_004557145.1	XP_004554601.1	XP_004550383.1	
467	ACT	5	Megalobrama amblycephala	XP_048041159.1	XP_048033498.1	XP_048060429.1	XP_048022690.1	XP_048045945.1
468	ACT	4	Megalops cyprinoides		XP_036377753.1	XP_036402159.1	XP_036379375.1	XP_036384262.1
469	ACT	16	Melanotaenia boesemani	XP_041823630.1	XP_041861356.1	XP_041865505.1	XP_041863854.1	
470	ACT	12	Micropterus dolomieu	XP_045888596.1	XP_045906191.1	XP_045926629.1	XP_045914177.1	
471	ACT	12	Micropterus salmoides	XP_038558195.1	XP_038572483.1	XP_038593227.1	XP_038563475.1	
472	ACT	5	Misgurnus anguillicaudatus	XP_055064622.1	XP_055043892.1	XP_055027018.1	XP_055035288.1	XP_055056320.1
473	ACT	13	Monopterus albus	XP_020461444.1	XP_020459028.1	XP_020454003.1	XP_020455510.1	
474	ACT	12	Morone saxatilis	XP_035537814.1	XP_035509826.1	XP_035519990.1	XP_035521920.1	
475	ACT	14	Mugil cephalus	XP_047437345.1	XP_047443047.1	XP_047459925.1	XP_047462651.1	
476	ACT	8	Myripristis murdjan	XP_029904294.1	XP_029905350.1	XP_029926269.1	XP_029924068.1	
477	ACT	18	Nematolebias whitei		XP_037554279.1		XP_037543829.1	
478	ACT	5	Neoarius graeffei	XP_060768042.1	XP_060793668.1	XP_060782219.1	XP_060791886.1	
479	ACT	15	Neolamprologus brichardi		XP_006790297.1	XP_006799105.1	XP_006785009.1	
480	ACT	18	Nothobranchius furzeri	XP_015809140.1	XP_015831822.1		XP_015816779.1	
481	ACT	12	Notolabrus celidotus	XP_034535599.1	XP_034557438.1	XP_034538513.1	XP_034539580.1	
482	ACT		Notothenia coriiceps	XP_010782123.1				XP_010794484.1
483	ACT		Oncorhynchus gorbuscha	XP_046189338.1		XP_046209593.1		
484	ACT		Oncorhynchus keta	XP_052371795.1		XP_035655131.1		XP_035631649.1
485	ACT		Oncorhynchus kisutch	XP_031668571.1		XP_020350592.1		
486	ACT		Oncorhynchus mykiss			XP_036815818.1		
487	ACT		Oncorhynchus tshawytscha	XP_042173443.1		XP_024280430.1		
488	ACT	5	Onychostoma macrolepis	XP_058639405.1	XP_058619530.1	XP_058651556.1	XP_058613544.1	XP_058633054.1
489	ACT	15	Oreochromis aureus		XP_031609612.1	XP_039477703.1	XP_031604361.1	
490	ACT	15	Oreochromis niloticus		XP_003439828.1	XP_025753223.1	XP_013127891.1	
491	ACT	17	Oryzias latipes		XP_011472752.1	XP_023818930.1	XP_023818691.1	
492	ACT		Oryzias melastigma		XP_024134822.1	XP_024120653.1	XP_024122309.1	
493	ACT	5	Pangasianodon hypophthalmus	XP_026793231.1	XP_026776216.1	XP_034158603.2	XP_026796735.1	
494	ACT	13	Paralichthys olivaceus		XP_019936897.1	XP_019953966.1	XP_019952722.1	
495	ACT	14	Parambassis ranga	XP_028258397.1	XP_028259223.1	XP_028278534.1	XP_028276660.1	XP_028269821.1
496	ACT	3	Paramormyrops kingsleyae		XP_023679554.1	XP_023679938.1	XP_023652679.1	XP_023674477.1

497	ACT	12	Perca flavescens	XP_028438054.1	XP_028442559.1	XP_028459072.1	XP_028450864.1	
498	ACT	12	Perca fluviatilis	XP_039651657.1	XP_039667560.1	XP_039640845.1	XP_039632738.1	
499	ACT	11	Periophthalmus magnuspinnatus		XP_033820728.1	XP_033835901.1	XP_033834638.1	
500	ACT		Phycodurus eques			XP_061545662.1	XP_061557919.1	
501	ACT		Phyllopteryx taeniolatus			XP_061645274.1	XP_061607305.1	
502	ACT		Pimephales promelas	XP_039513892.1		XP_039527641.1		XP_039517977.1
503	ACT	12	Plectropomus leopardus		XP_042339492.1	XP_042357508.1	XP_042354603.1	
504	ACT	13	Pleuronectes platessa		XP_053286516.1	XP_053292340.1	XP_053296937.1	
505	ACT	20	Poecilia formosa	XP_007549083.1	XP_007550811.1	XP_007567014.1	XP_007552997.1	
506	ACT	20	Poecilia latipinna	XP_014909318.1	XP_014899440.1	XP_014913720.1		
507	ACT	20	Poecilia mexicana		XP_014828802.1	XP_014863520.1	XP_014864742.1	
508	ACT	20	Poecilia reticulata		XP_008405750.1	XP_017164581.1	XP_008425179.1	
509	ACT	21	Poeciliopsis prolifica		XP_054916462.1	XP_054877715.1	XP_054907334.1	
510	ACT	1	Polypterus senegalus		XP_039607441.1	XP_039626755.1	XP_039631792.1	
511	ACT		Pseudochaenichthys georgianus	XP_033935163.1		XP_033957300.1		XP_033951573.1
512	ACT		Pseudoliparis swirei	XP_056269134.1		XP_056292295.1	XP_056298466.1	
513	ACT	15	Pundamilia nyererei		XP_005732398.1	XP_013766833.1	XP_005727991.1	
514	ACT	12	Pungitius pungitius	XP_037307157.1	XP_037325378.1	XP_037321189.1	XP_037322632.1	
515	ACT	5	Puntigrus tetrazona	XP_043099292.1	XP_043080831.1	XP_043111607.1	XP_043076761.1	XP_043095340.1
516	ACT	5	Pygocentrus nattereri	XP_017571897.1	XP_017566953.1	XP_017552165.1	XP_017576729.1	
517	ACT		Rhinichthys klamathensis	XP_056098458.1	XP_056096400.1	XP_056104195.1	XP_056123806.1	XP_056108938.1
518	ACT	14	Salarias fasciatus	XP_029965344.1	XP_029939893.1	XP_029963413.1		
519	ACT		Salmo salar			XP_014055433.1		
520	ACT		Salmo trutta			XP_029553923.1		
521	ACT		Salvelinus namaycush			XP_038847686.1		
522	ACT	12	Sander lucioperca	XP_031145898.1	XP_031167551.1	XP_031152733.1	XP_031136476.1	
523	ACT	12	Scatophagus argus	XP_046249914.1	XP_046248178.1	XP_046257504.1	XP_046266069.1	XP_046231265.1
524	ACT		Scleropages formosus			XP_018593581.1	XP_018607541.1	XP_018600431.1
525	ACT	10	Scomber japonicus	XP_053174215.1	XP_053177908.1	XP_053188425.1	XP_053187291.1	XP_053196606.1
526	ACT	13	Scophthalmus maximus	XP_035485313.1	XP_035504113.2	XP_035462660.2	XP_035480797.2	
527	ACT	12	Sebastes umbrosus	XP_037615364.1	XP_037625949.1	XP_037637909.1	XP_037603526.1	
528	ACT		Seriola aureovittata	XP_056239494.1	XP_056234123.1	XP_056251482.1	XP_056252806.1	
529	ACT	13	Seriola dumerili		XP_022614797.1	XP_022599500.1	XP_022594496.1	
530	ACT	13	Seriola lalandi	XP_023257429.1	XP_023284646.1	XP_023281618.1	XP_023285126.1	
531	ACT	5	Silurus meridionalis	XP_046697027.1	XP_046725236.1	XP_046726311.1	XP_046723491.1	
532	ACT	15	Simochromis diagramma		XP_039896067.1	XP_039900595.1	XP_039888073.1	
533	ACT		Siniperca chuatsi	XP_044049250.1		XP_044071146.1	XP_044077841.1	
534	ACT	5	Sinocyclocheilus rhinocerous	XP_016396548.1	XP_016387006.1	XP_016417231.1		XP_016422416.1
535	ACT	13	Solea senegalensis	XP_043886555.1	XP_043900478.1	XP_043902275.1	XP_043897742.1	XP_043868085.1
536	ACT		Solea solea	XP_058486415.1		XP_058507641.1	XP_058482949.1	XP_058473778.1
537	ACT	12	Sparus aurata	XP_030271118.1	XP_030289186.1	XP_030297885.1	XP_030293266.1	
538	ACT	11	Sphaeramia orbicularis		XP_029987376.1	XP_030011746.1	XP_030010817.1	XP_030004686.1
539	ACT	14	Stegastes partitus	XP_008304839.1	XP_008297065.1	XP_008278396.1	XP_008301012.1	XP_008286794.1
540	ACT	10	Synchiropus splendidus		XP_053741609.1	XP_053731534.1	XP_053702766.1	XP_053709184.1
541	ACT		Syngnathoides biaculeatus			XP_061692380.1	XP_061659437.1	
542	ACT	10	Syngnathus acus		XP_037104676.1	XP_037126784.1	XP_037125441.1	
543	ACT	10	Syngnathus scovelli		XP_049575250.1	XP_049592656.1	XP_049597671.1	
544	ACT	10	Syngnathus typhle		XP_061152537.1	XP_061140521.1		
545	ACT	5	Tachysurus fulvidraco	XP_047675801.1	XP_047662946.1	XP_027030677.1	XP_026995713.1	
546	ACT	5	Tachysurus vachellii	XP_060715259.1	XP_060749957.1	XP_060728760.1	XP_060747591.1	
547	ACT	12	Takifugu flavidus		XP_056894061.1	XP_056904183.1	XP_056909929.1	
548	ACT	12	Takifugu rubripes		XP_029684566.1	XP_029690974.1	XP_011609429.2	XP_003978167.1
549	ACT	9	Thalassophryne amazonica		XP_034036442.1	XP_034041245.1	XP_034033956.1	
550	ACT	10	Thunnus albacares	XP_044210935.1	XP_044217041.1	XP_044228257.1	XP_044225988.1	
551	ACT	10	Thunnus maccoyii	XP_042269718.1	XP_042273346.1	XP_042290301.1	XP_042286944.1	
552	ACT	13	Toxotes jaculatrix	XP_040891137.1	XP_040895743.1	XP_040905339.1	XP_040907052.1	
553	ACT		Trematomus bernacchii	XP_033971855.1		XP_033977750.1	XP_033995786.1	
554	ACT		Triplophysa dalaica	XP_056605592.1	XP_056588847.1	XP_056616271.1	XP_056586390.1	XP_056601921.1
555	ACT	5	Triplophysa rosa	XP_057193605.1	XP_057219373.1	XP_057197321.1	XP_057215795.1	XP_057177374.1
556	ACT	13	Xiphias gladius	XP_039992344.1	XP_039984972.1	XP_039995935.1	XP_040005800.1	
557	ACT	21	Xiphophorus couchianus		XP_027883476.1	XP_027862517.1	XP_027886604.1	
558	ACT	21	Xiphophorus hellerii		XP_032427620.1		XP_032431751.1	
559	ACT	21	Xiphophorus maculatus		XP_023195334.1	XP_023183344.1	XP_014326871.1	
560	ACT	5	Xyrauchen texanus		XP_051947575.1			
561	CHO		Amblyraja radiata	XP_032870937.1	XP_032881590.1	XP_032889501.1	XP_032885535.1	XP_032869548.1
562	CHO		Callorhinchus milii			XP_007894691.2	XP_042195821.1	XP_007909778.1
563	CHO		Carcharodon carcharias			XP_041065510.1	XP_041049286.1	
564	CHO		Chiloscyllium plagiosum			XP_043568437.1	XP_043559789.1	XP_043537546.1
565	CHO		Hemiscyllium ocellatum		XP_060681370.1	XP_060697908.1	XP_060693066.1	
566	CHO		Hypanus sabinus		XP_059844879.1	XP_059803580.1	XP_059846135.1	XP_059819325.1
567	CHO		Leucoraja erinacea		XP_055494802.1	XP_055502602.1	XP_055498932.1	
568	CHO		Pristis pectinata			XP_051883543.1	XP_051869672.1	XP_051896732.1
569	CHO		Rhincodon typus				XP_048459683.1	XP_020392043.1
570	CHO		Scyliorhinus canicula		XP_038647543.1	XP_038677256.1	XP_038651400.1	XP_038641598.1
571	CHO		Stegostoma tigrinum			XP_048406865.1	XP_048399151.1	XP_048378692.1
