# Supplementary material for: Divergent evolution of low-complexity regions in the vertebrate CPEB protein family
Source: Front Bioinform. 2025 Mar 20;5:1491735. doi: 10.3389/fbinf.2025.1491735 (PMC11965684; doi:10.3389/fbinf.2025.1491735)
Supplement: Supplementary file 3 [file Presentation1.pdf]

Supplemental materials

***

Divergent evolution of low-complexity regions in the vertebrate CPEB protein family

Serena Vaglietti[1], Stefania Boggio Bozzo[1], Mirella Ghirardi[1] and Ferdinando Fiumara[1]*

[1]*Rita Levi Montalcini* Department of Neuroscience, University of Turin, 10125, Turin, Italy

**Correspondence*: ferdinando.fiumara@unito.it

SUPPLEMENTAL FIGURE LEGENDS

Suppl. Figure 1. Distributions of the 20 amino acids along the primary sequence of human CPEB1

Schematic representation of the human CPEB1 protein, as in *Figure 2,* with the distribution of all 20 amino acids along the protein primary sequence, together with a schematic representation of the location of AARs and graphs of the per-residue SIM, REP, FuzDrop P_{DP} and PLAAC PrD scores.

Suppl. Figure 2. Distributions of the 20 amino acids along the primary sequence of human CPEB2

As in *Suppl. Fig. 1* for CPEB2.

Suppl. Figure 3. Distributions of the 20 amino acids along the primary sequence of human CPEB3

As in *Suppl. Fig. 1* for CPEB3.

Suppl. Figure 4. Distributions of the 20 amino acids along the primary sequence of human CPEB4.

As in *Suppl. Fig. 1* for CPEB4.

Suppl. Figure 5. Evolutionary dynamics of CPEB2 composition- and function-related parameters

Scatterplots with regression lines displaying correlations between two sets of 24 Pearson's *r* coefficients. These two sets were calculated for correlations between clade stem age and the 24 parameters of interest (percent occurrence of the 20 amino acids as well as SIM, REP, LLPS propensity, and prion-likeness scores) by considering, in each clade, *either* all the available CPEB2 ortholog sequences *or* only those from five randomly selected species. The scatterplots are relative to the 10 replicates of the same analysis (*Runs 1-10*) that were performed with different sets of randomly selected species per clade. Remarkably, for each replicate, we found a strong correlation ($r > 0.99$, n = 24, $p < 0.01$ in all instances) between the results (i.e. the *r* coefficients) of the evolutionary analyses performed using, for each clade, either all the available sequences or only 5 randomly selected ones.

Suppl. Figure 6. Intraclade *versus* interclade evolutionary variation in CPEB2

A-B. As in Fig. 9*B* for REP and PrD scores.

Suppl. Figure 7. Evolutionary dynamics of TIA1 compositional features, LLPS propensity, and prion-likeness in vertebrates

A. Schematic representation of the TIA1 protein highlighting the three RRMs (RRM1-3) in the N-terminal region (NTR) and the low-complexity C-terminal region (CTR). **B.** *Fig. 5E* panel reproduced here for comparison with *panel C*. **C.** As in *panel B* for TIA1. The graph reports the values related only to those amino acids displaying evolutionary variation that correlated significantly with clade stem ages. Note how the evolutionary variation of amino acid occurrence for TIA1 tends to be overall more limited than that observed for CPEB2. **D-G.** As in *Fig. 9C* for the indicated parameters.

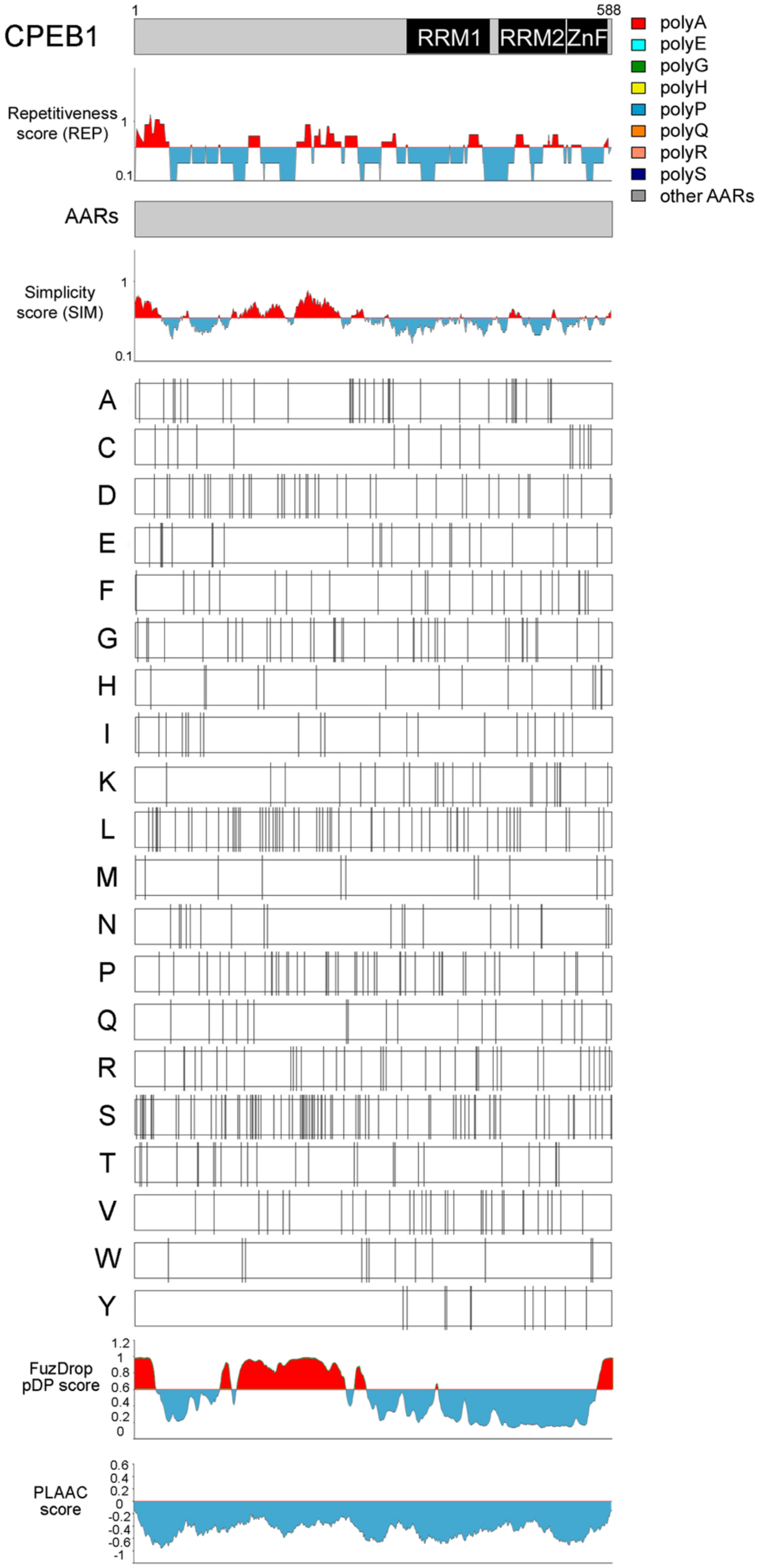
CPEB1
1
588
RRM1
RRM2
ZnF
polyA
polyE
polyG
polyH
polyP
polyQ
polyR
polyS
other AARs
Repetitiveness score (REP)
AARs
Simplicity score (SIM)
A
C
D
E
F
G
H
I
K
L
M
N
P
Q
R
S
T
V
W
Y
FuzDrop pDP score
PLAAC score

Suppl. Fig. 1

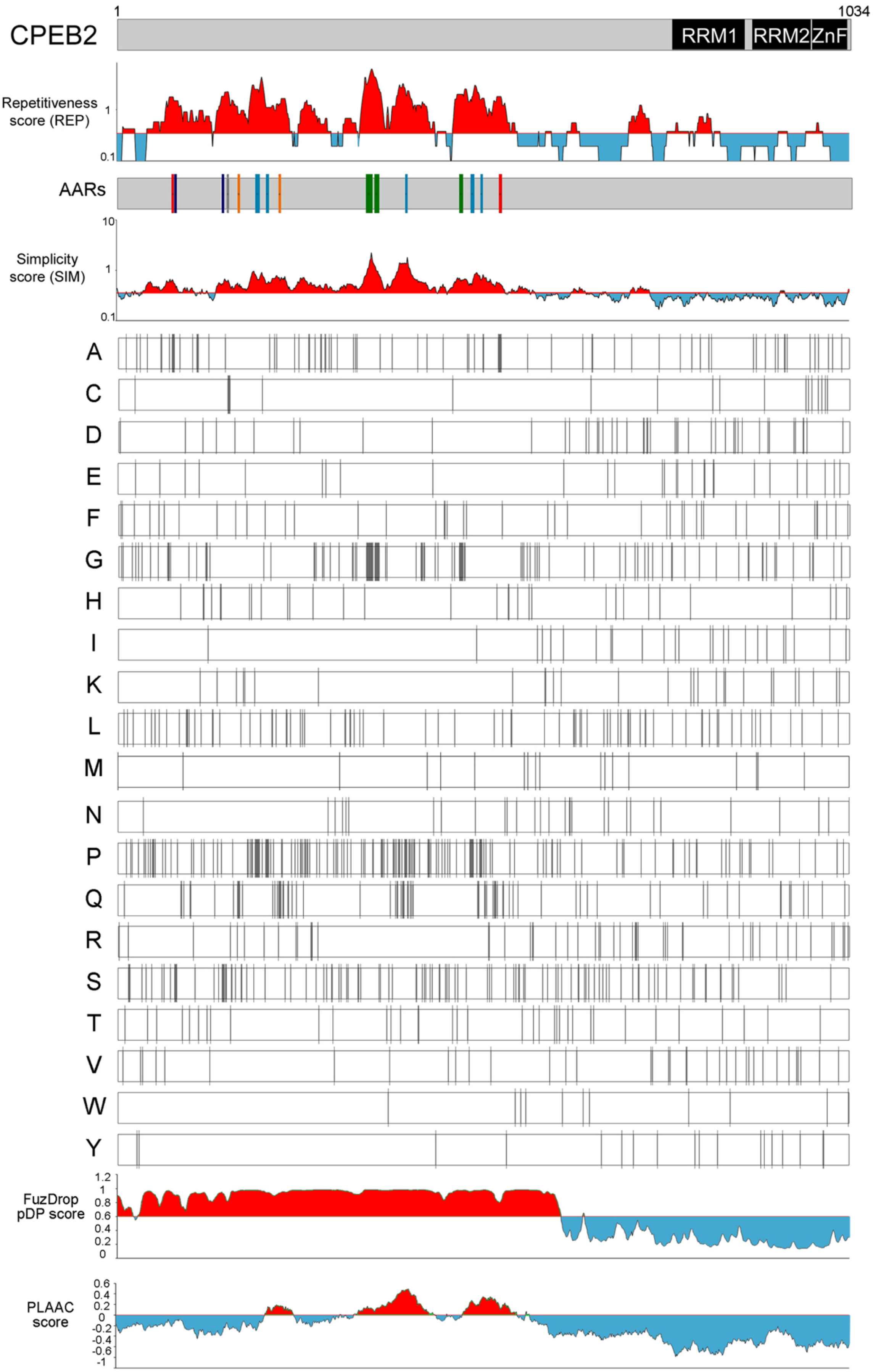
1
1034
CPEB2
RRM1
RRM2
ZnF
Repetitiveness score (REP)
1
0.1
AARs
10
Simplicity score (SIM)
1
0.1
A
C
D
E
F
G
H
I
K
L
M
N
P
Q
R
S
T
V
W
Y
FuzDrop pDP score
1.2
1
0.8
0.6
0.4
0.2
0
PLAAC score
0.6
0.4
0.2
0
-0.2
-0.4
-0.6
-1

Suppl. Fig. 2

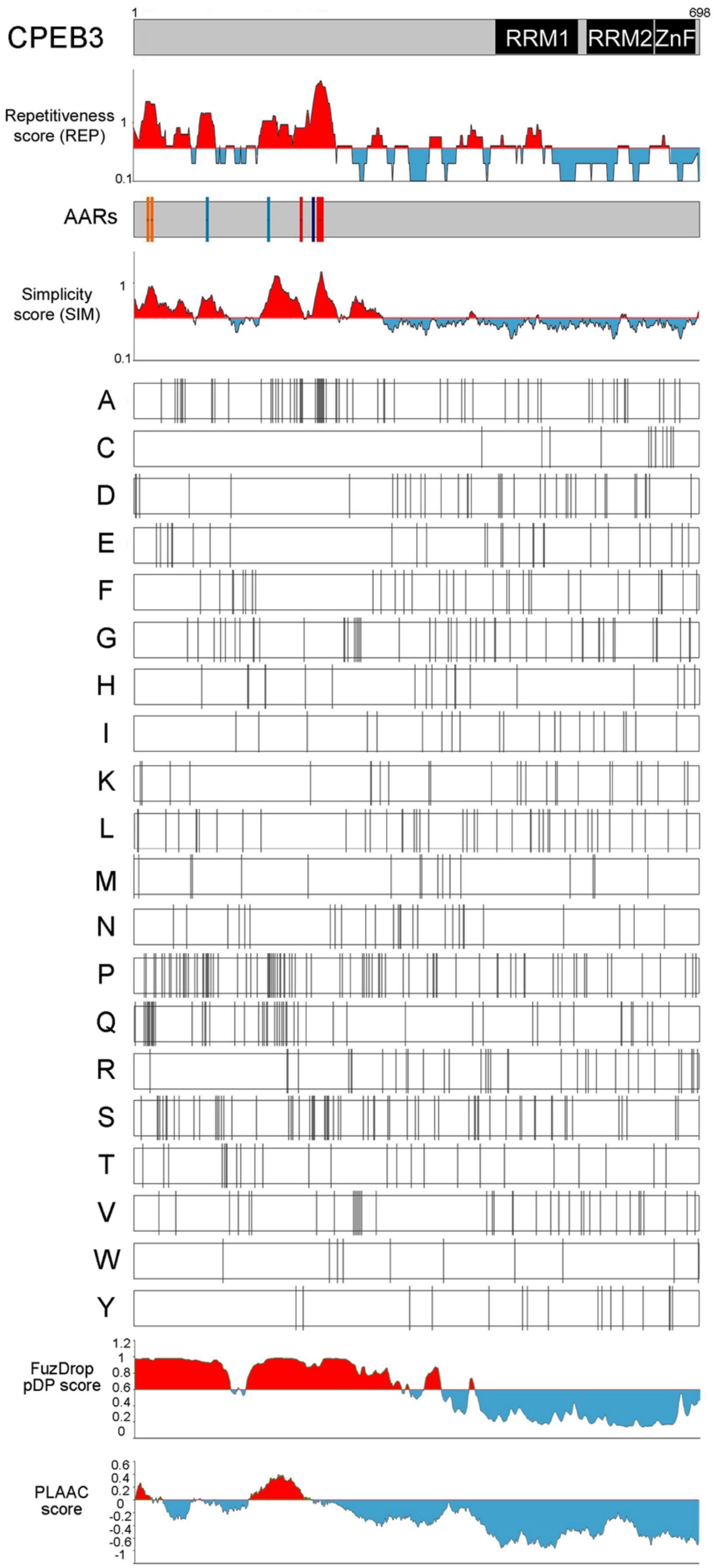
CPEB3
1
698
RRM1
RRM2
ZnF
Repetitiveness score (REP)
1
0.1
AARs
Simplicity score (SIM)
1
0.1
A
C
D
E
F
G
H
I
K
L
M
N
P
Q
R
S
T
V
W
Y
FuzDrop pDP score
1.2
1
0.8
0.6
0.4
0.2
0
PLAAC score
0.6
0.4
0.2
0
-0.2
-0.4
-0.6
-1

Suppl. Fig. 3

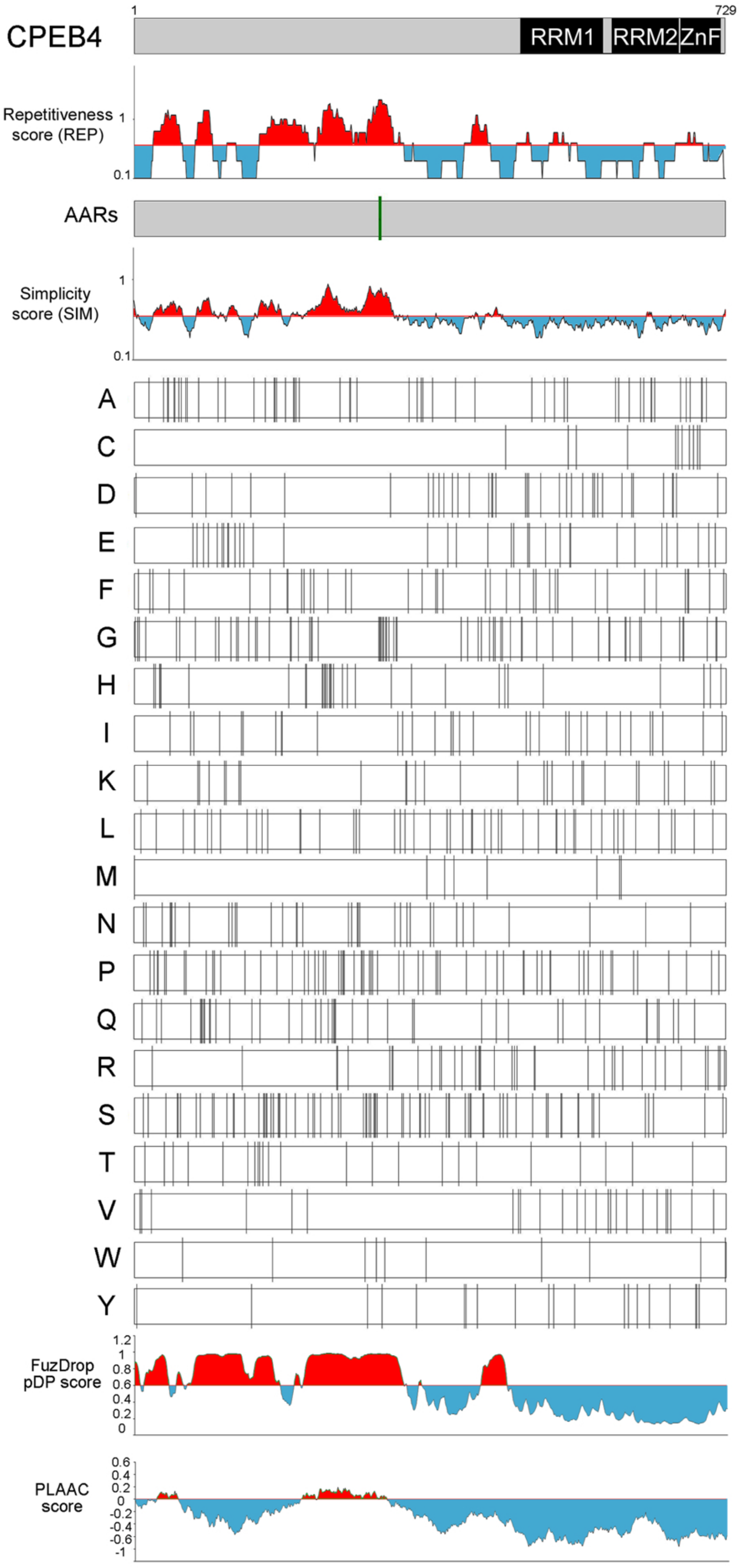

CPEB4
1
729
RRM1
RRM2
ZnF
Repetitiveness score (REP)
1
0.1
AARs
Simplicity score (SIM)
1
0.1
A
C
D
E
F
G
H
I
K
L
M
N
P
Q
R
S
T
V
W
Y
FuzDrop pDP score
1.2
1
0.8
0.6
0.4
0.2
0
PLAAC score
0.6
0.4
0.2
0
-0.2
-0.4
-0.6
-1

Suppl. Fig. 4

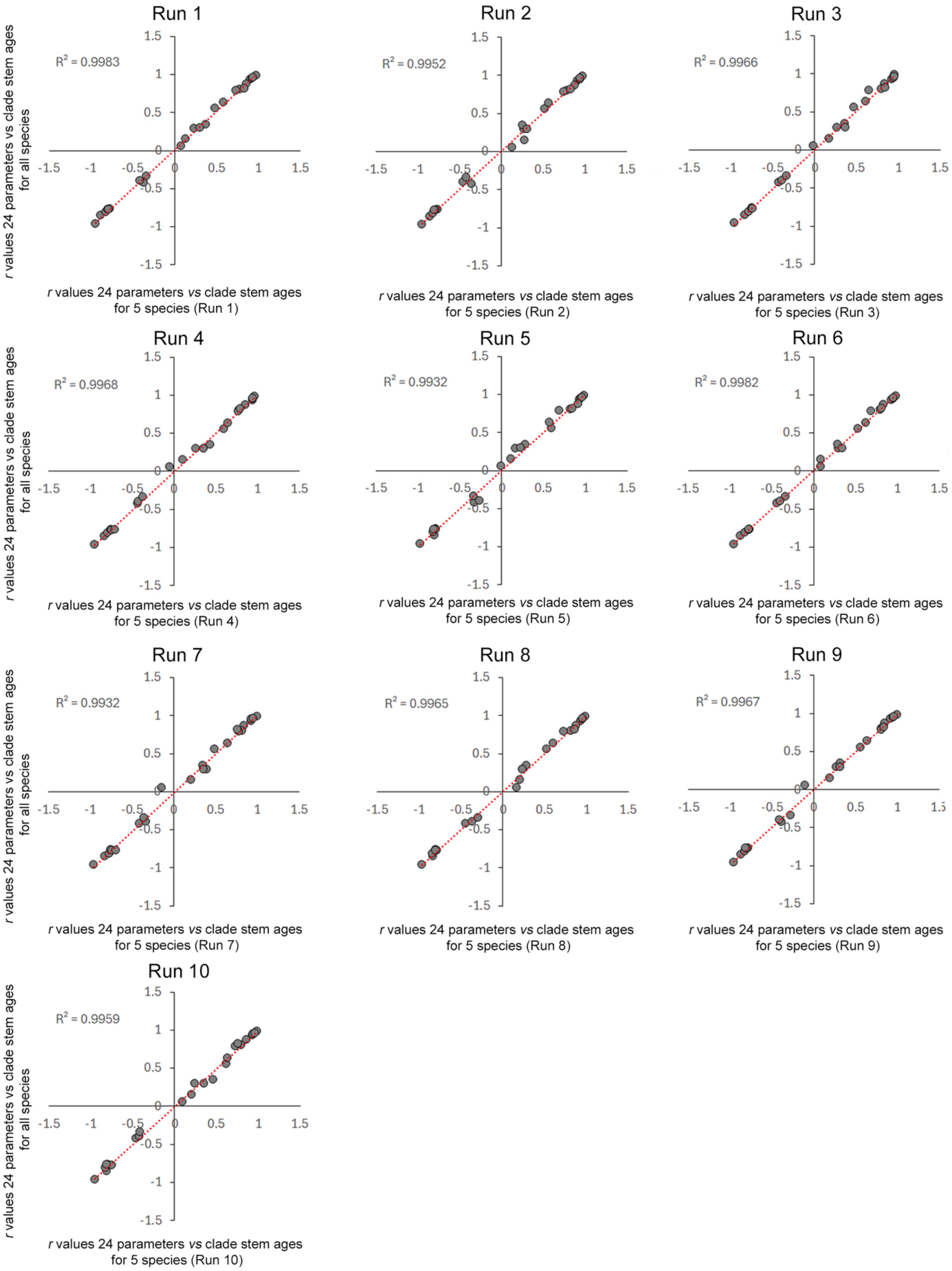
Run 1
R² = 0.9983
r values 24 parameters vs clade stem ages for all species
r values 24 parameters vs clade stem ages for 5 species (Run 1)
Run 2
R² = 0.9952
r values 24 parameters vs clade stem ages for 5 species (Run 2)
Run 3
R² = 0.9966
r values 24 parameters vs clade stem ages for 5 species (Run 3)
Run 4
R² = 0.9968
r values 24 parameters vs clade stem ages for all species
r values 24 parameters vs clade stem ages for 5 species (Run 4)
Run 5
R² = 0.9932
r values 24 parameters vs clade stem ages for 5 species (Run 5)
Run 6
R² = 0.9982
r values 24 parameters vs clade stem ages for 5 species (Run 6)
Run 7
R² = 0.9932
r values 24 parameters vs clade stem ages for all species
r values 24 parameters vs clade stem ages for 5 species (Run 7)
Run 8
R² = 0.9965
r values 24 parameters vs clade stem ages for 5 species (Run 8)
Run 9
R² = 0.9967
r values 24 parameters vs clade stem ages for 5 species (Run 9)
Run 10
R² = 0.9959
r values 24 parameters vs clade stem ages for all species
r values 24 parameters vs clade stem ages for 5 species (Run 10)

Suppl. Fig. 5

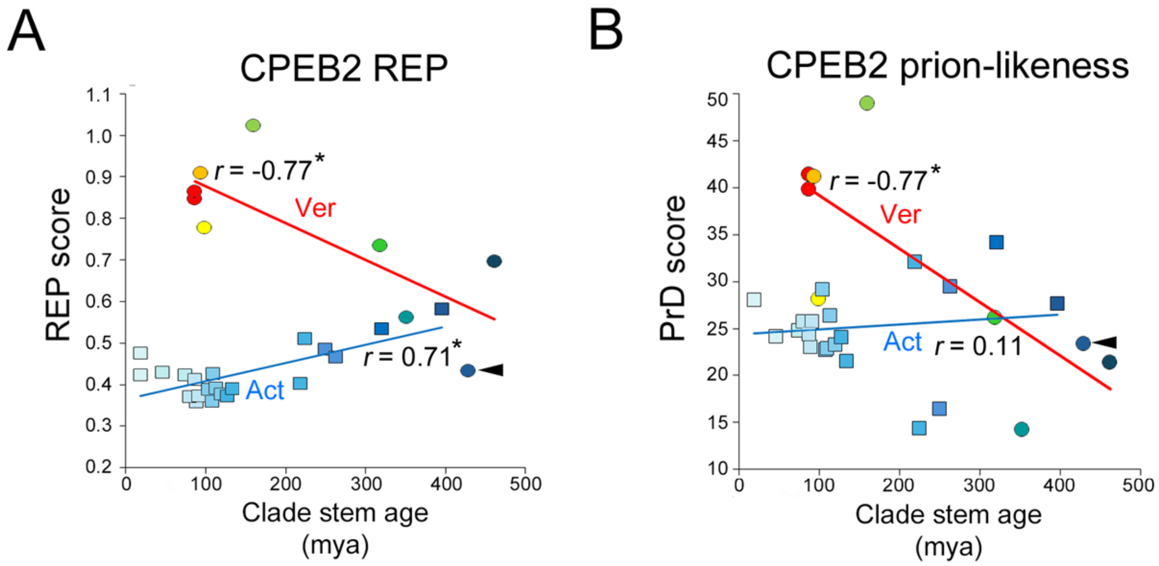
A
CPEB2 REP
REP score
r = -0.77*
Ver
r = 0.71*
Act
Clade stem age
(mya)
B
CPEB2 prion-likeness
PrD score
r = -0.77*
Ver
Act
r = 0.11
Clade stem age
(mya)

Suppl. Fig. 6

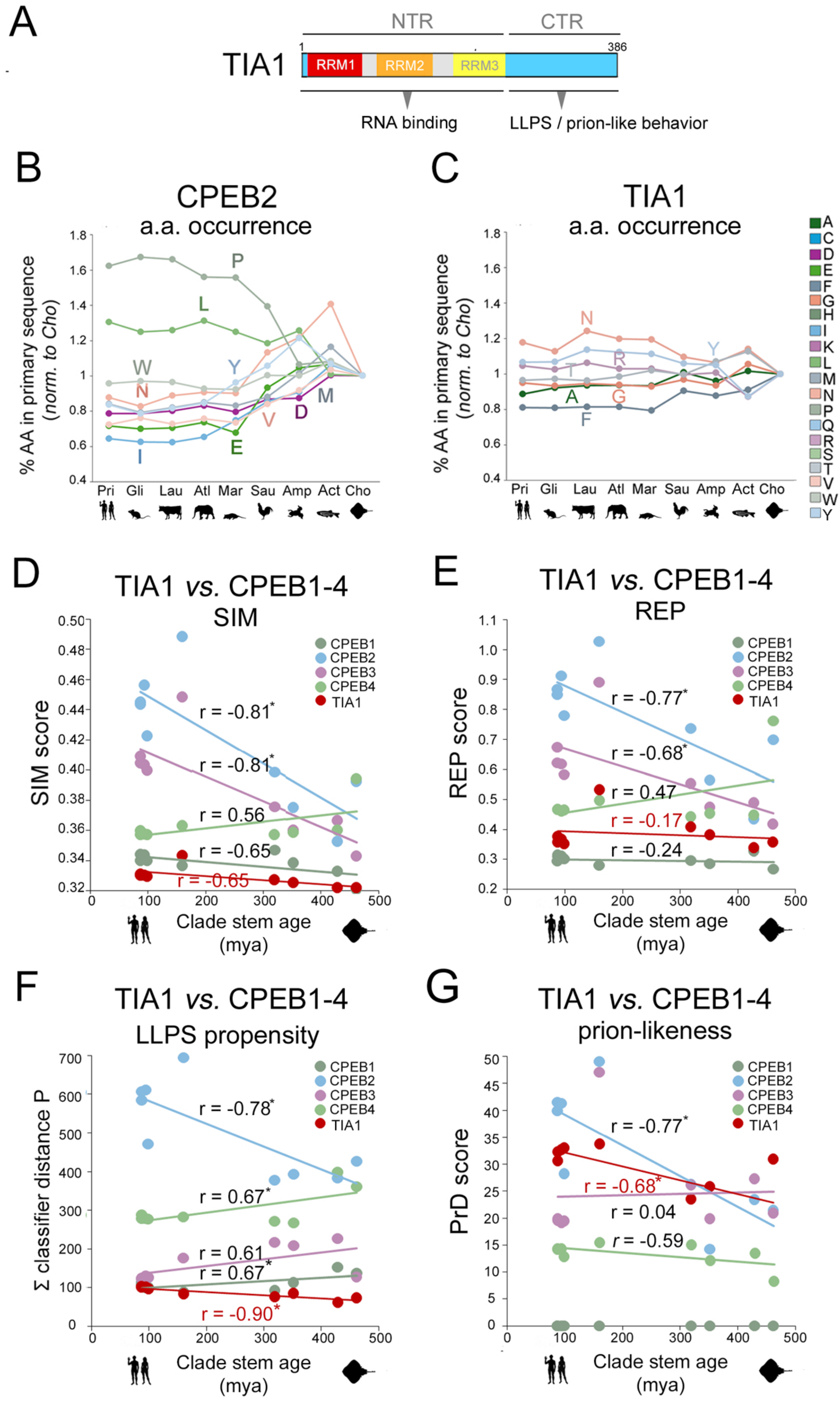
A
TIA1
NTR
CTR
RRM1
RRM2
RRM3
1
386
RNA binding
LLPS / prion-like behavior
B
CPEB2
a.a. occurrence
C
TIA1
a.a. occurrence
% AA in primary sequence (norm. to Cho)
Pri Gli Lau Atl Mar Sau Amp Act Cho
D
TIA1 vs. CPEB1-4
SIM
SIM score
r = -0.81*
r = -0.81*
r = 0.56
r = -0.65
r = -0.65
E
TIA1 vs. CPEB1-4
REP
REP score
r = -0.77*
r = -0.68*
r = 0.47
r = -0.17
r = -0.24
F
TIA1 vs. CPEB1-4
LLPS propensity
Σ classifier distance P
r = -0.78*
r = 0.67*
r = 0.61
r = 0.67*
r = -0.90*
G
TIA1 vs. CPEB1-4
prion-likeness
PrD score
r = -0.77*
r = -0.68*
r = 0.04
r = -0.59
CPEB1
CPEB2
CPEB3
CPEB4
TIA1
Clade stem age (mya)

Suppl. Fig. 7
