# Supplementary material for: Divergent evolution of low-complexity regions in the vertebrate CPEB protein family
Source: Front Bioinform. 2025 Mar 20;5:1491735. doi: 10.3389/fbinf.2025.1491735 (PMC11965684; doi:10.3389/fbinf.2025.1491735)
Supplement: Supplementary file 4 [file Table2.pdf]

**Supplemental Table 2** - *Pearson's r coefficients* for correlations between the indicated parameters (mean value per clade) and clade stem ages for each CPEB paralog*

| Paralogs | % A | % C | % D | % E | % F | % G | % H | % I | % K | % L | % M | % N | % P | % Q | % R | % S | %T | % V | % W | % Y |
|---|---|---|---|---|---|---|---|---|---|---|---|---|---|---|---|---|---|---|---|---|
| CPEB1 | -0.69 | 0.63 | 0.38 | -0.78 | -0.04 | 0.10 | -0.76 | -0.10 | 0.25 | -0.73 | 0.86 | 0.72 | -0.53 | 0.55 | -0.28 | 0.52 | 0.53 | -0.15 | -0.41 | 0.55 |
| CPEB2 | -0.43 | 0.29 | 0.93 | 0.95 | 0.55 | -0.40 | 0.34 | 0.98 | 0.63 | -0.85 | 0.87 | 0.80 | -0.96 | -0.34 | 0.29 | 0.15 | 0.05 | 0.96 | 0.78 | 0.82 |
| CPEB3 | -0.93 | 0.83 | 0.87 | 0.60 | 0.77 | 0.54 | 0.41 | 0.96 | 0.90 | 0.52 | 0.43 | 0.96 | -0.93 | -0.41 | 0.92 | 0.11 | 0.62 | 0.59 | 0.92 | 0.65 |
| CPEB4 | -0.75 | 0.64 | -0.23 | -0.29 | -0.23 | 0.08 | 0.35 | -0.82 | -0.28 | -0.31 | 0.76 | -0.36 | 0.51 | 0.23 | 0.30 | 0.26 | 0.48 | 0.84 | -0.04 | -0.66 |

| Paralogs | pA | pC | pD | pE | pF | pG | pH | pI | pK | pL | pM | pN | pP | pQ | pR | pS | pT | pV | pW | pY | SIM | REP | LLPS | PRD |
|---|---|---|---|---|---|---|---|---|---|---|---|---|---|---|---|---|---|---|---|---|---|---|---|---|
| CPEB1 | -0.27 | NA | NA | -0.52 | NA | 0.30 | 0.47 | NA | NA | 0.21 | NA | 0.47 | 0.47 | NA | -0.51 | 0.47 | 0.47 | NA | NA | NA | -0.65 | -0.24 | 0.67 | NA |
| CPEB2 | -0.60 | -0.85 | 0.47 | 0.76 | NA | -0.88 | 0.44 | NA | NA | 0.64 | NA | 0.82 | -0.91 | -0.09 | 0.09 | 0.03 | 0.47 | 0.55 | 0.55 | 0.55 | -0.81 | -0.77 | -0.78 | -0.77 |
| CPEB3 | -0.84 | NA | NA | NA | NA | 0.47 | 0.38 | NA | 0.47 | NA | NA | 0.47 | -0.60 | -0.07 | 0.21 | 0.23 | 0.47 | NA | NA | NA | -0.81 | -0.68 | 0.61 | 0.04 |
| CPEB4 | 0.22 | NA | NA | NA | NA | 0.76 | 0.55 | NA | NA | NA | NA | NA | 0.13 | 0.75 | NA | 0.21 | 0.47 | NA | NA | NA | 0.56 | 0.47 | 0.67 | -0.59 |

*the *r* value for statistically significant changes is in *green* (increase from older to younger clades) or in *red* (decrease from older to younger clades).

**Legend**

*%X = mean percent amino acid frequency across ortholog primary sequences for each clade (where X is any amino acid)*

*pX = mean total length of amino acid repeats (AARs) across ortholog primary sequences for each clade (where X is any amino acid)*

*SIM = SIM score*

*REP = REP score*

*LLPS = Σ classifier distance P (ParSE)*

*PRD = PRD score (PLAAC)*
