# Supplementary material for: Divergent evolution of low-complexity regions in the vertebrate CPEB protein family
Source: Front Bioinform. 2025 Mar 20;5:1491735. doi: 10.3389/fbinf.2025.1491735 (PMC11965684; doi:10.3389/fbinf.2025.1491735)
Supplement: Supplementary file 5 [file Table3.pdf]

Supplemental Table 3 - *Pearson's r coefficients* for correlations between the indicated 24 parameters (mean values across either all species per clade, i.e. 'All species', or five randomly selected species per clade, i.e. '5 species - run 1-10') and clade stem ages for CPEB2 orthologs*

Paralogs	% A	% C	% D	% E	% F	% G	% H	% I	% K	% L	% M	% N	% P	% Q	% R	% S	%T	% V	% W	% Y	SIM	REP	LLPS	PRD
5 species - run 1	-0.37	0.233	**0.909**	**0.928**	0.49	-0.41	0.38	**0.98**	0.588	**-0.88**	**0.862**	**0.781**	**-0.94**	-0.33	0.309	0.136	0.08	**0.939**	**0.734**	**0.842**	**-0.82**	**-0.77**	**-0.79**	**-0.78**
5 species - run 2	-0.36	0.274	**0.909**	**0.942**	0.522	-0.45	0.253	**0.976**	0.571	**-0.85**	**0.883**	**0.79**	**-0.94**	-0.42	0.309	0.281	0.134	**0.942**	**0.748**	**0.833**	**-0.81**	**-0.76**	**-0.79**	**-0.8**
5 species - run 3	-0.43	0.271	**0.928**	**0.951**	0.476	-0.38	0.361	**0.959**	0.613	**-0.83**	**0.838**	**0.804**	**-0.96**	-0.33	0.371	0.175	-0.01	**0.961**	0.649	**0.846**	**-0.79**	**-0.74**	**-0.74**	**-0.74**
5 species - run 4	-0.43	0.266	**0.941**	**0.939**	0.599	-0.42	0.432	**0.966**	0.645	**-0.83**	**0.855**	**0.779**	**-0.95**	-0.37	0.356	0.104	-0.05	**0.942**	**0.773**	**0.795**	**-0.79**	**-0.75**	**-0.74**	**-0.7**
5 species - run 5	-0.32	0.17	**0.934**	**0.95**	0.6	-0.26	0.287	**0.988**	0.578	**-0.8**	**0.916**	**0.822**	**-0.97**	-0.33	0.233	0.111	6E-05	**0.968**	**0.689**	**0.847**	**-0.82**	**-0.79**	**-0.78**	**-0.8**
5 species - run 6	-0.44	0.303	**0.93**	**0.957**	0.536	-0.4	0.294	**0.985**	0.625	**-0.88**	**0.838**	**0.796**	**-0.95**	-0.33	0.346	0.092	0.087	**0.952**	**0.686**	**0.817**	**-0.82**	**-0.76**	**-0.77**	**-0.77**
5 species - run 7	-0.41	0.396	**0.927**	**0.93**	0.487	-0.33	0.346	**0.994**	0.643	**-0.82**	**0.845**	**0.818**	**-0.96**	-0.35	0.359	0.211	-0.15	**0.95**	**0.777**	**0.766**	**-0.77**	**-0.75**	**-0.74**	**-0.69**
5 species - run 8	-0.44	0.237	**0.937**	**0.955**	0.529	-0.37	0.286	**0.989**	0.605	**-0.83**	**0.879**	**0.814**	**-0.97**	-0.29	0.237	0.205	0.165	**0.955**	**0.727**	**0.863**	**-0.84**	**-0.8**	**-0.78**	**-0.8**
5 species - run 9	-0.39	0.269	**0.92**	**0.951**	0.557	-0.41	0.316	**0.994**	0.635	**-0.87**	**0.846**	**0.809**	**-0.95**	-0.28	0.318	0.195	-0.1	**0.961**	**0.806**	**0.84**	**-0.83**	**-0.78**	**-0.8**	**-0.81**
5 species - run 10	-0.46	0.352	**0.935**	**0.942**	0.618	-0.42	0.464	**0.985**	0.635	**-0.81**	**0.862**	**0.802**	**-0.95**	-0.41	0.247	0.206	0.097	**0.965**	**0.731**	**0.761**	**-0.83**	**-0.79**	**-0.74**	**-0.81**
All species	-0.43	0.293	**0.929**	**0.947**	0.554	-0.4	0.344	**0.983**	0.634	**-0.85**	**0.868**	**0.8**	**-0.96**	-0.34	0.294	0.149	0.052	**0.957**	**0.785**	**0.815**	**-0.81**	**-0.77**	**-0.78**	**-0.77**

*the *r* value for statistically significant changes is in *green* (increase from older to younger clades) or in *red* (decrease from older to younger clades).

Legend

%X = mean percent amino acid frequency across ortholog primary sequences for each clade (where X is any amino acid)

SIM = SIM score

REP = REP score

LLPS = Σ classifier distance P (ParSE)

PRD = PRD score (PLAAC)
